# Insufficient PD-1 expression during active autoimmune responses: a deep single-cell proteomics analysis in inflammatory arthritis

**DOI:** 10.3389/fimmu.2024.1403680

**Published:** 2024-06-07

**Authors:** Eleni-Kyriaki Vetsika, George E. Fragoulis, Maria Kyriakidi, Kleio-Maria Verrou, Maria G. Tektonidou, Themis Alissafi, Petros P. Sfikakis

**Affiliations:** ^1^ Centre of New Biotechnologies and Precision Medicine (CNBPM), School of Medicine, National and Kapodistrian University of Athens, Athens, Greece; ^2^ First Department of Propaedeutic Internal Medicine and Joint Rheumatology Program, School of Medicine, National and Kapodistrian University of Athens, Athens, Greece; ^3^ Laboratory of Biology, School of Medicine, National and Kapodistrian University of Athens, Athens, Greece; ^4^ Laboratory of Immune Regulation, Center of Basic Sciences, Biomedical Research Foundation Academy of Athens, Athens, Greece

**Keywords:** programmed-cell death-1, mass cytometry, peripheral blood, psoriatic arthritis, rheumatoid arthritis, immune cells, systemic inflammation

## Abstract

**Objectives:**

Programmed cell death protein-1 (PD-1) maintains peripheral immune tolerance by preventing T cell continuous activation. Aiming to understand the extent of PD-1 expression in inflammatory arthritis beyond its involvement with T cells, we assess its presence on various circulating single cells.

**Methods:**

Mass cytometry analysis of patients with active seropositive/seronegative rheumatoid (RA; n=9/8) and psoriatic (PsA; n=9) arthritis versus healthy controls (HC; n=13), re-evaluating patients after 3 months of anti-rheumatic treatment.

**Results:**

PD-1 was expressed in all leukocyte subpopulations, with the highest PD-1^+^ cell frequencies in eosinophils (59-73%) and T cells (50–60%), and the lowest in natural-killer cells (1–3%). PD-1^+^ cell frequencies and PD-1 median expression were comparable between patient subgroups and HC, in the majority of cell subsets. Exceptions included increases in certain T cell/B cell subsets of seropositive RA and specific monocyte subsets and dendritic cells of PsA; an expanded PD-1^+^CD4^+^CD45RA^+^CD27^+^CD28^+^ T subset, denoting exhausted T cells, was common across patient subgroups. Strikingly, significant inverse correlations between individual biomarkers of systemic inflammation (ESR and/or serum CRP) and PD-1^+^ cell frequencies and/or median expression were evident in several innate and adaptive immunity cell subsets of RA and PsA patients. Furthermore, all inverse correlations noted in individuals with active arthritis were no longer discernible in those who attained remission/low disease activity post-treatment.

**Conclusion:**

PD-1 expression may be insufficient, relative to the magnitude of the concomitant systemic inflammatory response on distinct leukocyte subsets, varying between RA and PsA. Our results point to the potential therapeutic benefits of pharmacological PD-1 activation, to rebalance the autoimmune response and reduce inflammation.

## 1 Introduction

Over the past decade, there has been a growing recognition of molecules that play pivotal roles in inhibiting T cell responses, with programmed cell death protein-1 (PD-1) being the most extensively characterized. PD-1 acts as a negative regulator of T cell responses preventing their continuous activation, thus maintaining peripheral immune tolerance and protecting from autoimmunity (1, 2). PD-1 inhibitors have revolutionized oncology where the therapeutic activation of T cells is desired (3, 4). Treatment with these drugs has been shown to reverse exhaustion of PD-1-expressing T cells and even to lead to clonal revival and expansion of precursor T cells (5, 6). While these drugs have shown unprecedented clinical success, they have been associated with immune-related adverse events, (7) including inflammatory arthritis and flares in patients with pre-existing rheumatoid arthritis (RA) (8). Arthralgias and inflammatory arthritis occur in up to 40% and 7.5%, respectively, of patients with malignancies treated with PD-1 inhibitors (9), whereas even in-utero exposure has recently also been linked with (auto)immune-mediated phenomena (10).

On the other hand, aberrations have been reported in the PD-1/programmed cell death ligand-1 (PD-L1) axis in chronic inflammatory arthritis. In collagen-induced arthritis models, the absence or inhibition of PD-1 has been linked to an increased frequency and severity of arthritis (11, 12). Furthermore, PD-1 is upregulated and functional in activated T cells in RA patients’ synovial fluid and synovial tissue, reflecting the continuous local immune activation (11, 13, 14). However, the expression of PD-1 in the peripheral blood of patients with inflammatory arthritis remains essentially unknown, with studies being focused thus far on T cells, reporting conflicting results for both RA and psoriatic arthritis (PsA) (15–19). Moreover, differences in PD-1-mediated interactions between seropositive and seronegative RA have been described (13), possibly reflecting the differences between these two entities (20).

While most studies within the field of rheumatology and beyond have focused on T cells, PD-1 expression on other leukocytes, such as natural killer (NK) cells, dendritic cells (DC), and B cells (21–23) has been documented but remains poorly defined. Importantly, it has been demonstrated that PD-1 expression on other cells can influence T cell status via the PD-1/PD-L1 pathway, for example, PD-1 expression on DCs suppresses CD8^+^ T cell function (22, 23). Moreover, immunocytes other than T and B cells are involved in the pathogenesis of chronic inflammatory arthritis (24, 25).

Our objective was to assess PD-1 expression beyond its involvement with T cells in autoimmune responses, by performing a deep single-cell analysis in the peripheral blood of patients with seropositive or seronegative active RA, patients with active PsA, and healthy donors. In this context, we also aimed to correlate the presence of PD-1 with the disease activity during autoimmune responses in active inflammatory arthritis.

## 2 Materials and methods

### 2.1 Patients

A total of 17 biologic treatment-naïve patients with active RA (RA 2010 criteria), of whom nine were seropositive (ACPA and/or RF positive) and eight were seronegative (ACPA and RF negative), as well as nine biologic treatment-naïve patients with active PsA (CASPAR criteria) who attended outpatient rheumatology clinics from March to June 2022, were consecutively enrolled. The clinical patient characteristics are presented in **Table 1**. Patients with a history of neoplasm (solid or hematological), recent infection, and those who had been vaccinated within the previous four weeks were excluded. Healthy individuals (n=13), age and sex-matched, served as controls (HC).

**Table 1:** Demographics and clinical data of study participants.

Characteristics	HC, n=13 n, (%)	Seropositive RA, n=9 n, (%)	Seronegative RA, n=8 n, (%)	PsA, n=9 n, (%)
Median Age				
Years (range)	51 (38-60)	56 (33-65)	50 (23-77)	49 (19-55)
Gender				
Male	4 (30.8)	1 (11.1)	0 (0)	3 (33.3)
Female	9 (69.2)	8 (88.9)	8 (100)	6 (66.7)
Smoking Status				
Active smoker	4 (30.8)	1 (11.1)	4 (50)	2 (22.2)
No-smoker	9 (69.2)	8 (88.9)	4 (50)	7 (77.8)
Treatment				
Naive	N/A	3 (33.3)	3 (37.5)	5 (55.6)
csDMARDs-experienced				
Methotrexate	N/A	5 (55.6)	4 (50)	4 (44.4)
Leflunomide	N/A	1 (11.1)	1 (12.5)	0 (0)
Laboratory parameters				
CRP, mg/l [mean (± SEM)]	1.2 (0.3)	40.1 (28.7)	7.0 (1.7)	10.1 (3.0)
ESR, mm/h [mean (± SEM)]	12 (1.3)	44 (8.5)	32 (8.3)	29 (6.4)
RF positivity	N/A	9 (100)	N/A	N/A
ACPA positivity	N/A	7 (77.8)	N/A	N/A
Disease activity score	N/A	DAS28	DAS28	DAPSA
mean (± SEM)	N/A	5.4 (0.33)	4.7 (0.17)	17.3 (1.14)
Axial involvement	N/A	N/A	N/A	3 (33%)
Enthesitis	N/A	N/A	N/A	1 (11%)
BSA (≥ 3)	N/A	N/A	N/A	4 (44%)

n, number; RA, rheumatoid arthritis; PsA, psoriatic arthritis; HC, healthy controls, CRP, C-reactive protein; ESR, erythrocyte sedimentation rate; csDMARDs, conventional synthetic disease-modifying antirheumatic drugs; csDMARDs-experienced, patients who have already received csDMARDs at the time of enrolment; RF, rheumatoid factor, ACPA, anti-citrullinated peptide antibodies; DAPSA, Disease Activity Index for Psoriatic Arthritis; DAS28, Disease Activity Score-28, BSA, body surface area.

Moderate/high disease activity for RA (active RA) was defined as DAS28> 3.2 and for PsA (active PsA) as DAPSA> 14 and/or ASDAS> 1.2 (for those having axial disease confirmed by X-rays or magnetic resonance). Matched synovial fluid (SF) samples from two RA patients with knee arthritis were simultaneously collected. Subsequent blood samples were obtained three months after the introduction or change of antirheumatic treatment from seven seropositive RA, two seronegative RA, and eight PsA patients. At the 3-month time-point, patients received treatments as follows: methotrexate monotherapy (n=1), anti-TNF (n=4) and JAK inhibitors (n=2), seropositive RA patients; methotrexate monotherapy (n=1) and steroid (n=1), seronegative patients; methotrexate monotherapy (n=3), leflunomide (n=1), apremilast (n=1), anti-TNF (n=1) and anti-IL-17 (n=2), PsA patients. All these patients were treated for active disease in the context of standard clinical practice and according to national and international guidelines.

The study complied with the Ethical Principles for Medical Research Involving Human Subjects according to the World Medical Association Declaration of Helsinki and the Oviedo Convention and was approved by the local Ethics and Scientific Committees of the University Hospitals of the National and Kapodistrian University of Athens (No.314/2021). All individuals signed the informed consent form.

### 2.2 Whole blood staining

One ml of whole blood or SF was incubated with heparin (at a final concentration of 100 U/ml) for 20 min at room temperature (RT). Then, 270 μl of heparin-blocked blood or SF were transferred directly into the 31 antibody cocktail (Maxpar Direct Immunophenotyping Assay (201334) supplemented with the anti-PD-1, **Supplementary Table 1**; Standard BioTools Inc., San Francisco, CA, USA) for 30 min at RT, followed by red blood cell lysis using 250 μl of Cal-Lyse lysing solution (Thermo Fisher Scientific, Waltham, MA, USA) for 10 min at RT in the dark. After incubation with 3 ml of Maxpar Water for 10 min at RT in the dark, the tube was centrifuged at 300 x g for 5 min and the supernatant was carefully aspirated. Cells were washed 3 times using 3 ml of Maxpar Cell Staining Buffer (CSB). Next, cells were fixed and permeabilized by adding 1 ml of 1.6% formaldehyde solution in Maxpar Phosphate Buffer Solution (PBS). After incubating for 10 min at RT, the cells were centrifuged at 800 x g for 5 min and DNA staining was performed by overnight incubation in 1 ml of 125 nM Cell-ID Iridium intercalator solution (Standard BioTools Inc., San Francisco, CA, USA) in Maxpar Fix and Perm Buffer (Standard BioTools Inc., San Francisco, CA, USA) at 4°C. Cells were then washed, pelleted, and kept at -80°C until acquisition.

### 2.3 Sample acquisition

A 3^rd^ generation Helios mass cytometer was used for sample acquisition. Prior to the acquisition, samples were washed twice with 1 ml of Maxpar Cell Staining Buffer (CSB) and twice with 1 ml of Cell Acquisition Solution (CAS; Standard BioTools Inc., San Francisco, CA, USA). Then, the cells were resuspended in CAS containing 0.1X EQ Four Element Calibration Beads (Standard BioTools Inc., San Francisco, CA, USA) to obtain a cell concentration of 250,000-500,000 cells per ml and filtered through a 35-mm nylon mesh cell strainer (BD Biosciences). Using the CyTOF Software version 7.0.8493, a minimum of 500,000 events were acquired per file, at a rate of 200–300 events/second on a Helios mass cytometer (Standard BioTools Inc., San Francisco, CA, USA).

### 2.4 Data analysis

After completion of the acquisition, data were converted to FCS, randomized, and normalized files using beads passport by the CyTOF software (Version, 7.0.8493) and then Cytobank (Beckman Coulter Life Sciences, Indianapolis, IN, USA) were used for analyzing our data, manual biaxial gating on clean live single cells, and visualization of t-distributed stochastic neighbor embedding (tSNE). The gating strategy for the initial preprocessing stages and the definition of the subpopulations/subsets are shown in **Supplementary Figures 1**-**14**. Granulocytes, monocytes, NK, T cells, CD4^+^T cells, CD8^+^T cells, B cells, DC, γδT cells, mucosal-associated invariant T/invariant NKT (MAIT/iNKT) cells, and innate lymphoid cells (ILC) are referred to as subpopulations throughout the paper. Neutrophils, eosinophils, CD66b^-^neutrophils, monocytes [classical, transitional, and non-classical], NK [early and late], double negative (DN) T cells, CD8^+^T [naïve, central memory (CM), effector memory (EM), terminal effector (TE), CD27^-^CD8^-^, senescent T cells (Tsen), Tsen/effector memory T cells re-expressing CD45RA (Temra), CD27^+^CD8^+^, CD127^+^CD27^+^CD8^+^, and CD45RA^+^CD27^+^CD8^+^], CD4^+^T [naïve, CM, EM, TE, CD27^-^CD8^-^, Tsen, Tsen/Temra, CD27^+^CD8^+^, CD127^+^CD27^+^CD8^+^, and CD45RA^+^CD27^+^CD8^+^, regulatory T cells (Treg), T helper cells-1 (Th1), Th2, Th17, T follicular helper (Tfh), and T peripheral helper (Tph)], B [naïve, memory, IgD^+^ memory, IgD^-^ memory, and age-associated B cells (ABC)], mDC, pDC, activated MAIT/iNKT, memory MAIT/iNKT, ILC2 and ILC3 are referred to as subsets throughout the paper. The phenotypes of the identified subpopulations/subsets are listed in online **Supplementary Table 2**.

The raw median values (MI) were exported from Cytobank and were used to quantify PD-1 expression on a single cell level. For visualization of the high-dimensional data on two dimensions, the dimensionality reduction algorithm viSNE (t-distributed stochastic neighbor embedding–based visualization) was performed using 466,624 CD45^+^PD-1^+^ cells, with theta set to 0.5, a perplexity of 30 and maximum number of iterations equal to 3,000.

### 2.5 Statistical analysis

Because of the observational/exploratory nature of the study, it was not possible to propose a statistical hypothesis for the estimation of the appropriate sample size. Statistical analysis was performed using GraphPad Prism version 9.0 (GraphPad Software, San Diego, CA, USA). Normal distribution was assessed by the D’Agostino-Pearson test with a 0.05 alpha value. Comparisons of percentages and MI were conducted with unpaired t-test or Mann-Whitney U test. The differences between the four cohorts’ PD-1^+^-cell subpopulations were drawn in R utilizing the `ggplot2` library. Groups, according to disease activity and different timepoint, were compared by performing two-way ANOVA corrected for multiple comparisons by false discovery rate (FDR) using the two-stage linear step-up procedure of Benjamini, Krieger and Yekutieli. To search for correlations between individual biomarkers of systemic inflammation [erythrocyte sedimentation rate (ESR) and/or serum C-reactive protein (CRP)] and PD-1^+^ cell frequencies and/or median expression in cell subpopulation/subsets, the `cor. test` function in R was utilized. (R version 3.6.2). Differences and correlations were considered significant when *p*- or *q-*value < 0.05 and for two-sided tests.

## 3 Results

### 3.1 PD-1 is heterogeneously expressed across all leukocyte subsets in peripheral blood and synovial fluid

**Figure 1:**
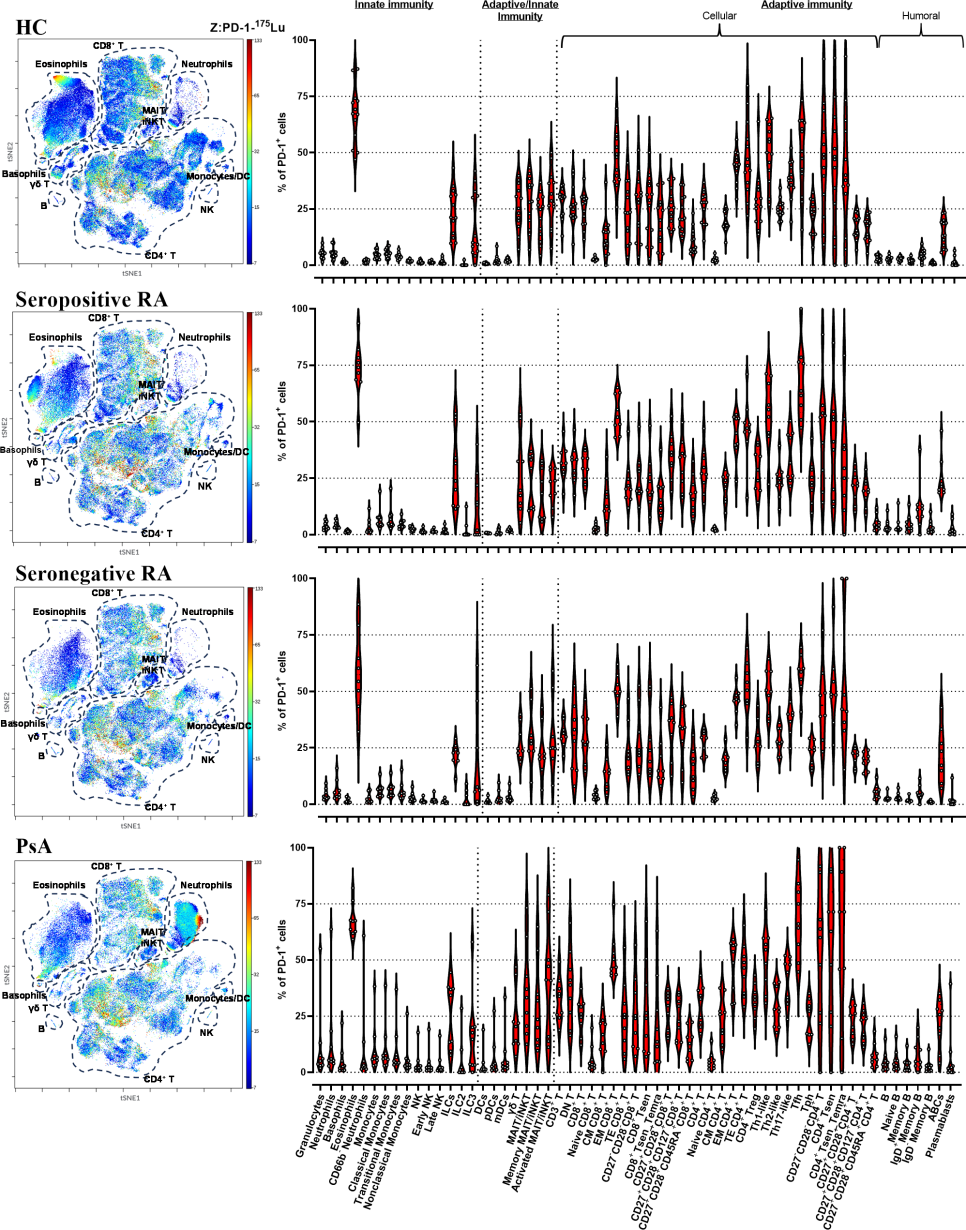
CyTOF analysis shows PD-1 expression in all circulating leukocytes. viSNE plots showing 466,624 PD-1-expressing single cells (HC=13; seropositive RA=9; seronegative RA=8; PsA=9). The primary cell populations are circled with a dotted line on the viSNE plot. Each dot represents a cell and is colored according to PD-1 intensity on a spectrum heat scale (red = high intensity; blue = low intensity). Arcsine-transformed color scales report the raw values of the PD-1’s intensity. Corresponding violin plots showing the frequencies of the PD-1^+^ cells in 59 leukocyte subpopulations/subsets studied. (HC, healthy control; PsA, psoriatic arthritis; RA, rheumatoid arthritis).

A deep single-cell analysis was conducted to assess the presence and quantification of circulating PD-1^+^ leukocytes in biologic treatment-naive patients with inflammatory arthritis, utilizing mass cytometry. As depicted in **Figure 1**, PD-1 exhibited a heterogenous expression pattern across all 59 leukocyte subpopulations/subsets studied in healthy controls, seropositive RA, seronegative RA, and PSA. The highest frequencies of PD-1^+^ cells were observed in eosinophils (59-73%), followed by T cells, exhibiting a range of 50-60%, while NK cells exhibited the lowest PD-1 expression frequencies, ranging from 1% to 3% (**Figure 1**).

A heterogeneous expression pattern of PD-1 across all leukocyte subpopulations/subsets studied was also noted in synovial fluid from two RA patients. Notably, frequencies of PD-1^+^ cells were found to be elevated in synovial fluid compared to the corresponding blood samples across the majority of examined cell subpopulations/subsets (**Supplementary Figure 16**).

### 3.2 Distinct PD-1^+^ profiling of T and B cells in active seropositive RA

Analyzing PD-1 expression on 31 T cell and 6 B cell subpopulations/subsets in patients with active inflammatory arthritis in comparison to HC, several differences were found that pertained almost exclusively to seropositive RA (**Supplementary Tables 3**, **4**).

**Figure 2:**
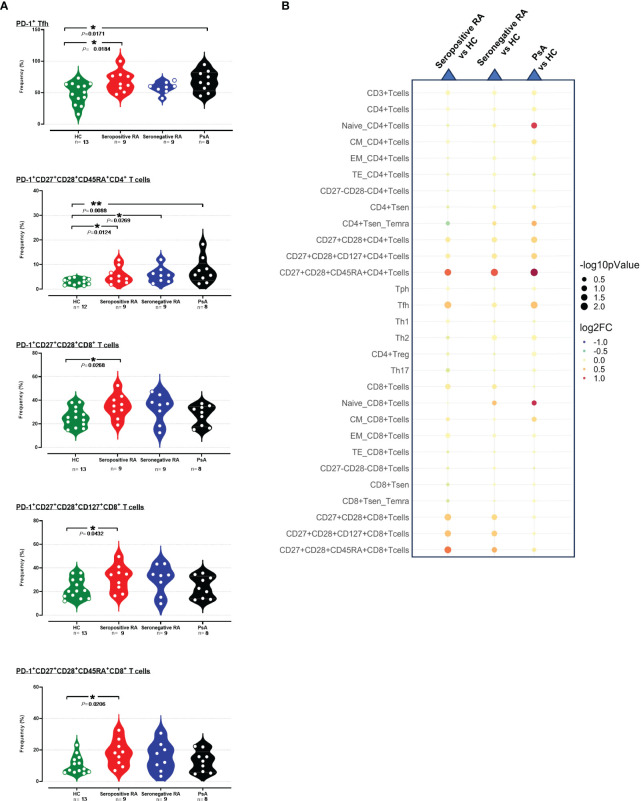
**(A)** Violin plots showing the frequencies of PD-1^+^Tfh, PD-1^+^CD27^+^CD28^+^CD45RA^+^CD4^+^ T cells, PD-1^+^CD27^+^CD28^+^CD8^+^ T cells, PD-1^+^CD27^+^CD28^+^CD127^+^CD8^+^ T cells and PD-1^+^CD27^+^CD28^+^CD45RA^+^CD8^+^ T cells, across the four subgroups. **(B)** Integrated heatmap/dot-plot showing fold change and p-value between the three patients’ subgroups and the HC of the cellular immunity PD-1^+^ T cell subsets’ frequencies. Each white circle at the violin plots corresponds to an individual patient (green plot = HC, n = 13; red plot = seropositive RA patients, n = 9; blue plot = seronegative RA patients, n = 8; black plot = PsA patients, n = 9). Asterisks indicate statistically significant differences between groups based on unpaired t-test or Mann–Whitney U test. (n, number of patients; HC, healthy controls; PsA, psoriatic arthritis; RA, rheumatoid arthritis; CM, central memory; EM, effector memory; TE, terminal effector; Tsen, senescent T cells; Temra, effector memory T cells re-expressing CD45RA; Treg, regulatory T cells; Th, T helper cells; Tfh, T follicular helper, Tph, T peripheral helper; *, p ≤ 0.05 and **, p ≤ 0.01). The size of the dots at the dot-blots corresponds to the statistical significance, with bigger dots denoting lower p-values. Coloring corresponds to log2 Fold Change, with red denoting a higher abundance in the examined group, while blue denotes a higher abundance in the baseline group.

In detail, the levels of PD-1 median expression on single CD3^+^ cells, as well as on single CD4^+^ T cells were higher in seropositive RA patients, but not in seronegative RA or PsA, compared to HC (**Supplementary Table 3**, **Supplementary Figure 17**). Among the various cytotoxic CD8^+^ T cell subsets, significant increases of both PD-1^+^ cell frequencies and PD-1 median expression were noted in total CD27^+^CD28^+^ activated cells, as well as in those expressing CD127^+^ or CD45RA^+^ (**Figures 2A, B**, **Supplementary Figure 17**). PD-1 expression levels (but not their frequencies) were also elevated on PD-1^+^EM CD8^+^ T cells in seropositive RA compared to HC (**Supplementary Figure 17**, **Supplementary Tables 3**, **4**). Similarly, among the various CD4^+^ T cell subsets, levels of the PD-1 molecule on CD27^+^CD28^+^ cells, as well as those expressing CD127^+^ or CD45RA^+^, were higher in seropositive RA patients compared to HC (**Supplementary Table 3**, **Supplementary Figure 17**). In addition, the expression of the PD-1 molecule on EM CD4^+^ T cells, and Th1-like cells was augmented in seropositive RA patients compared to HC (**Supplementary Figure 17**, **Supplementary Table 4**). Intriguingly, an expanded PD-1^+^CD4^+^CD45RA^+^CD27^+^CD28^+^ subset denoting exhausted T helper cells was common across both seropositive and seronegative RA, as well as PsA (**Figures 2A, B**). Another interesting finding was that the frequencies of PD-1^+^Tfh cells were higher in both seropositive RA and PsA compared to HC (**Supplementary Table 3**).

**Figure 3:**
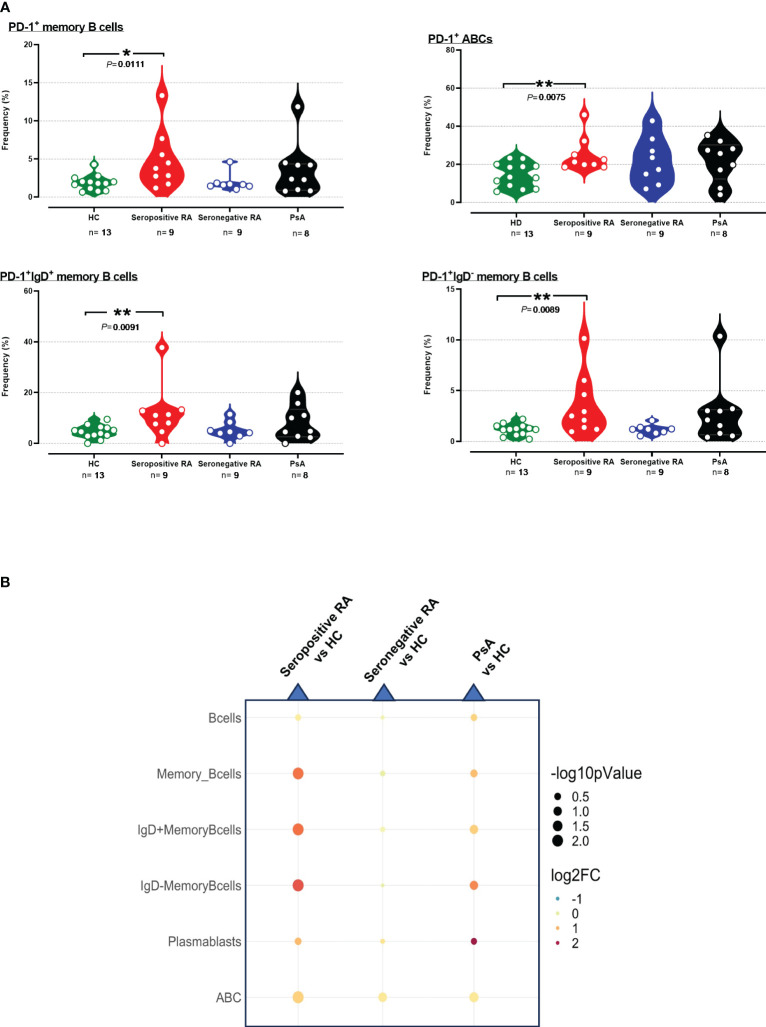
**(A)** Violin plots showing the frequencies of PD-1^+^memory B cells, PD-1^+^IgD^+^ B cells, PD-1^+^IgD^-^ B cells, and PD-1^+^ABC cells, across the four subgroups. **(B)** Integrated heatmap/dot-plot showing fold change and p-value between the three patients’ subgroups and the HC of humoral immunity PD-1^+^ B subsets’ frequencies. Each white circle at the violin plots corresponds to an individual patient (green plot = HC, n = 13; red plot = seropositive RA patients, n = 9; blue plot = seronegative RA patients, n = 8; black plot = PsA patients, n = 9). Asterisks indicate statistically significant differences between groups based on unpaired t-test or Mann–Whitney U test. (n, number of patients; HC, healthy controls; PsA, psoriatic arthritis; RA, rheumatoid arthritis; *, p ≤ 0.05 and **, p ≤ 0.01). The size of the dots at the dot-blots corresponds to the statistical significance, with bigger dots denoting lower p-values. Coloring corresponds to log2 Fold Change, with red denoting a higher abundance in the examined group, while blue denotes a higher abundance in the baseline group.

PD-1^+^ frequencies were also systematically assessed across various cell subtypes involved in humoral immunity. As observed in T cells, differences in PD-1^+^ profiling among B cells and their subsets compared to HC were detected only in seropositive RA and not in seronegative RA or PsA (**Supplementary Tables 3**, **4**). Indeed, an elevated PD-1 expression on total PD-1^+^B cells was noted in these patients (**Supplementary Figure 18**, **Supplementary Table 4**). Moreover, significant increases of both PD-1^+^ cell frequencies and PD-1 median expression were noted in PD-1^+^ memory B cells in seropositive RA; increased PD-1^+^ cell frequencies were also noted among both IgD^+^ and IgD^-^ memory B cell subsets, as well as in ABCs (**Figures 3A, B**, **Supplementary Table 3**).

### 3.3 Distinct PD-1^+^ profiling of innate immunity cells and cells “bridging” innate and adaptive immunity in active PsA

**Figure 4:**
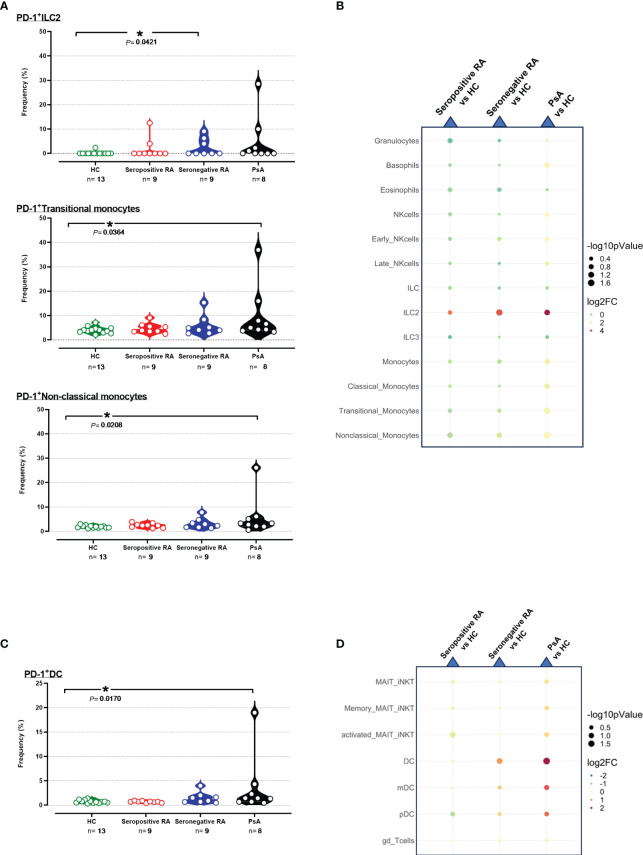
**(A)** Violin plots showing the frequencies of PD-1^+^ ILC2, PD-1^+^ Transitional, and Non-classical monocytes across the four cohorts. **(B)** Integrated heatmap/dot-plot showing fold change and p-value between the three patients’ cohorts and the HC of the innate immunity PD-1^+^ immune cell subsets’ frequencies. **(C)** Violin plots showing the frequencies of PD-1^+^ DC across the four cohorts. **(D)** Integrated heatmap/dot-plot showing fold change and p-value between the three patients’ cohorts and the HC of the innate/adaptive immunity PD-1^+^ immune cell subsets’ frequencies. Each white circle at the violin plots corresponds to an individual patient (green plot = HC, n = 13; red plot = seropositive RA patients, n = 9; blue plot = seronegative RA patients, n = 8; black plot = PsA patients, n = 9). Asterisks indicate statistically significant differences between groups based on unpaired t-test or Mann–Whitney U test. (n, number of patients; HC, healthy controls; PsA, psoriatic arthritis; RA, rheumatoid arthritis; ILC2, innate lymphoid cells; DC, dendritic cells; *, p ≤ 0.05). The size of the dots at the dot-plots corresponds to the statistical significance, with bigger dots denoting lower p-values. Coloring corresponds to log2 Fold Change, with red denoting a higher abundance in the examined group, while blue denotes a higher abundance in the baseline group.

The frequencies of PD-1^+^ cells and the median PD-1 expression in 15 subpopulations of innate immunity, along with 7 subpopulations bridging innate and adaptive immunity, were similar between seropositive RA and healthy controls. Concerning seronegative RA, comparable results to HC were observed (**Supplementary Tables 5**, **6**), except for elevated PD-1^+^ILC2 frequencies (**Figures 4A, B**).

Interestingly, significant increases of PD-1^+^ cell frequencies and/or PD-1 median expression in 5/15 innate immunity and 3/7 subpopulations/subsets bridging innate and adaptive immunity were observed in PsA patients versus HC (**Supplementary Tables 5**, **6**). As shown in **Figure 4**, higher frequencies of transitional PD-1^+^ monocytes and non-classical PD-1^+^ monocytes were evident in PsA compared to HC, whereas PD-1 expression levels on these two monocyte subsets were also increased (**Figure 4A, B**, **Supplementary Figures 19A, B**). Moreover, increased PD-1 expression in PsA was noted in neutrophils, total monocytes, and classical monocytes (**Supplementary Figures 19A, B**). Regarding the cells bridging innate and adaptive immunity, PD-1^+^DC exhibited higher frequencies in PsA compared to HC (**Figures 4C, D**). In contrast, higher expression levels of PD-1 were noted on both pDC and mDC subsets (**Supplementary Figures 20A, B**, **Supplementary Table 6**).

### 3.4 Systemic inflammation biomarkers and PD-1^+^ profiling of specific circulating leukocyte subsets in active RA and PsA

To indirectly assess the hypothesis that PD-1 signaling may be deficient during autoimmune responses occurring in active inflammatory arthritis, we searched for correlations between biomarkers of systemic inflammation and the frequencies and/or the expression of PD-1 on circulating leukocytes among patients. Indeed, significant correlations between individual values of ESR and/or serum CRP and PD-1^+^ cell frequencies and/or median expression were evident in several of the 59 innate/adaptive immunity cell subpopulations/subsets, both in seropositive (11/59; **Table 2A**, **Supplementary Table 7A**) and seronegative RA (6/59; **Table 2B**, **Supplementary Table 7B**), as well as in PsA (25/59; **Table 2C**, **Supplementary Table 7C**). Strikingly, most of these correlations were inverse (5/11, 5/6, and 24/25, respectively). Conversely, ESR and/or serum CRP displayed a positive correlation with the PD-1^+^CD4^+^ Treg across both seropositive and seronegative RA, as well as in PsA (**Table 2**, **Supplementary Table 7**).

**Table 2:** Significant correlations of the frequencies of circulating PD-1^+^ leukocytes with CRP and/or ESR in bDMARD-naïve, (A) seropositive RA, (B) seronegative RA patients, and (C) PsA patients.

Subpopulations/subsets of PD-1^+^-circulating leukocytes	CRP		ESR	
	Rho	P value	Rho	P value
A. SEROPOSITIVE RA				
Innate Immunity				
Neutrophils	**0.783**	**0.017**	0.452	NS
Adaptive Immunity				
CD8^+^ T cells				
CM	**0.678**	**0.045**	0.550	NS
B cells	**-0.700**	**0.043**	-0.594	NS
Naïve	**-0.717**	**0.037**	**-0.703**	**0.035**
IgD^+^ Memory B cells	**-0.800**	**0.014**	-0.343	NS
B. SERONEGATIVE RA				
Innate Immunity				
ILC	-0.595	NS	**-0.833**	**0.015**
ILC3	**-0.810**	**0.022**	**-0.762**	**0.037**
Adaptive Immunity				
DN T cells	**-0.810**	**0.022**	**-0.857**	**0.011**
CD4^+^ Treg	**0.738**	**0.046**	0.595	NS
C. PsA				
Innate Immunity				
Monocytes	**-0.767**	**0.021**	**-0.833**	**0.008**
Classical	**-0.767**	**0.021**	**-0.833**	**0.008**
Transitional	**-0.750**	**0.025**	**-0.800**	**0.014**
Basophils	-0.633	NS	**-0.700**	**0.043**
CD66b^-^ Neutrophils	**-0.717**	**0.037**	-0.650	NS
Innate & Adaptive Immunity				
DC				
Myeloid	**-0.717**	**0.037**	-0.650	NS
Adaptive Immunity				
CD3^+^ T cells	-0.650	NS	**-0.750**	**0.025**
CD4^+^ T cells	**-0.717**	**0.037**	**-0.850**	**0.006**
Naïve	**-0.767**	**0.021**	**-0.817**	**0.011**
CM	-0.633	NS	**-0.867**	**0.005**
EM	**-0.700**	**0.043**	**-0.850**	**0.006**
TE	**-0.733**	**0.031**	**-0.883**	**0.003**
Treg	-0.400	NS	**-0.683**	**0.050**
Th1-like	-0.500	NS	**-0.700**	**0.043**
Th2-like	-0.417	NS	**-0.867**	**0.005**
Tph	**-0.683**	**0.050**	**-0.800**	**0.014**
CD27^+^CD28^+^	-0.617	NS	**-0.917**	**0.001**
CD27^+^CD28^+^CD127^+^	-0.517	NS	**-0.883**	**0.003**
CD45RA^+^CD27^+^CD28^+^	-0.567	NS	**-0.783**	**0.017**
CD27^-^CD28^-^	**-0.850**	**0.006**	**-0.783**	**0.017**
Tsen	**-0.828**	**0.006**	**-0.770**	**0.015**
Tsen/Temra	**-0.748**	**0.020**	**-0.798**	**0.010**
CD8^+^ T cells				
Naïve	**-0.700**	**0.043**	**-0.717**	**0.037**
Plasmablasts	**-0.746**	**0.021**	**-0.797**	**0.010**

Correlation coefficients between the variables were calculated according to Spearman’s rank correlation coefficient (rho).

RA, rheumatoid arthritis; PsA, psoriatic arthritis; CRP, C-reactive protein; ESR, erythrocyte sedimentation rate; PD-1, programmed cell death-1; ILC3, innate lymphoid cells type 3; Treg, regulatory T cells; CM, central memory; DC, dendritic cells; EM, effector memory; TE, terminal effector; Th, T helper cells; Tph, T peripheral helper; Tsen, senescent T cells; Temra, effector memory T cells re-expressing CD45RA.

The bold values denote statistical significance.

In detail, regarding seropositive RA patients, individual CRP levels were inversely correlated with frequencies of PD-1^+^ B cells, as well as their subtypes, namely IgD^+^ memory and naïve B cells; the latter were also inversely correlated with ESR (**Table 2A**). Moreover, an inverse correlation was observed between CRP and the MI of PD-1 on ILC and memory B cells (**Supplementary Table 7A**). On the other hand, individual CRP levels were positively correlated with frequencies of PD-1^+^ CM CD8^+^ T cells (**Table 2A**). ESR showed an inverse correlation with the expression levels of PD-1 on ILCs, while positive correlations were observed on neutrophils, CD4^+^, and CD8^+^ T cell subtypes (**Supplementary Table 7A**).

As far as the seronegative RA patients were concerned, both individual ESR and CRP levels were inversely correlated with the frequencies of PD-1-expressing immune cells bridging innate and adaptive immunity, such as ILC3, as well as with DN T cells (**Table 2B**). Not surprisingly, a positive correlation was observed between CRP and both the frequency of PD-1^+^CD4^+^ Treg and PD1 expression levels in this particular subset (**Table 2B**, **Supplementary Table 7B**). Furthermore, an inverse correlation was found between both CRP and ESR and the expression levels of PD-1 on PD-1^+^ ILC and NK cells (**Supplementary Table 7B**).

Regarding PsA, as shown in **Table 2C** individual CRP levels displayed inverse correlations with most of the frequencies of PD-1-expressing cells involved in innate and acquired immunity. These inverse correlations included CD66b^-^ neutrophils, monocytes, classical and transitional monocytes, mDC, naïve CD8^+^ T cells, CD4^+^ T cells, and their subtypes (naïve, EM, TE, Tph, CD27^-^CD28^-^, Tsen, Tsen/Temra), and plasmablasts. Similarly, many inverse correlations were also observed between ESR and the frequencies of various PD-1-expressing cell subtypes of innate and acquired immunity (basophils, monocytes, classical monocytes, transitional monocytes, CD3^+^ T cells, all CD4^+^ T cells subtypes, naïve CD8^+^ T cells and plasmablasts (**Table 2C**).

Moreover, in PsA patients, CRP was inversely correlated with the expression levels of PD-1 on PD-1-expressing eosinophils, DC, CD4^+^ Tsen/Temra, and naïve CD8^+^ T cells. At the same time, it showed a positive correlation with PD-1 expression on CD27^+^CD28^+^CD8^+^ T cells (**Supplementary Table 7C**). An inverse correlation was also observed between ESR and the expression levels of PD-1 on eosinophils, monocytes, classical monocytes, CD3^+^ T cells, and various CD4^+^ T cell subtypes (**Supplementary Table 7C**).

### 3.5 Abrogation of inverse correlations between systemic inflammation biomarkers and PD-1 expression in RA/PsA patients achieving remission/low disease activity

We re-evaluated a total of 17 of 26 patients studied at baseline (seven, two, and eight patients with seropositive RA, seronegative RA, and PsA, respectively), after three months of antirheumatic treatment, as per routine clinical practice. Of these 17 patients, nine patients achieved remission/low disease activity, while the remaining eight patients had persistently active RA or PsA, albeit less active than baseline, despite treatment.

Due to the small numbers, we analyzed patients collectively, irrespective of the type of inflammatory arthritis. We evaluated the changes in PD-1^+^ immune cell subpopulations/subsets during treatments after dividing our IA patients into those who achieved remission/low disease activity and those with persistently active disease. The frequencies of PD-1^+^ cells remained comparable between baseline and after three months of treatments across all, but one (PD-1^+^Th17-like), circulating leukocyte subpopulations/subsets, irrespective of clinical outcome (**Supplementary Figure 21**). Furthermore, higher baseline levels of PD-1 expression were detected on PD-1-expressing naïve CD4^+^ T cells, Tfh, CM and EM CD8^+^ T cells in patients who achieved remission/low disease activity, in contrast to those with persistent active disease (**Supplementary Figure 22**). Interestingly, we found that among patients who achieved either disease remission or low disease activity all inverse associations observed between systemic inflammation biomarkers and PD-1^+^-expressing cells at baseline disappeared (**Table 3**, **Supplementary Table 8**). Conversely, in patients with sustained active disease, CRP correlated inversely with frequencies of certain circulating PD-1^+^CD4^+^ T cell subtypes (CD27^-^CD28^-^, Tsen, Tsen/Temra). In contrast, various cells of innate/adaptive immunity showed inverse correlations with ESR (**Table 3**).

**Table 3:** Significant correlations of the frequencies (%) of circulating PD-1^+^ leukocytes with CRP and/or ESR in IA patients (A) who remained active 3 months post-therapy (n=8), (B) who became inactive 3 months post-therapy (n=9).

Subpopulations/subsets of PD-1^+^-circulating leukocytes	CRP		ESR	
	Rho	P value	Rho	P value
A. Active disease 3 months post-therapy				
Innate Immunity				
ILC				
ILC2	-0.655	NS	**-0.741**	**0.035**
Granulocytes	-0.214	NS	**-0.743**	**0.035**
Neutrophils	-0.238	NS	**-0.814**	**0.014**
CD66b^-^ Neutrophils	-0.262	NS	**-0.778**	**0.023**
Innate & Adaptive Immunity				
DC	-0.262	NS	**-0.778**	**0.023**
Myeloid	-0.238	NS	**-0.719**	**0.045**
Adaptive Immunity				
CD4^+^ T cells				
CD27^-^CD28^-^	**-0.922**	**0.001**	-0.199	NS
Tsen	**-0.970**	**0.001**	-0.452	NS
Tsen/Temra	**-0.874**	**0.005**	-0.524	NS
Plasmablasts	-0.084	NS	**-0.807**	**0.015**
B. Inactive disease 3 months post-therapy				
Eosinophils	**0.678**	**0.045**	0.552	NS

Correlation coefficients between the variables were calculated according to Spearman’s rank correlation coefficient (rho).

IA, inflammatory arthritis; CRP, C-reactive protein; ESR, erythrocyte sedimentation rate; PD-1, programmed cell death-1; ILC, innate lymphoid cells; DC, dendritic cells; Tsen, senescent T cells; Temra, effector memory T cells re-expressing CD45RA.

The bold values denote statistical significance.

## 4 Discussion

Herein, we show that PD-1, in addition to T cells (13, 17, 18, 26) is essentially expressed in every circulating leukocyte subset of either innate or adaptive immunity. These findings are derived from the first deep single-cell analysis by mass cytometry exploring PD-1 expression in the peripheral blood of RA and PsA patients versus healthy controls. The distinctive pathogenetic mechanisms in seropositive and seronegative RA (27–29) led us to present our findings separately for these subsets.

Unexpectedly, the highest expression of PD-1 in peripheral blood was noted on eosinophils, probably implying a physiological need for tightly regulated responses of these cells in health and autoimmune disease. Of note, the role of eosinophils is not very well examined in inflammatory arthritis so far. There is some evidence however that a subset of them might alleviate inflammation in arthritic joints (30). Further studies are warranted to examine the precise PD-1 regulatory function, if any, in these cells. On the other hand, the lowest expression of PD-1 was noted on NK cells; the absence of PD-1 expression in almost 97% of these cells may also suggest a mandatory physiological need for minimal negative regulation, contrary to eosinophils, for these particular immunocytes.

Consistent with the fact that the function of immune checkpoints should be enhanced in patients with active RA or PsA to protect from the uncontrolled inflammatory response, we found that PD-1 expression is increased in some peripheral blood subsets both in RA and PsA, compared to HC, albeit in a distinct pattern. Previous flow-cytometry studies primarily examined PD-1 expression in peripheral blood T cells in RA, yielding conflicting results, especially in CD4^+^T and CD8^+^ T cell populations with most of the evidence, however, supporting the increased expression of PD-1 compared to healthy individuals or patients with osteoarthritis (14, 15, 17, 31). Interestingly, using mass cytometry in exclusively bDMARD-naïve patients with active seropositive RA, we observed elevated frequencies of PD-1-expressing cells across various T cell and B cell subtypes. Seronegative RA displayed this trend only in ILC2 and CD45RA^+^CD27^+^CD28^+^CD4^+^ T cells (32). For this particular cell subset, we observed that PD1^+^ cell frequencies were increased across all types of inflammatory arthritis, suggesting their potential common role. In contrast, we show for the first time that for PsA, increased frequencies of cells expressing PD-1 were noted mainly in cells of innate immunity as well as in those bridging innate and adaptive immunity, corroborating the hypothesis that these cells are important in the pathogenesis of this disease (25, 27).

Besides, we had the opportunity to perform a single-cell analysis of PD-1 expression in paired blood and knee-derived synovial fluid samples from two RA patients. In agreement with previous flow cytometry studies, PD-1 expression was increased in synovial fluid lymphocytes compared to the blood lymphocytes in RA (33–35). We also found increased PD-1 expression in synovial fluid relative to paired peripheral blood in all lymphocyte subsets in both patients, as well as in the majority of innate immunity cell subsets. These novel findings underscore the fact that synovial fluid immunocytes, situated in the inflammatory milieu proximal to the target tissue, exhibit an exhausted phenotype due to sustained activation compared to their circulating counterparts.

Finally, we sought to indirectly investigate whether PD-1 signaling during autoimmune responses in patients with active inflammatory arthritis may be inadequate. Indeed, we found that the individual degree of the systemic inflammatory response as attested by elevated ESR and CRP values, had a striking inverse relationship with expression levels of PD-1 in various leukocyte subtypes across the three groups of patients with inflammatory arthritis. The pattern of active seronegative RA was quite similar to that observed for active PsA, while seropositive RA differed, demonstrating inverse correlations mainly for B cell subsets. This is not unexpected given that the B cell is one of the major effector cells in seropositive RA in terms of producing autoantibodies. Notably, Zacca et al. (36) observed increased levels of PD-L1-expressing B cells in RA patients, associated with reduced disease activity following treatment. Their findings suggest a potential role for the PD-L1/PD-1 pathway in modulating disease activity in patients with RA. Further substantiating that PD-1 expression may be insufficient in active inflammatory arthritis, all inverse correlations between PD-1 expression levels and ESR/CRP levels disappeared in those patients who achieved low disease activity after three months of treatment, in contrast to those who remained active, in whom several inverse correlations were again observed. These findings collectively offer possible explanations for the promising outcomes observed in the phase 2 study, in which highly active RA patients were treated with peresolimab a humanized IgG1 monoclonal antibody stimulating the function of PD-1 (37).

On the other hand, positive correlations between PD-1 expression on particular T cell subsets and levels of inflammatory response were noted in seropositive RA only. This finding can be interpreted by the fact that continuously activated T cells may have a more central role in seropositive versus seronegative RA. Along this line, abatacept which also blocks T cell co-stimulation has better results in seropositive than in seronegative RA patients (38). Notably also, individual systemic inflammation biomarkers demonstrated a positive correlation with frequencies of PD-1^+^ Treg across the three patient groups with active arthritis, as expected, since PD-1 facilitates the proliferation of these particular cells (39).

This study is subject to certain limitations, including not measuring the soluble PD-1, PD-L1, and PD-L2, as well as the PD-L1/2-expressing immune cells. We focused on delineating PD-1 expression in cell populations beyond T cells in fresh whole blood and exploring its potential correlation with disease activity in inflammatory arthropathies, where PD-1 presents as an appealing therapeutic target. Even though we examined the presence of PD-1 on most immune cell subsets, certain cells that are implicated in inflammatory arthritis such as Th9 cells, as well as the cDC1, cDC2, and cDC3, were not addressed. While early untreated RA patients were not included, the exclusive enrollment of bDMARD-naïve patients helped mitigate the confounding effects of treatment on our findings. Moreover, corroborating our single-cell proteomics analysis with single-cell RNA sequencing (scRNA-seq) would strengthen our results. However, to the best of our knowledge, scRNA-seq investigations in fresh whole blood are not publicly available so far, posing a constraint in directly aligning PD-1 gene expression levels with PD-1 protein levels across our target cell subpopulations/subtypes. Nevertheless, the study presents several strengths, including the comparative analysis between PsA and RA, with further stratification of RA into seropositive and seronegative subgroups. Additionally, multiple patients underwent analysis at two distinct time points, facilitating a more nuanced exploration of associations with disease activity.

To conclude, the expression of PD-1 on various circulating immunocytes in chronic autoimmune-mediated arthritis is heterogeneously expressed in all leukocyte subsets and is disproportionately deficient to concomitant high levels of a systemic inflammatory response. Our study offers a deeper insight into PD-1-mediated immune regulation in the context of RA and PsA beyond T cells, shedding light on its intricate relationship with disease activity. These findings contribute to the evolving landscape of immune dysregulation in autoimmune diseases, pointing to the potential therapeutic benefit of pharmacological PD-1 activation to rebalance the autoimmune response and reduce inflammation.

## Data availability statement

The raw data supporting the conclusions of this article will be made available by the authors, without undue reservation.

## Ethics statement

The studies involving humans were approved by Local Ethics and Scientific Committees of the University Hospitals of the National and Kapodistrian University of Athens (No.314/2021). The studies were conducted in accordance with the local legislation and institutional requirements. The participants provided their written informed consent to participate in this study.

## Author contributions

EKV: Conceptualization, Data curation, Formal analysis, Funding acquisition, Investigation, Methodology, Project administration, Resources, Visualization, Writing – original draft, Writing – review & editing. GF: Investigation, Methodology, Resources, Writing – original draft, Writing – review & editing. MK: Formal analysis, Investigation, Visualization, Writing – review & editing. KMV: Formal analysis, Resources, Writing – original draft. MT: Writing – review & editing. TA: Writing – review & editing. PS: Conceptualization, Funding acquisition, Project administration, Supervision, Writing – review & editing.
