# Supplementary material for: Insufficient PD-1 expression during active autoimmune responses: a deep single-cell proteomics analysis in inflammatory arthritis
Source: Front Immunol. 2024 Jun 7;15:1403680. doi: 10.3389/fimmu.2024.1403680 (PMC11190177; doi:10.3389/fimmu.2024.1403680)
Supplement: Supplementary file 1 [file DataSheet_1.pdf]

Supplementary Material

Insufficient PD-1 expression during active autoimmune responses: a deep single-cell proteomics analysis in inflammatory arthritis

Eleni-Kyriaki Vetsika, George E. Fragoulis, Maria Kyriakidi, Kleio-Maria Verrou, Maria G Tektonidou, Themis Alissafi, Petros P. Sfikakis*

*** Correspondence:** Petros P. Sfikakis: psfikakis@med.uoa.gr

1 Supplementary Figures and Tables

1.1 Supplementary Figures

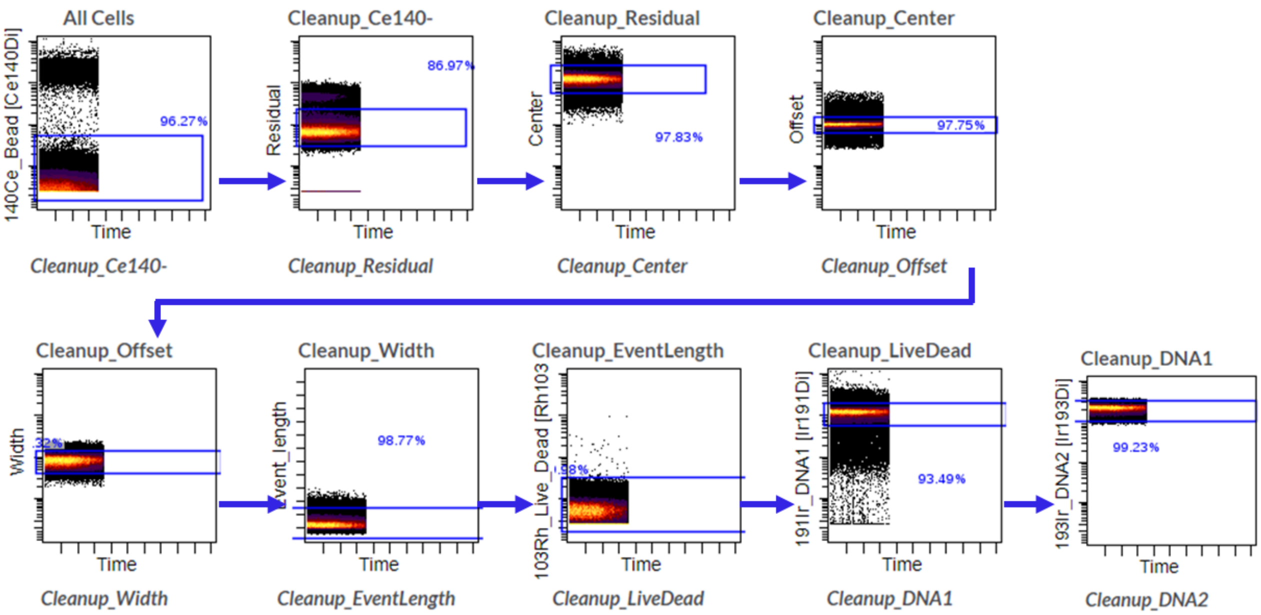

Supplementary Figure 1. Gating strategy followed for data cleanup. Representative dot plots of mass cytometry analysis of data cleanup. The gates for dot plots are presented on the top of each box. Arrows indicate the gating sequence.

A

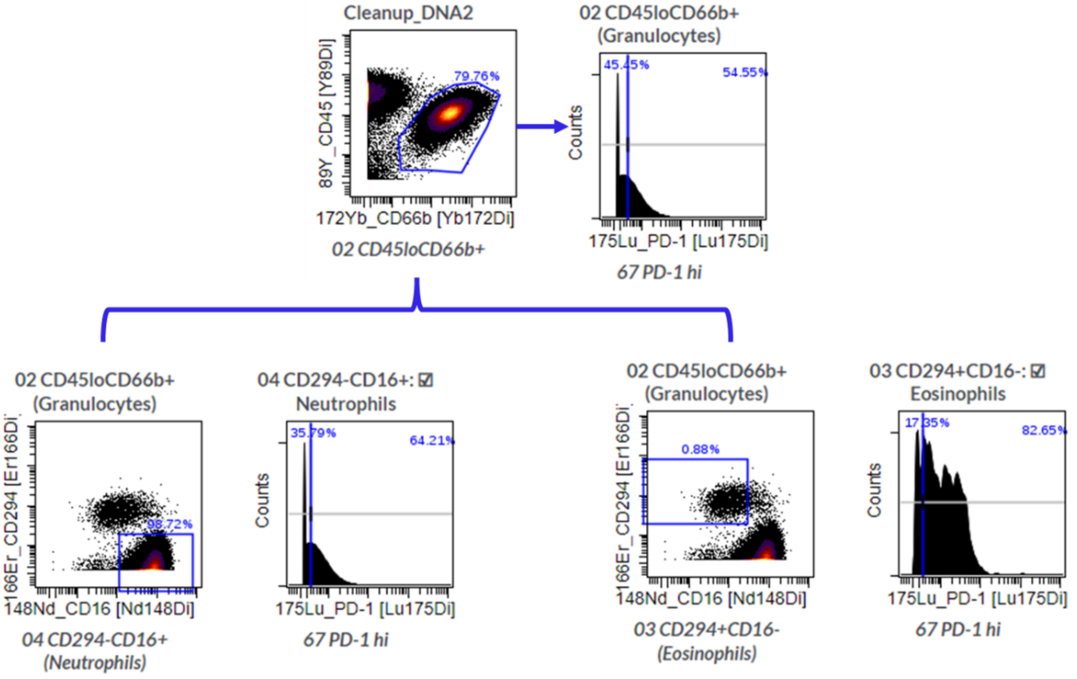

B

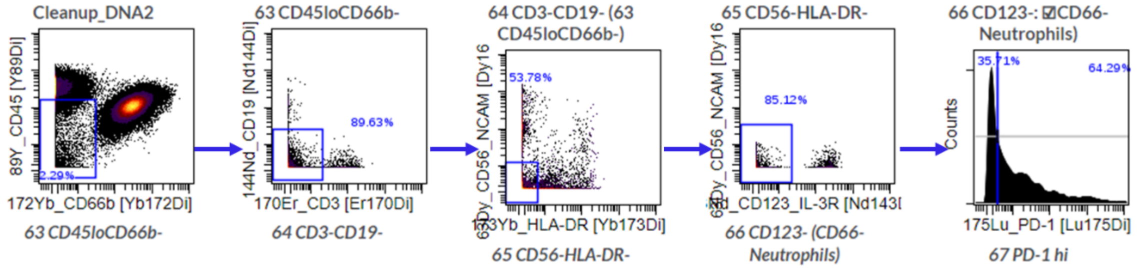

C

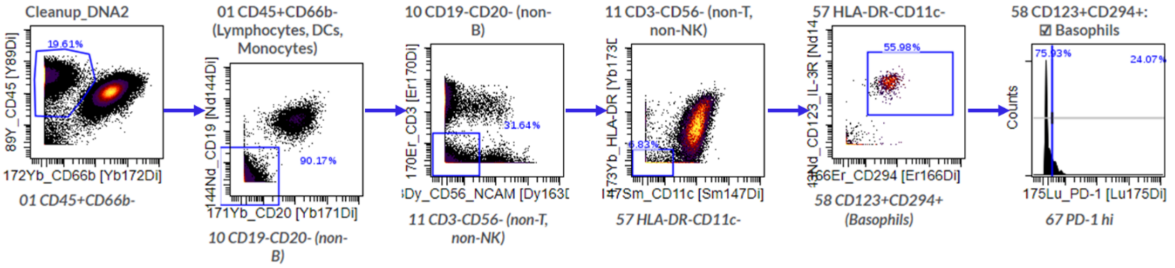

Supplementary Figure 2. Phenotypic analysis of PD-1$^+$ **(A)** Granulocytes, Neutrophils, and Eosinophils, **(B)** CD66b$^-$ Neutrophils and **(C)** Basophils. Representative dot plots, as well as the gating strategy for identification and quantification of granulocytes, neutrophils, eosinophils, CD66b- Neutrophils, and basophils expressing PD-1. Arrows indicate the sequence of gating. The gates for each dot plot are presented on the top of each box.

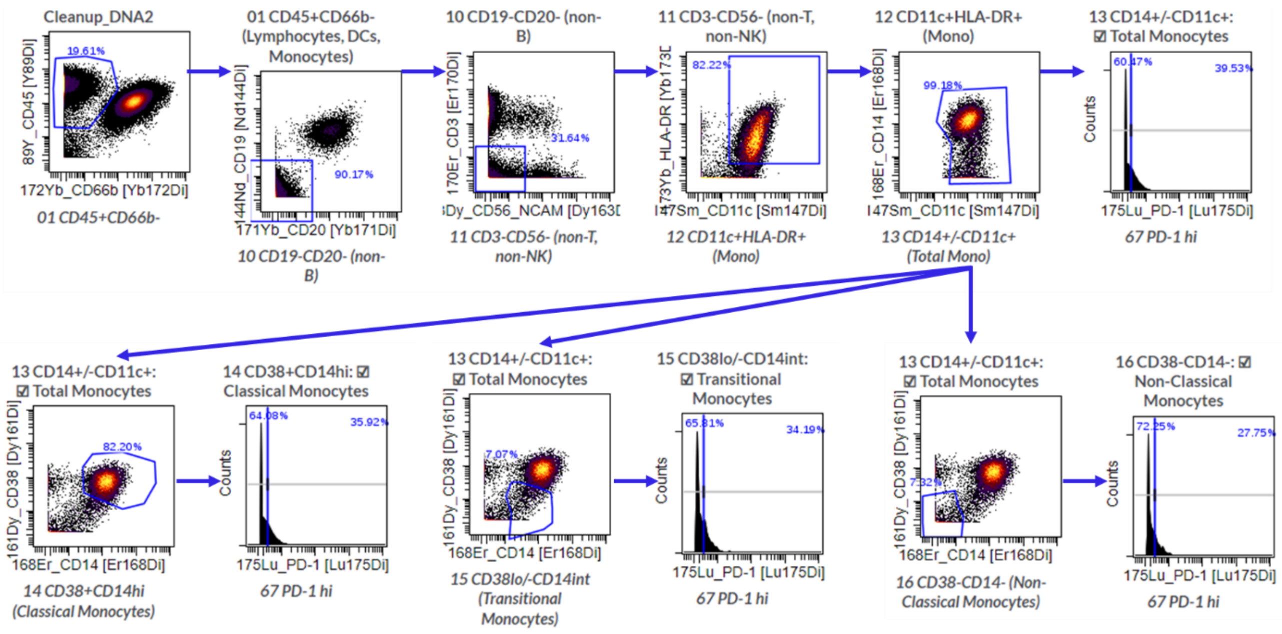

Supplementary Figure 3. Phenotypic analysis of PD-1^{+}Monocytes and their subtypes. Representative dot plots, as well as the gating strategy for identification and quantification of monocytes and their subtypes (classical, transitional, and non-classical) expressing PD-1. Arrows indicate the sequence of gating. The gates for each dot plot are presented on the top of each box.

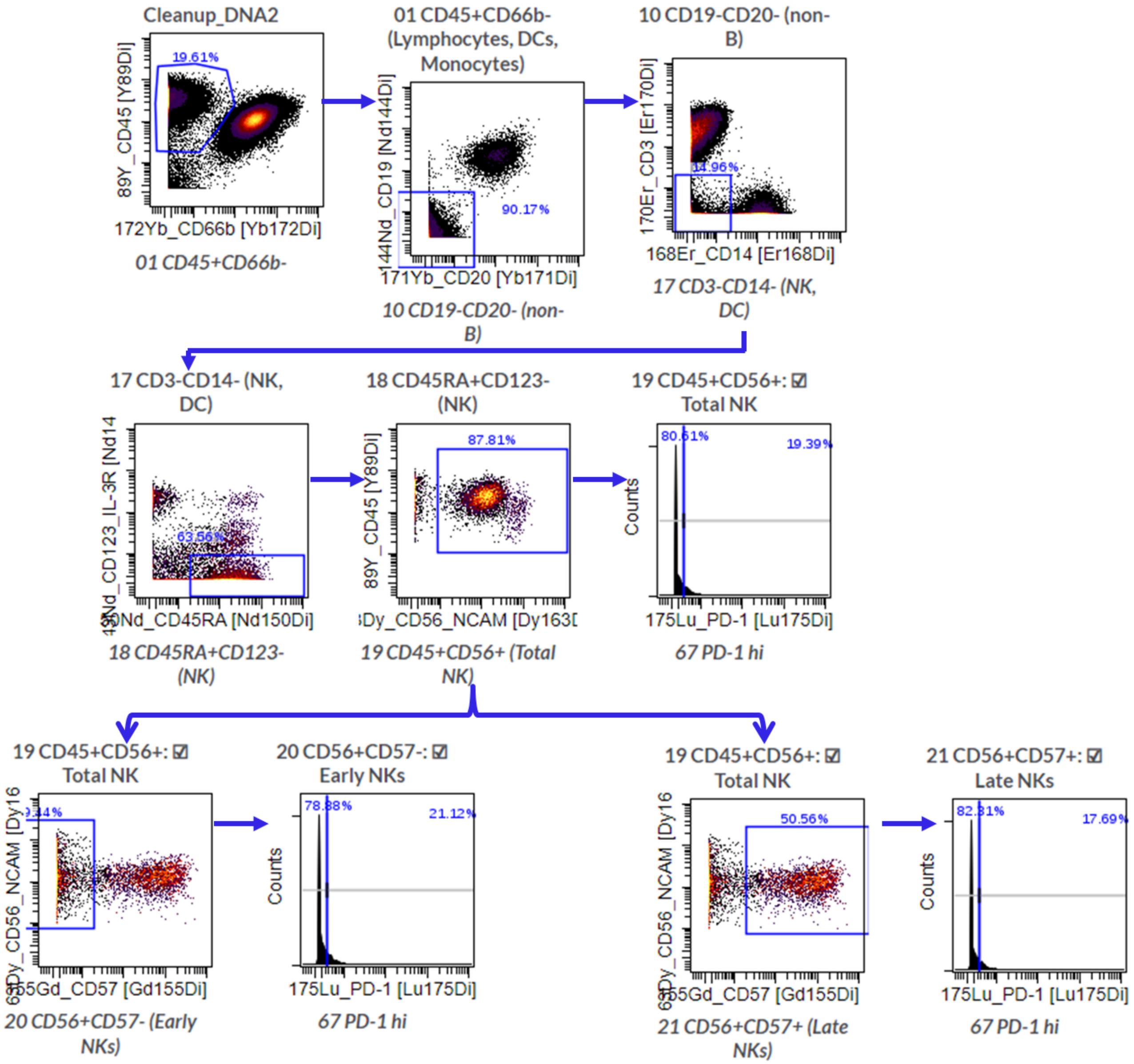

Supplementary Figure 4. Phenotypic analysis of PD-1^{+}NK cells and their subtypes. Representative dot plots, as well as the gating strategy for identification and quantification of NK cells and their subtypes (early and late) expressing PD-1. Arrows indicate the sequence of gating. The gates for each dot plot are presented on the top of each box. (NK: natural killer)

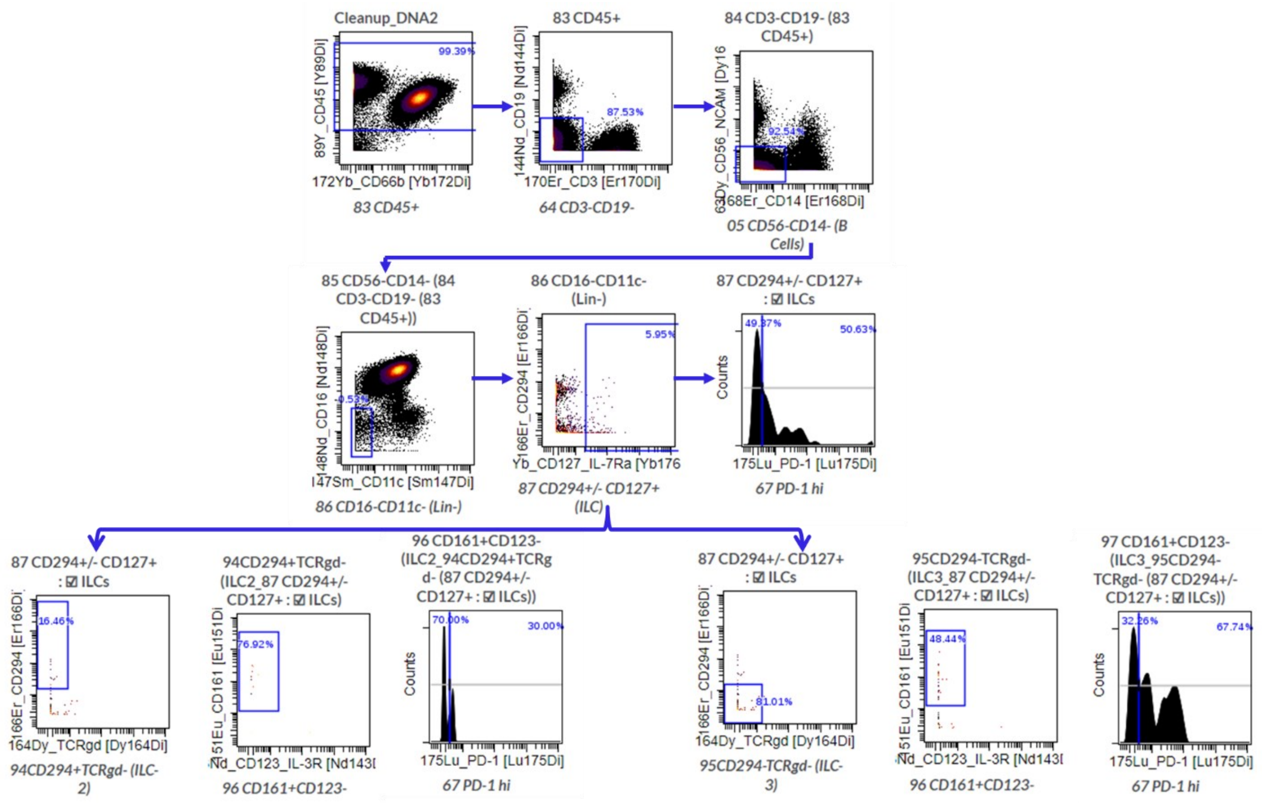

Supplementary Figure 5. Phenotypic analysis of PD-1$^+$ ILC and its subtypes. Representative dot plots, as well as the gating strategy for identification and quantification of ILC cells and their subtypes (ILC2 and ILC3) expressing PD-1. Arrows indicate the sequence of gating. The gates for each dot plot are presented on the top of each box. (ILC: innate lymphoid cells)

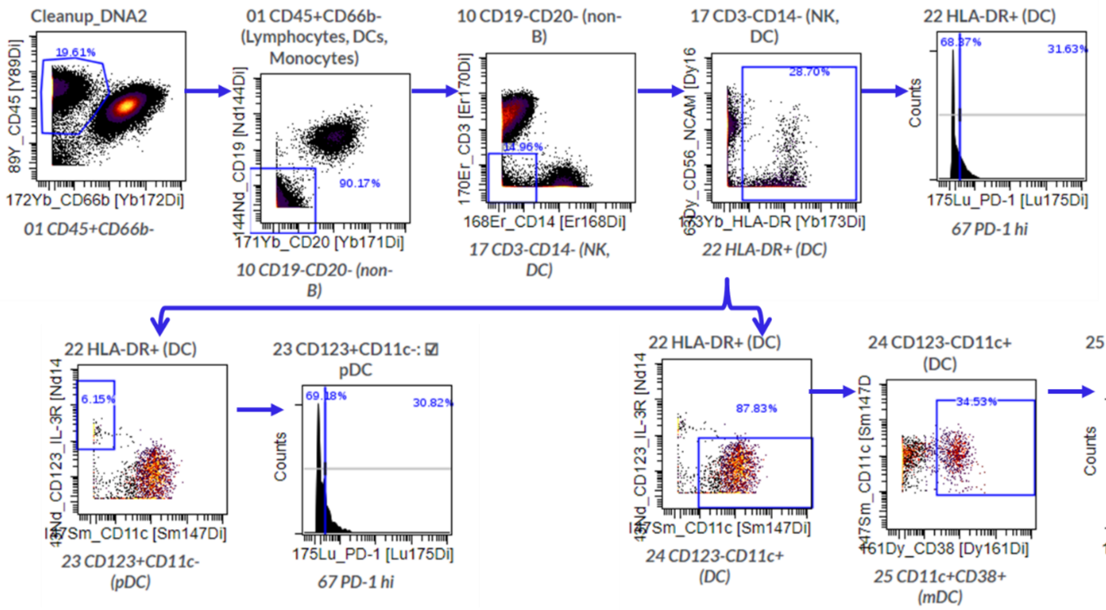

Supplementary Figure 6. Phenotypic analysis of PD-1$^+$ DC and its subtypes. Representative dot plots, as well as the gating strategy for identification and quantification of DC cells and their subtypes (pDC and mDC) expressing PD-1. Arrows indicate the sequence of gating. The gates for each dot plot are presented on the top of each box. (mDC: myeloid dendritic cells; pDC: plasmacytoid dendritic cells)

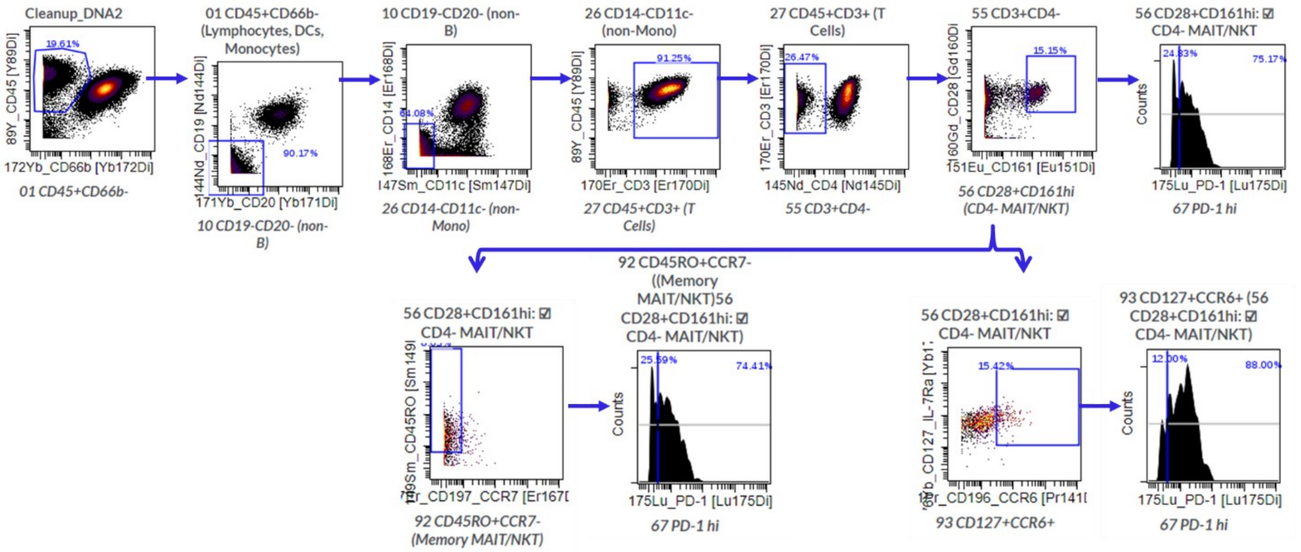

Supplementary Figure 7. Phenotypic analysis of PD-1$^+$MAIT/iNKT and its subtypes. Representative dot plots, as well as the gating strategy for identification and quantification of MAIT/iNKT cells and their subtypes (memory and CD127$^+$CCR6$^+$) expressing PD-1. Arrows indicate the sequence of gating. The gates for each dot plot are presented on the top of each box. (MAIT: mucosal-associated invariant T cells; iNKT: invariant natural killer T cells)

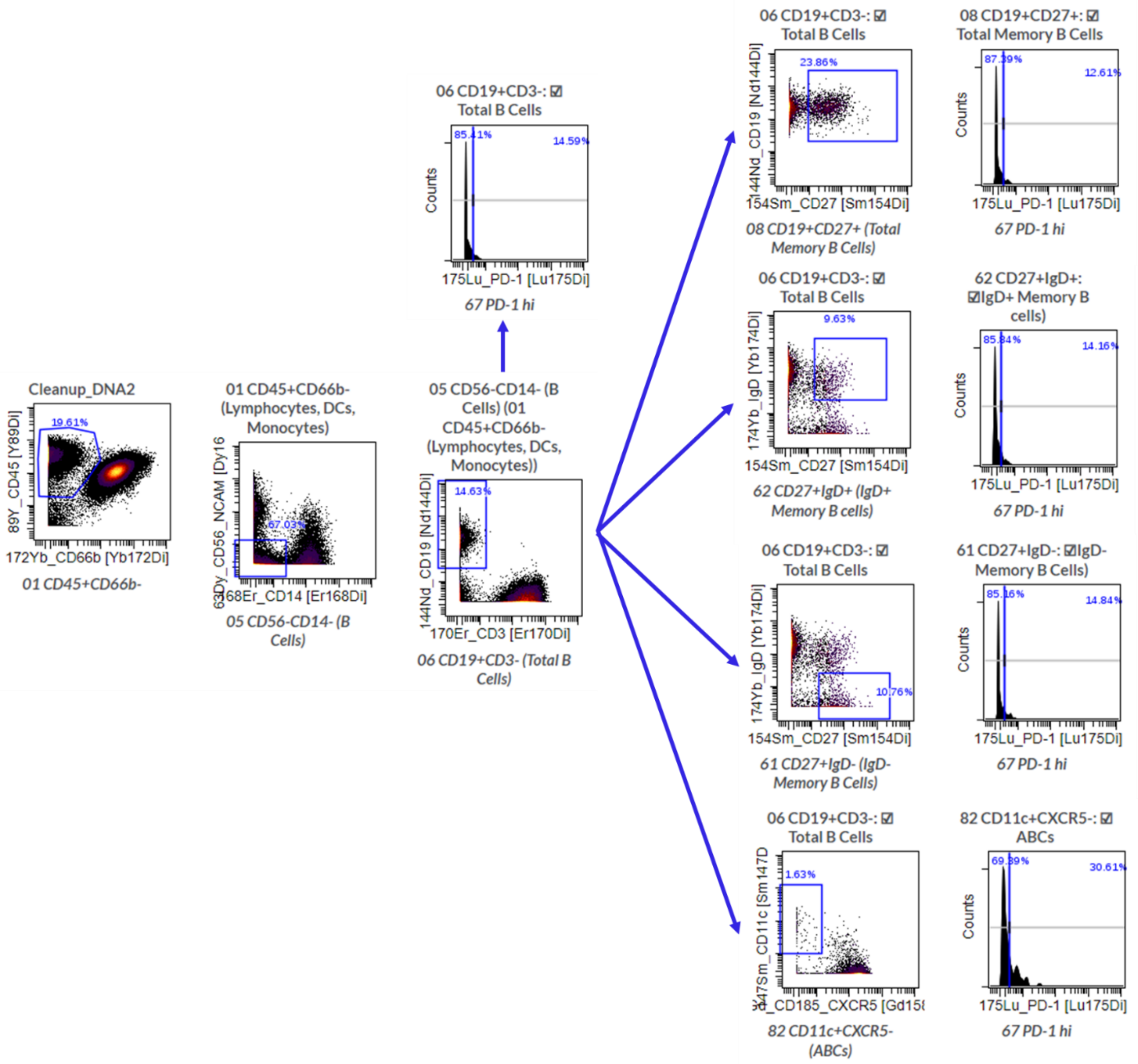

Supplementary Figure 8. Phenotypic analysis of B cells and their subtypes. Representative dot plots, as well as the gating strategy for identification and quantification of B cells and their subtypes (memory, IgD$^+$ and IgD$^-$ Memory B cells and ABC) expressing PD-1. Arrows indicate the sequence of gating. The gates for each dot plot are presented on the top of each box. (ABC: age-associated B cells)

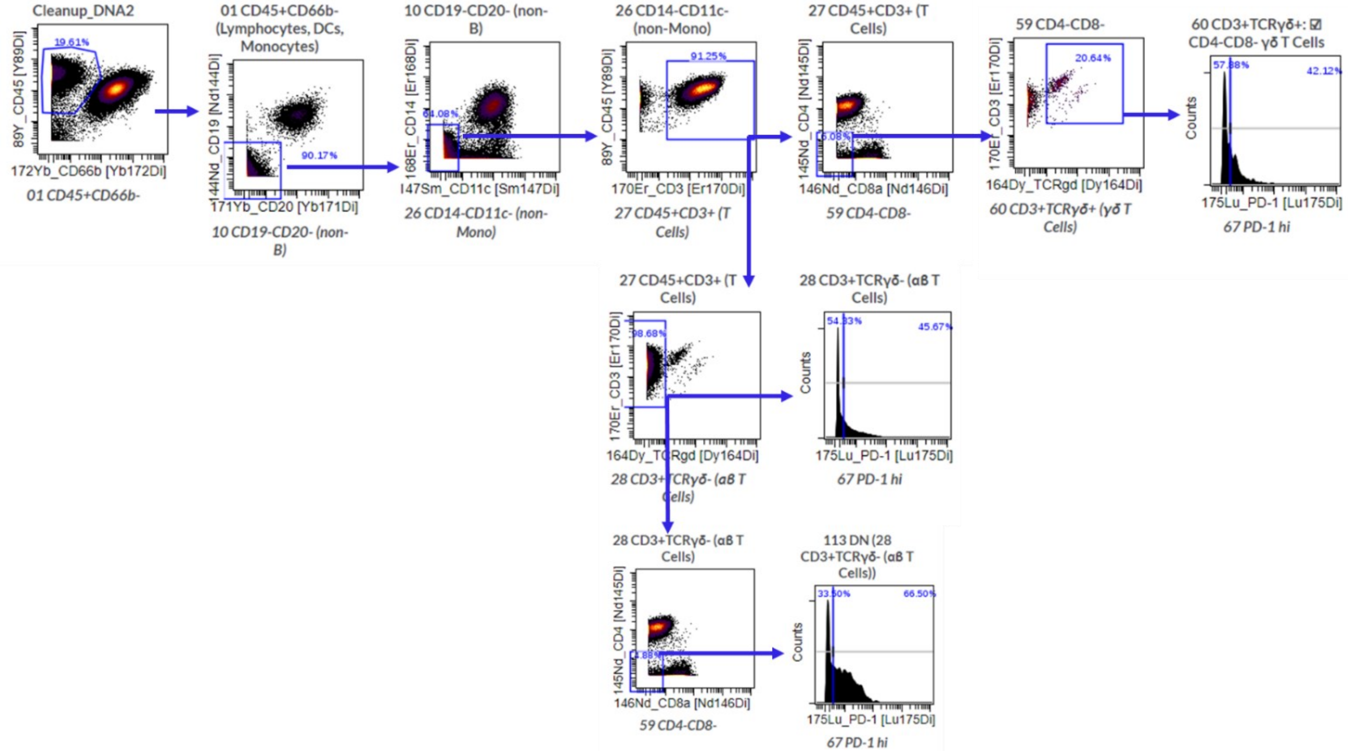

Supplementary Figure 9. Phenotypic analysis of PD-1$^+$ αβT, PD-1$^+$ γδT cells, and PD-1$^+$ DN T subsets. Representative dot plots, as well as the gating strategy for identification and quantification of αβT and γδT cells expressing PD-1. Arrows indicate the sequence of gating. The gates for each dot plot are presented on the top of each box.

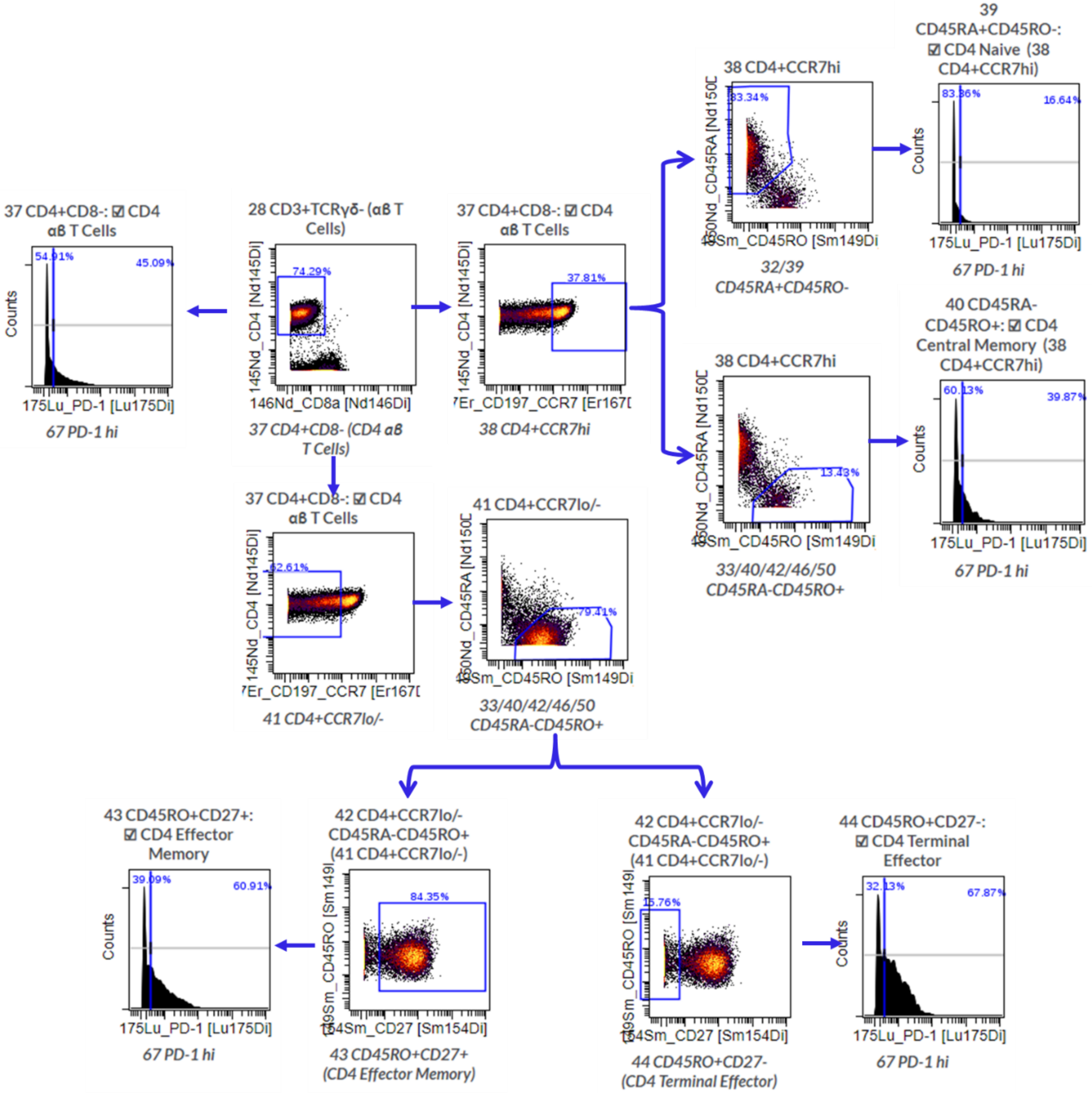

Supplementary Figure 10. Phenotypic analysis of PD-1^{+}CD4^{+} T cells and their subtypes. Representative dot plots, as well as the gating strategy for identification and quantification of CD4^{+} T cells and their subtypes (naïve, CM, EM, and TE) expressing PD-1. Arrows indicate the sequence of gating. The gates for each dot plot are presented on the top of each box. (CM: central memory; EM: effector memory; TE: terminal effector)

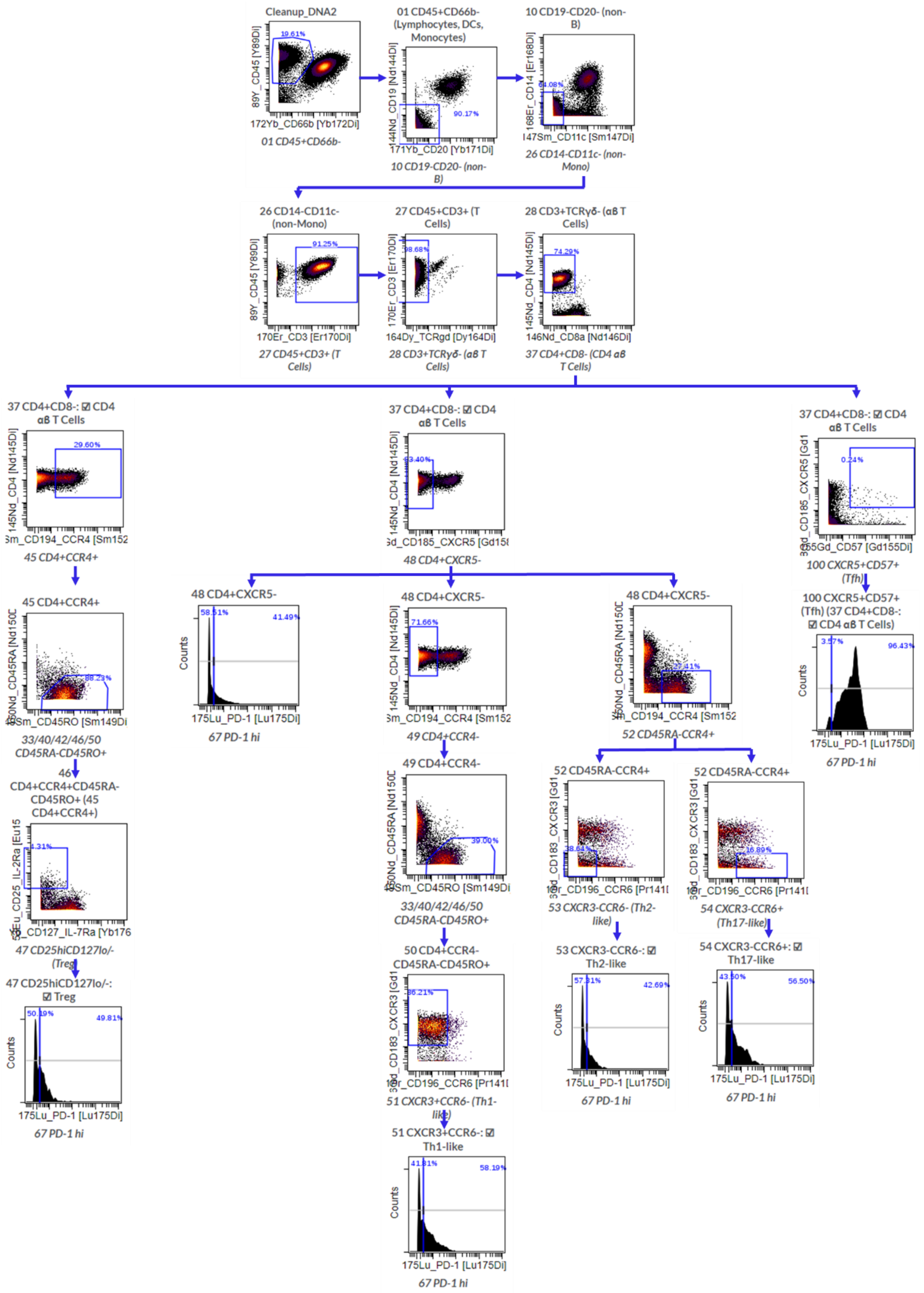

Supplementary Figure 11. Phenotypic analysis of PD-1^{+}CD4^{+} T cell subtypes. Representative dot plots, as well as the gating strategy for identification and quantification of CD4^{+} T cells subtypes (Treg,

Tph, Th1-like, Th2-like, Th17, and Tfh) expressing PD-1. Arrows indicate the sequence of gating. The gates for each dot plot are presented on the top of each box. (Tregs: regulatory T cells; Th: T helper cells; Tfh: T follicular helper, Tph: T peripheral helper)

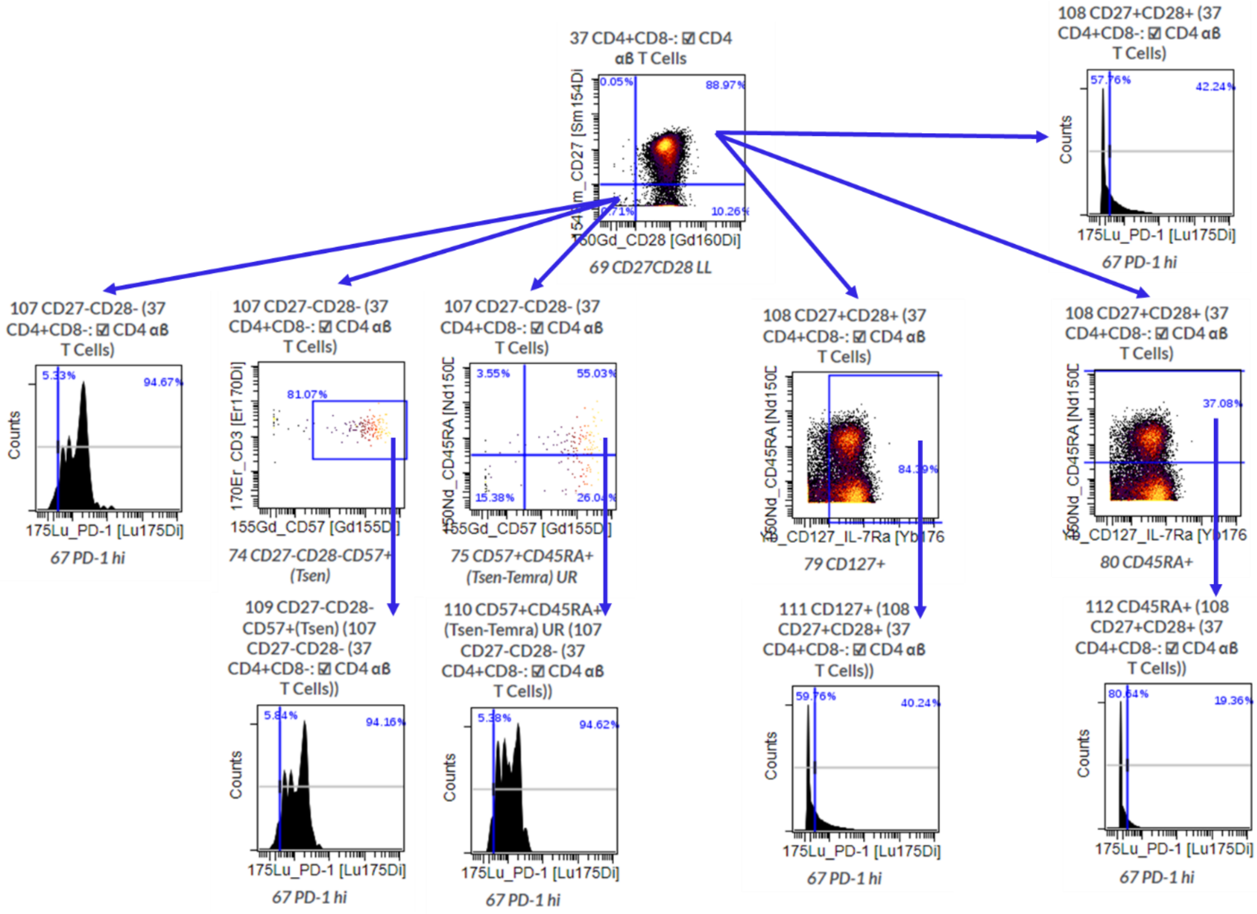

Supplementary Figure 12. Phenotypic analysis of PD-1$^+$CD4$^+$ T cell subtypes. Representative dot plots, as well as the gating strategy for identification and quantification of CD4$^+$ T cells, subtypes (CD27$^-$CD28$^-$, Tsen, Tsen/emra, CD27$^+$CD28$^+$, CD127$^+$CD27$^+$CD28$^+$, and CD45RA$^+$CD27$^+$CD28$^+$) expressing PD-1. Arrows indicate the sequence of gating. The gates for each dot plot are presented on the top of each box. (Tsen: senescent T cells; Temra: effector memory T cells re-expressing CD45RA)

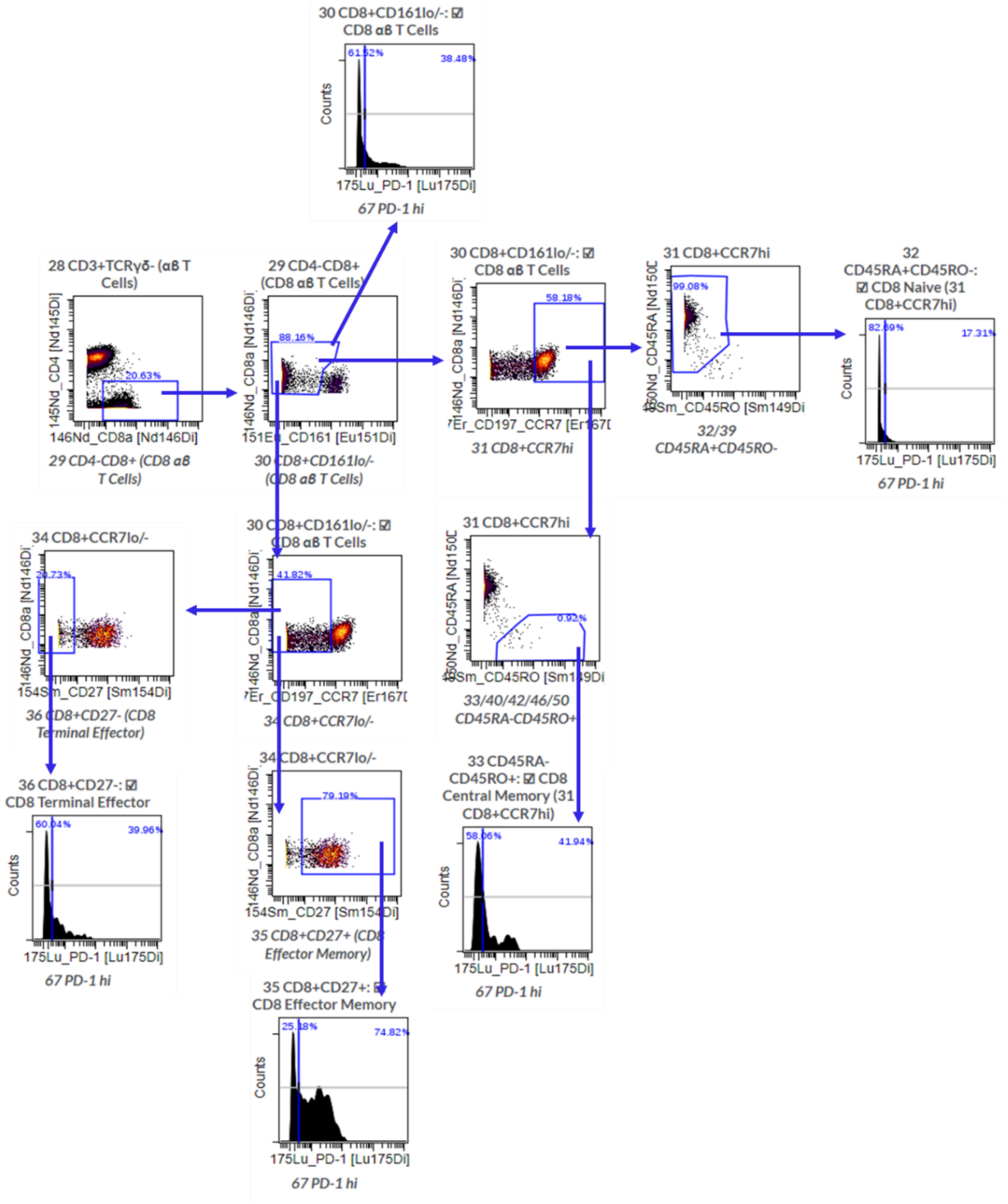

Supplementary Figure 13. Phenotypic analysis of PD-1^{+}CD8^{+} T cells and their subtypes. Representative dot plots, as well as the gating strategy for identification and quantification of CD8^{+} T cells and their subtypes (naïve, CM, EM, and TE) expressing PD-1. Arrows indicate the sequence of gating. The gates for each dot plot are presented on the top of each box. (CM: central memory; EM: effector memory; TE: terminal effector)

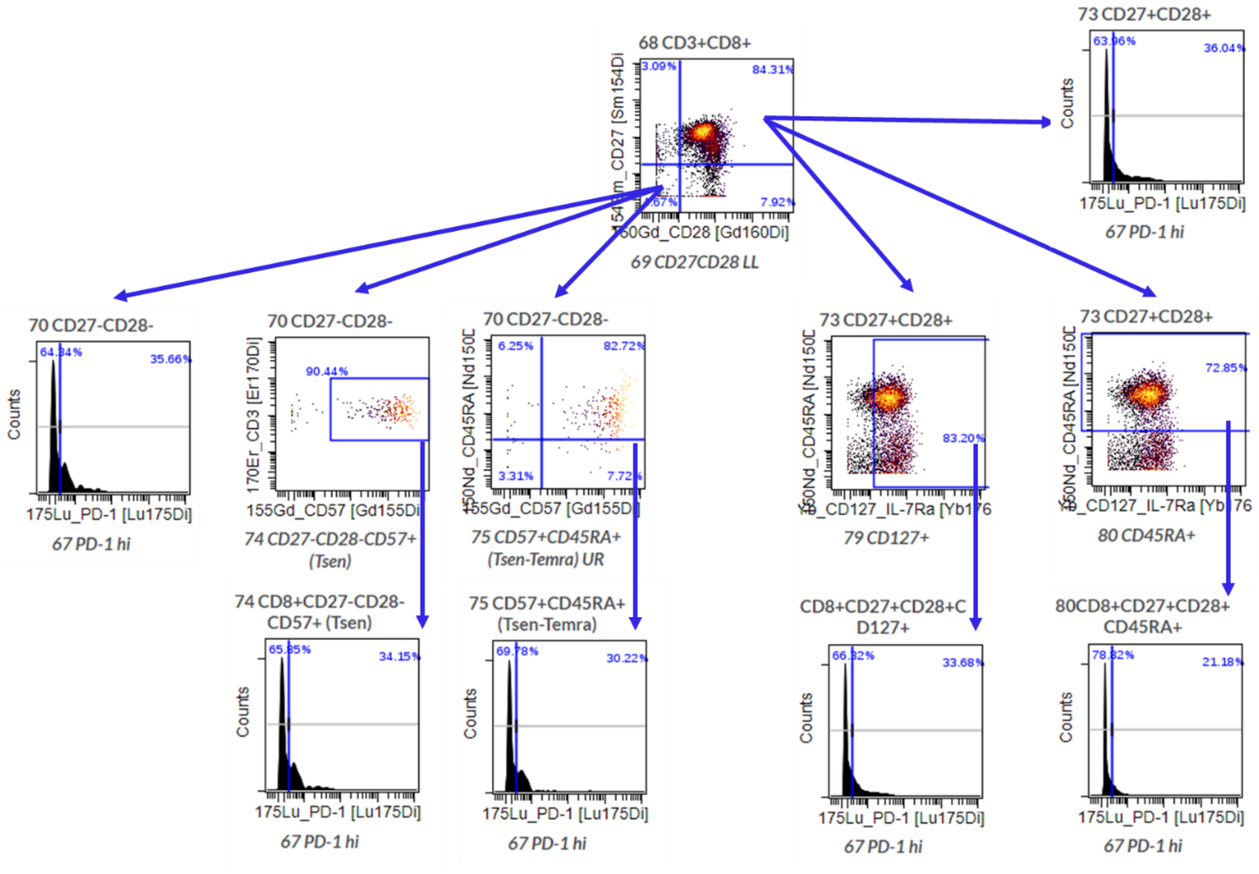

Supplementary Figure 14. Phenotypic analysis of PD-1^{+}CD8^{+} T cell subtypes. Representative dot plots, as well as the gating strategy for identification and quantification of CD8^{+} T cells, subtypes (CD27^{-}CD28^{-}, Tsen, Tsen/emra, CD27^{+}CD28^{+}, CD127^{+}CD27^{+}CD28^{+}, and CD45RA^{+}CD27^{+}CD28^{+}) expressing PD-1. Arrows indicate the sequence of gating. The gates for each dot plot are presented on the top of each box. (Tsen: senescent T cells; Temra: effector memory T cells re-expressing CD45RA)

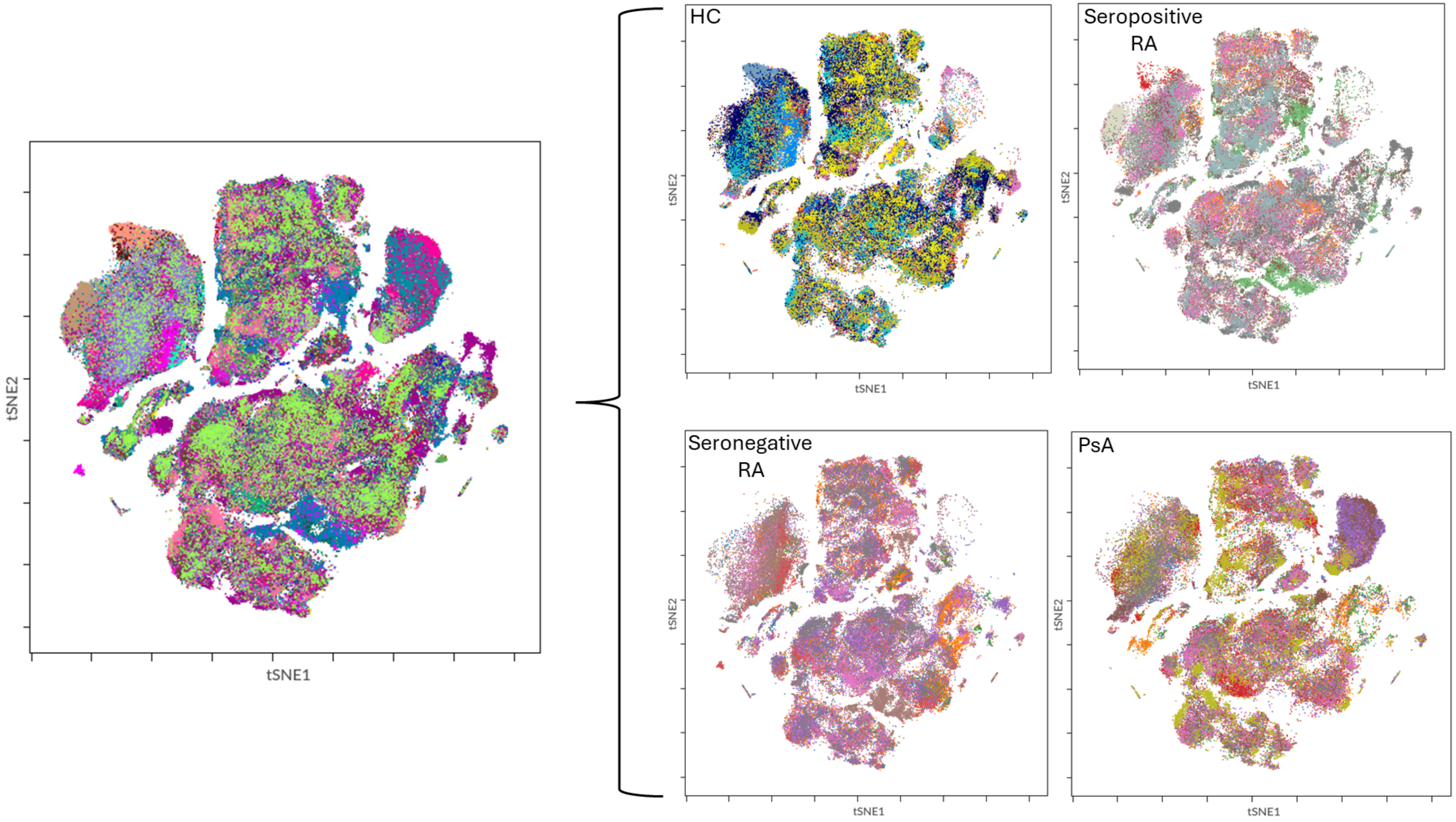

Supplementary Figure 15. viSNE maps of all PD-1-expressing circulating leukocytes. viSNE plots showing 466,624 single PD-1^{+} cells (HC=13; seropositive RA=9; seronegative RA=8; PsA=9). Each dot represents a cell and is colored by the individual subject.

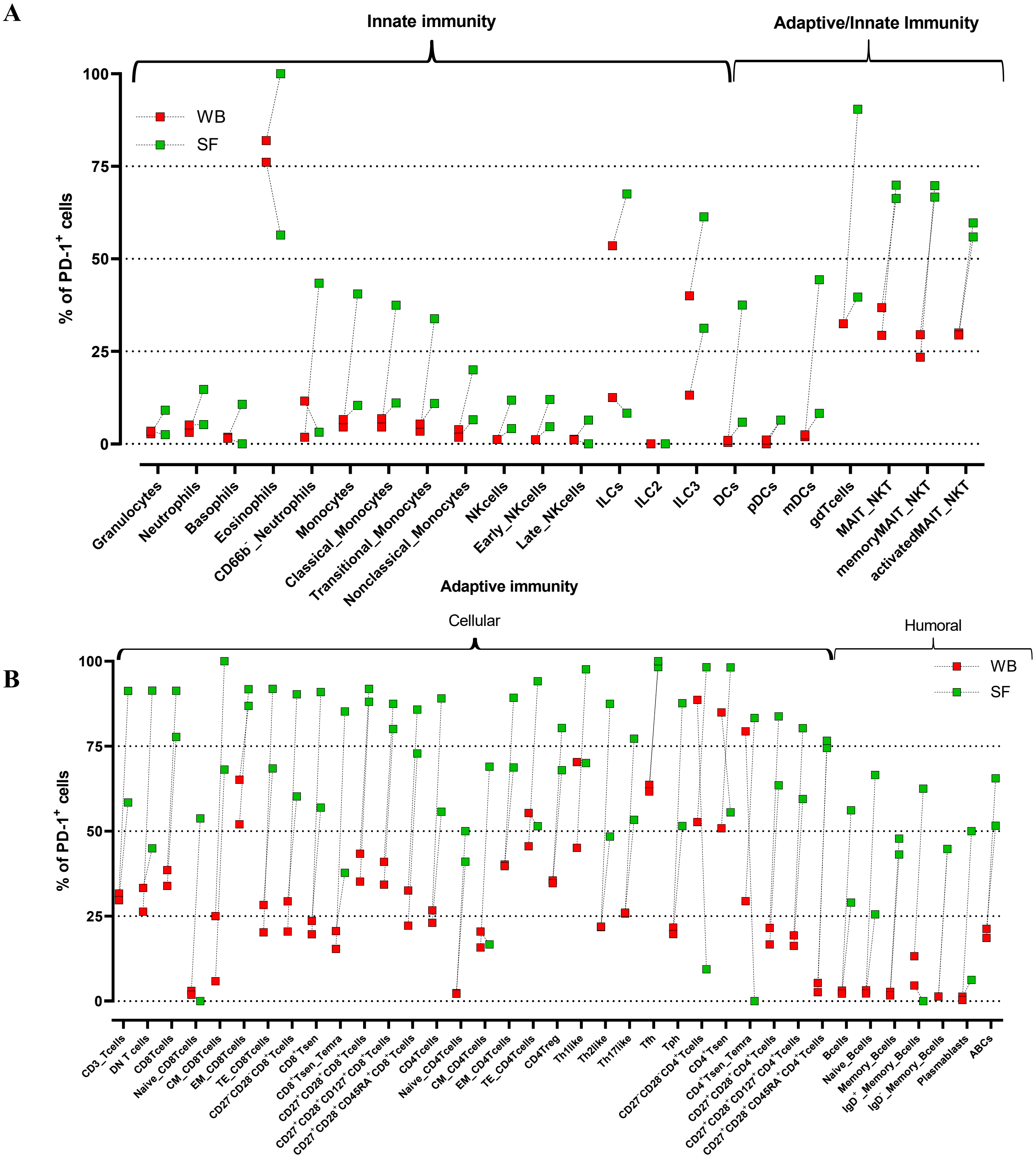

Supplementary Figure 16. CyTOF analysis shows PD-1 expression in all leukocyte subpopulations/subsets in the synovial fluid of 2 RA patients. Plots showing the frequencies of the PD-1^+ leukocytes participating in **(A)** innate immunity and**, (B)** adaptive immunity studied in the peripheral blood and matched synovial fluid from 2 RA patients. (red square: whole blood; green square: synovial fluid)

A

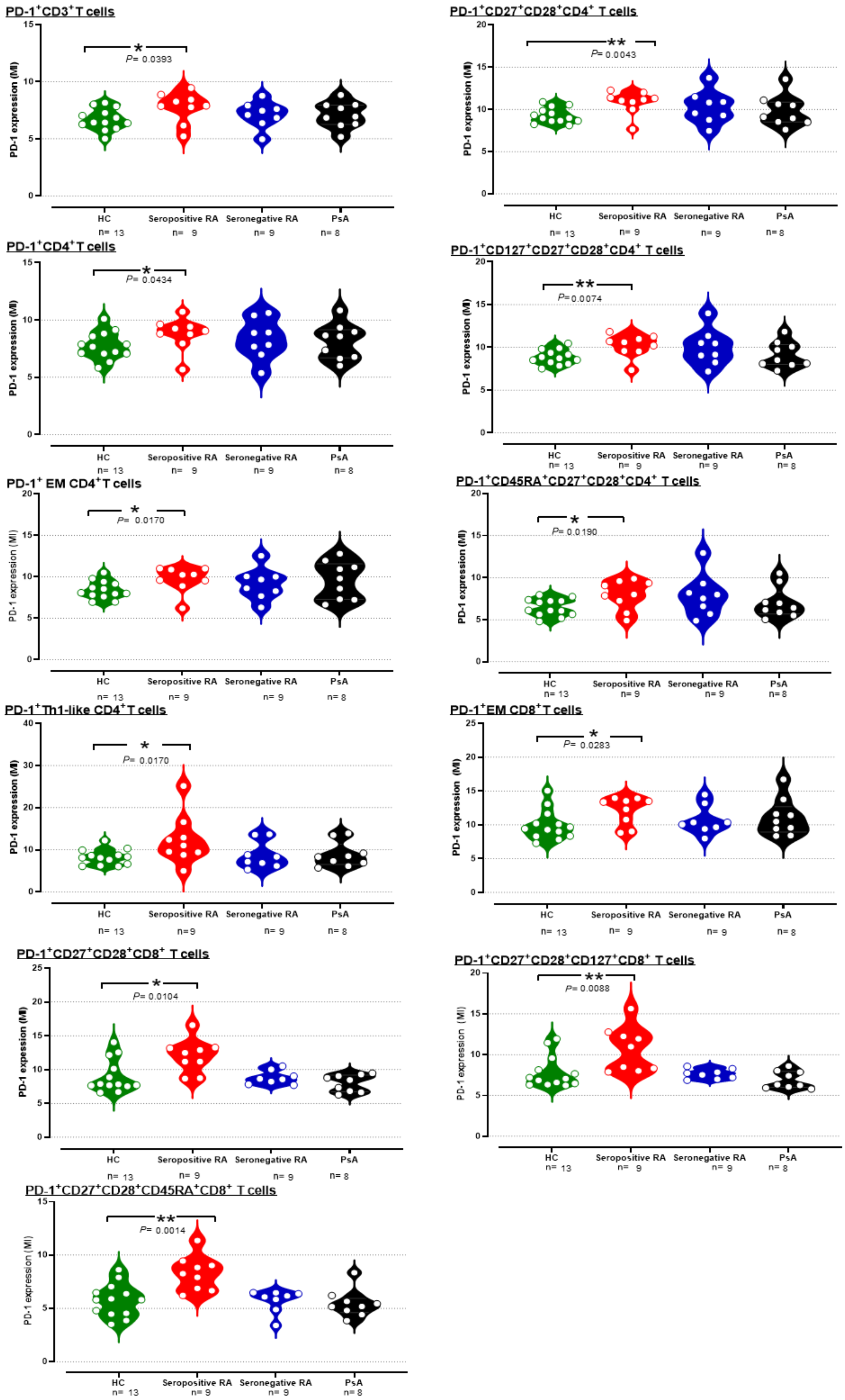
PD-1+CD3+T cells
P= 0.0393
PD-1+CD27+CD28+CD4+ T cells
P= 0.0043
PD-1+CD4+T cells
P= 0.0434
PD-1+CD127+CD27+CD28+CD4+ T cells
P= 0.0074
PD-1+ EM CD4+T cells
P= 0.0170
PD-1+CD45RA+CD27+CD28+CD4+ T cells
P= 0.0190
PD-1+Th1-like CD4+T cells
P= 0.0170
PD-1+EM CD8+T cells
P= 0.0283
PD-1+CD27+CD28+CD8+ T cells
P= 0.0104
PD-1+CD27+CD28+CD127+CD8+ T cells
P= 0.0088
PD-1+CD27+CD28+CD45RA+CD8+ T cells
P= 0.0014
PD-1 expression (MI)
HC
Seropositive RA
Seronegative RA
PsA
n= 13
n= 9
n= 9
n= 8

B

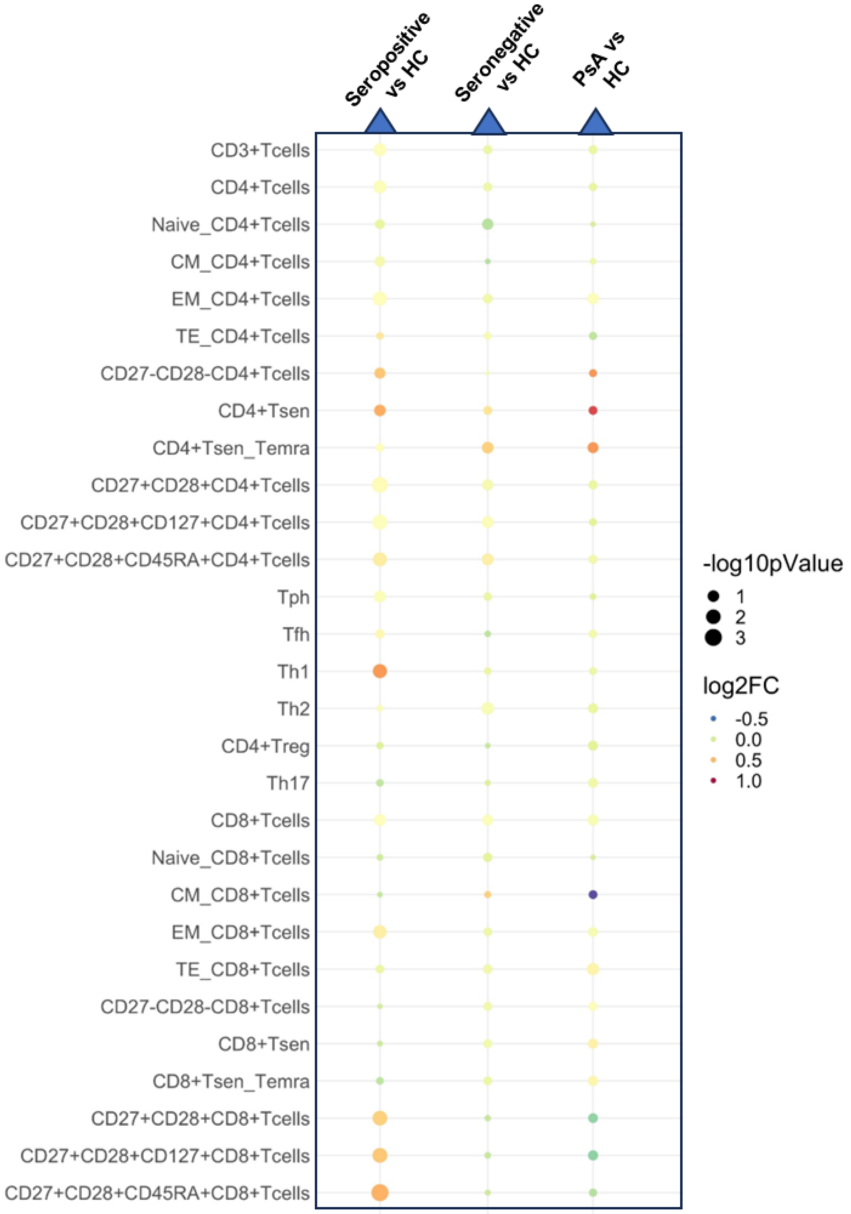

Supplementary Figure 17. (A) PD-1 expression levels (MI) of PD-1$^+$CD3$^+$ T cells, PD-1$^+$CD4$^+$ T cells, EM PD-1$^+$CD4$^+$ T cells, PD-1$^+$Th1-like cells, PD-1$^+$CD27$^+$CD28$^+$CD4$^+$ T cells, PD-1$^+$CD27$^+$CD28$^+$CD127$^+$CD4$^+$ T cells and PD-1$^+$CD27$^+$CD28$^+$CD45RA$^+$CD4$^+$ T cells, EM PD-1$^+$CD8$^+$ T cells, PD-1$^+$CD27$^+$CD28$^+$CD8$^+$ T cells, PD-1$^+$CD27$^+$CD28$^+$CD127$^+$CD8$^+$ T cells and PD-1$^+$CD27$^+$CD28$^+$CD45RA$^+$CD8$^+$ T cells, across the four subgroups. Each point corresponds to an individual patient (green plot= HC, n = 13; red plot = seropositive RA patients, n = 9; blue plot = seronegative RA patients, n = 8; black plot = PsA patients, n = 9). Asterisks indicate statistically significant differences between subgroups based on unpaired t-test or Mann–Whitney U test. (n: number of patients; HC: healthy controls; PsA: psoriatic arthritis; RA: rheumatoid arthritis; CM: central memory; EM: effector memory; TE: terminal effector; Tsen: senescent T cells; Temra: effector memory T cells re-expressing CD45RA; Tregs: regulatory T cells; Th: T helper cells; Tfh: T follicular helper, Tph: T peripheral helper; *, $p \leq 0.05$ and **, $p \leq 0.01$). **(B)** Integrated heatmap/dot-plot showing fold change and p-value between the three patients' cohorts and the HC of the PD-1 expression levels

(MI) on PD-1^{+} T cell subsets of cellular immunity. The size of the dots corresponds to the statistical significance, with bigger dots denoting lower p-values. Coloring corresponds to log2 fold Change, with red denoting a higher abundance in the examined subgroup, while blue denotes a higher abundance in the baseline subgroup.

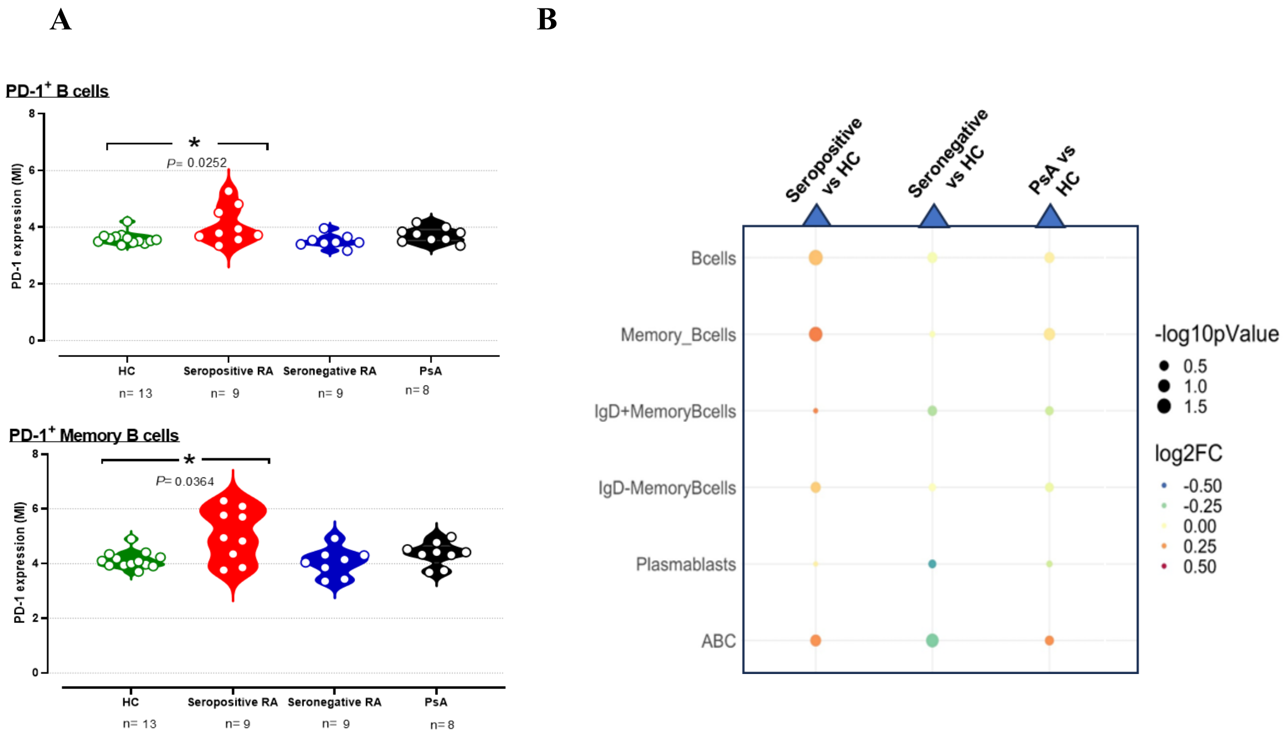

Supplementary Figure 18 (A) PD-1 expression levels (MI) of PD-1^{+} B cells and PD-1^{+} memory B cells, across the four subgroups. Each point corresponds to an individual patient (green plot= HC, n = 13; red plot = seropositive RA patients, n = 9; blue plot = seronegative RA patients, n = 8; black plot = PsA patients, n = 9). Asterisks indicate statistically significant differences between groups based on unpaired t-test or Mann–Whitney U test. (n: number of patients; HC: healthy controls; PsA: psoriatic arthritis; RA: rheumatoid arthritis; ABC: age-associated B cells; *, $p \leq 0.05$). **(B)** Integrated heatmap/dot-plot showing fold change and p-value between the three patients' subgroups and the HC of the PD-1 expression levels (MI) on PD-1^{+}B cell subsets, of humoral immunity. The size of the dots corresponds to the statistical significance, with bigger dots denoting lower p-values. Coloring corresponds to log2 fold Change, with red denoting a higher abundance in the examined subgroup, while blue denotes a higher abundance in the baseline subgroup.

A

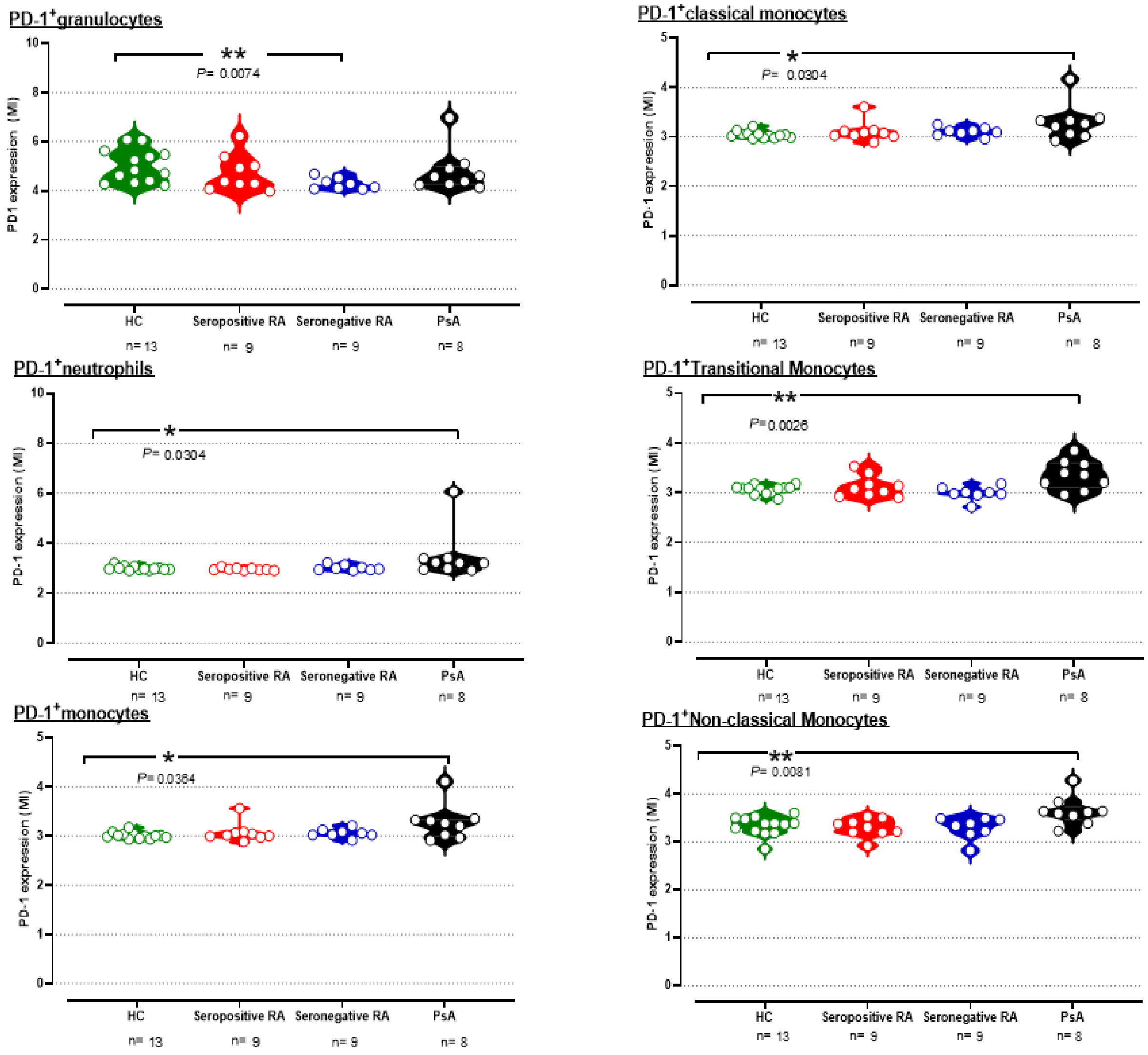
PD-1+granulocytes
PD1 expression (MI)
**
P= 0.0074
HC
Seropositive RA
Seronegative RA
PsA
n= 13
n= 9
n= 9
n= 8
PD-1+classical monocytes
PD-1 expression (MI)
*
P= 0.0304
PD-1+neutrophils
PD-1 expression (MI)
*
P= 0.0304
PD-1+Transitional Monocytes
PD-1 expression (MI)
**
P= 0.0026
PD-1+monocytes
PD-1 expression (MI)
*
P= 0.0364
PD-1+Non-classical Monocytes
PD-1 expression (MI)
**
P= 0.0081

B

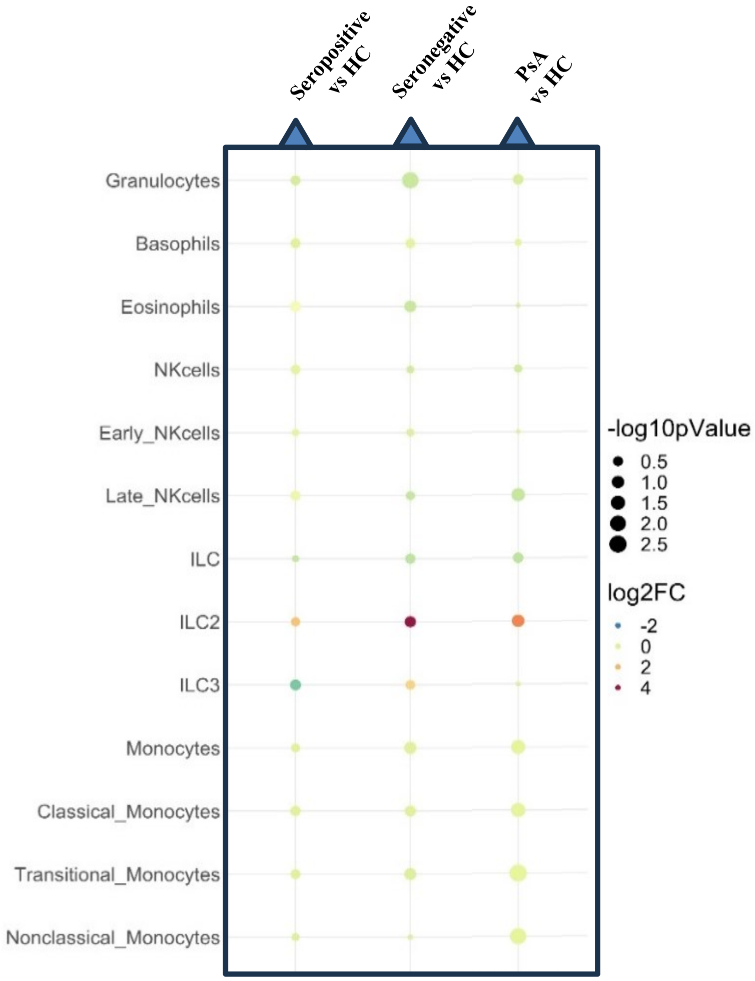

Supplementary Figure 19. **(A)** Violin plots showing the PD-1 expression levels (MI) of the PD-1^+ Granulocytes, PD-1^+ Neutrophils, PD-1^+ Monocytes, and their subsets across the four subgroups. Each point corresponds to an individual patient (green plot = HC, n = 13; red plot = seropositive RA patients, n = 9; blue plot = seronegative RA patients, n = 8; black plot = PsA patients, n = 9;). Asterisks indicate statistically significant differences between groups based on unpaired t-test or Mann–Whitney U test. (n: number of patients; PsA: psoriatic arthritis; RA: rheumatoid arthritis; MI: median intensity; *, $p \leq 0.05$). **(B)** Integrated heatmap/dot-plot showing fold change and p-value between the three patients' cohorts and the HC of the PD-1 expression levels (MI) on PD-1^+ cells of innate immunity. The size of the dots corresponds to the statistical significance, with bigger dots denoting lower p-values. Coloring corresponds to log2 Fold Change, with red denoting a higher abundance in the examined group, while blue denotes a higher abundance in the baseline group.

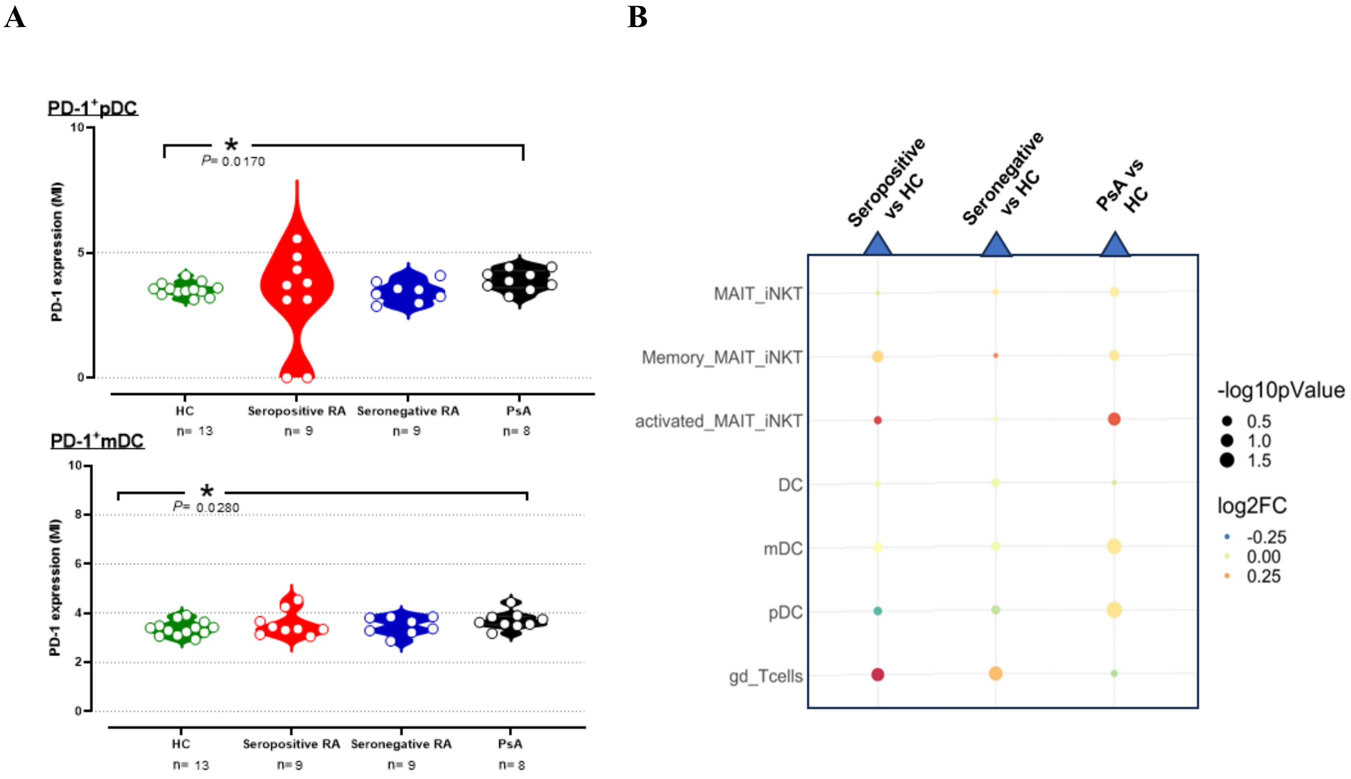

Supplementary Figure 20. (A) Violin plots showing the PD-1 expression levels (MI) of PD-1$^+$pDC and PD-1$^+$mDC across the four cohorts. Each point corresponds to an individual patient (green plot = HC, n = 13; red plot = seropositive RA patients, n = 9; blue plot = seronegative RA patients, n = 8; black plot = PsA patients, n = 9). Asterisks indicate statistically significant differences between subgroups based on unpaired t-test or Mann–Whitney U test. (n: number of patients; PsA: psoriatic arthritis; RA: rheumatoid arthritis; DC: dendritic cells; pDC: plasmacytoid DC; mDC: myeloid DC; MI: median intensity; *, $p \leq 0.05$). **(B)** Integrated heatmap/dot-plot showing fold change and p-value between the three patients' subgroups and the HC of the PD-1 expression levels (MI) on PD-1$^+$ cells of innate/adaptive immunity. The size of the dots corresponds to the statistical significance, with bigger dots denoting lower p-values. Coloring corresponds to log2 fold Change, with red denoting a higher abundance in the examined group, while blue denotes a higher abundance in the baseline group.

A. Innate Immunity

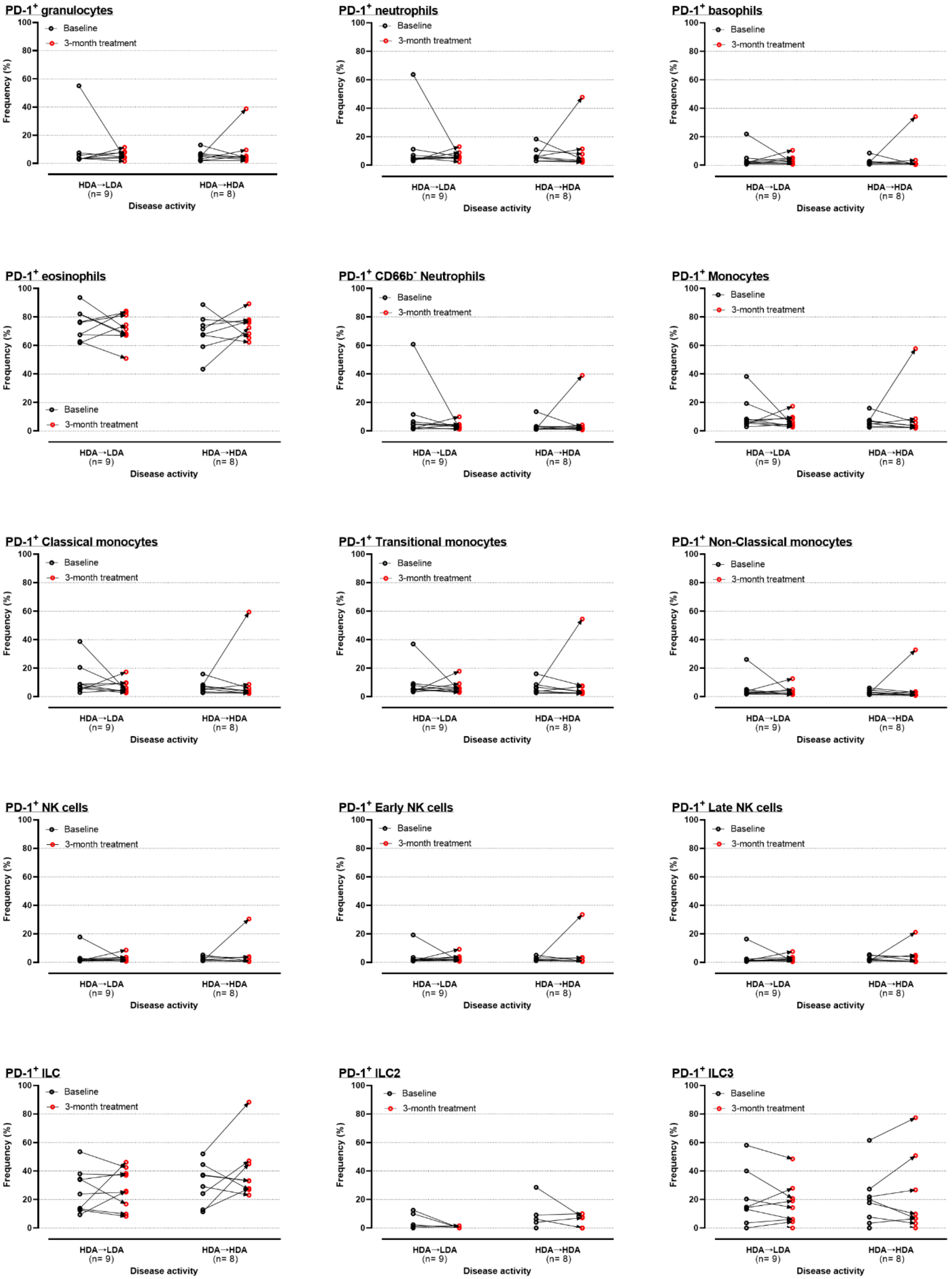

B. Innate and adaptive immunity

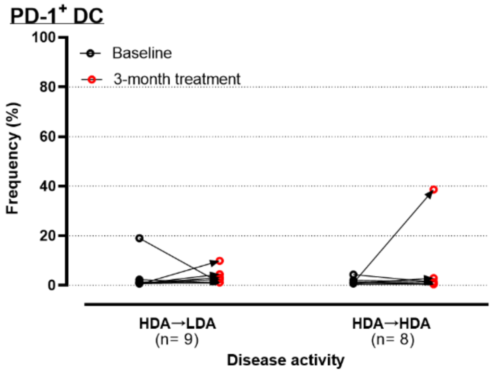

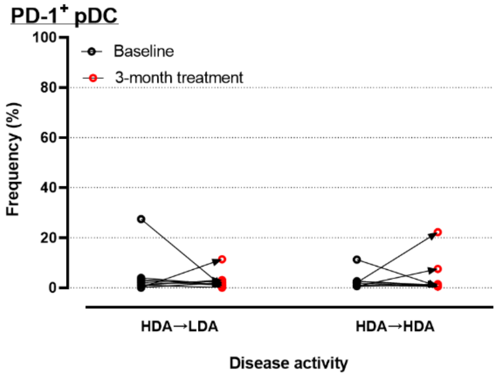

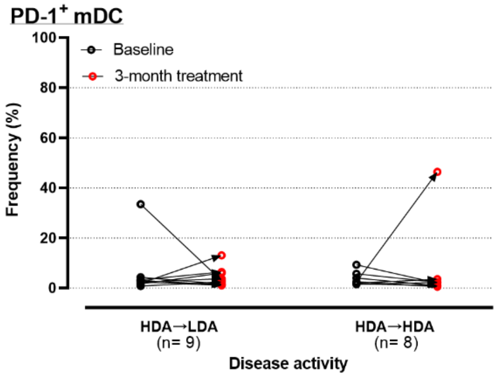

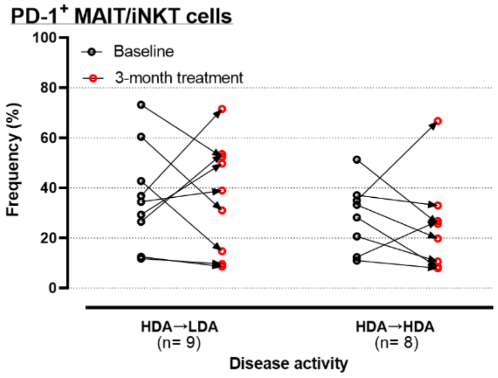

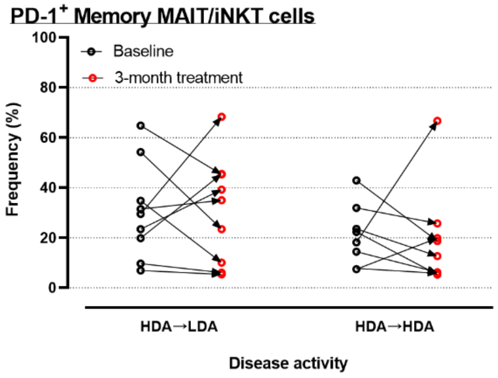

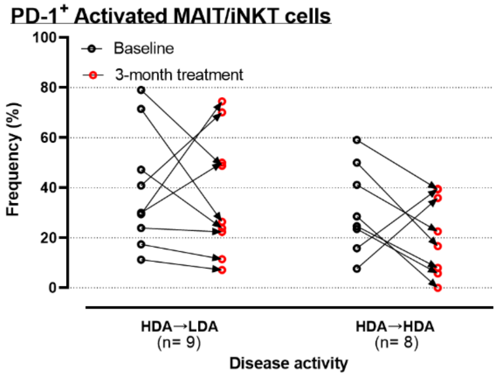

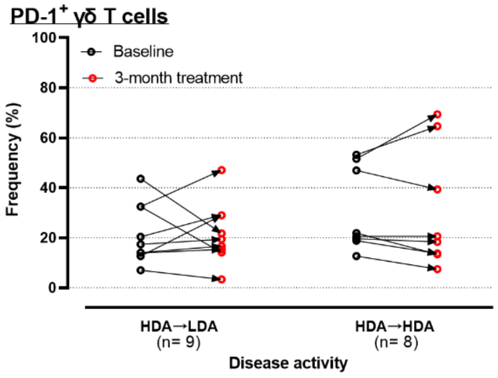

C. Adaptive immunity

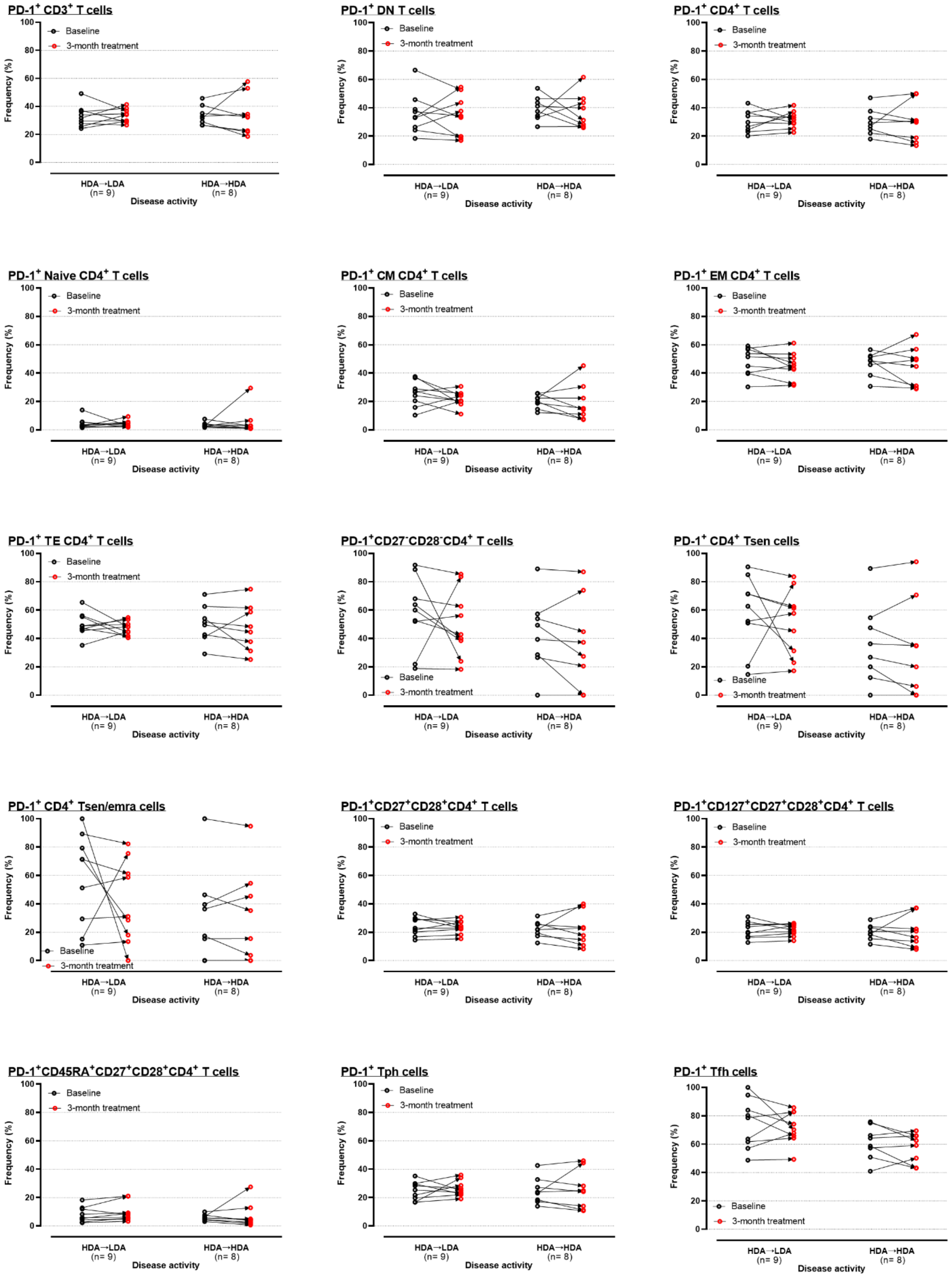

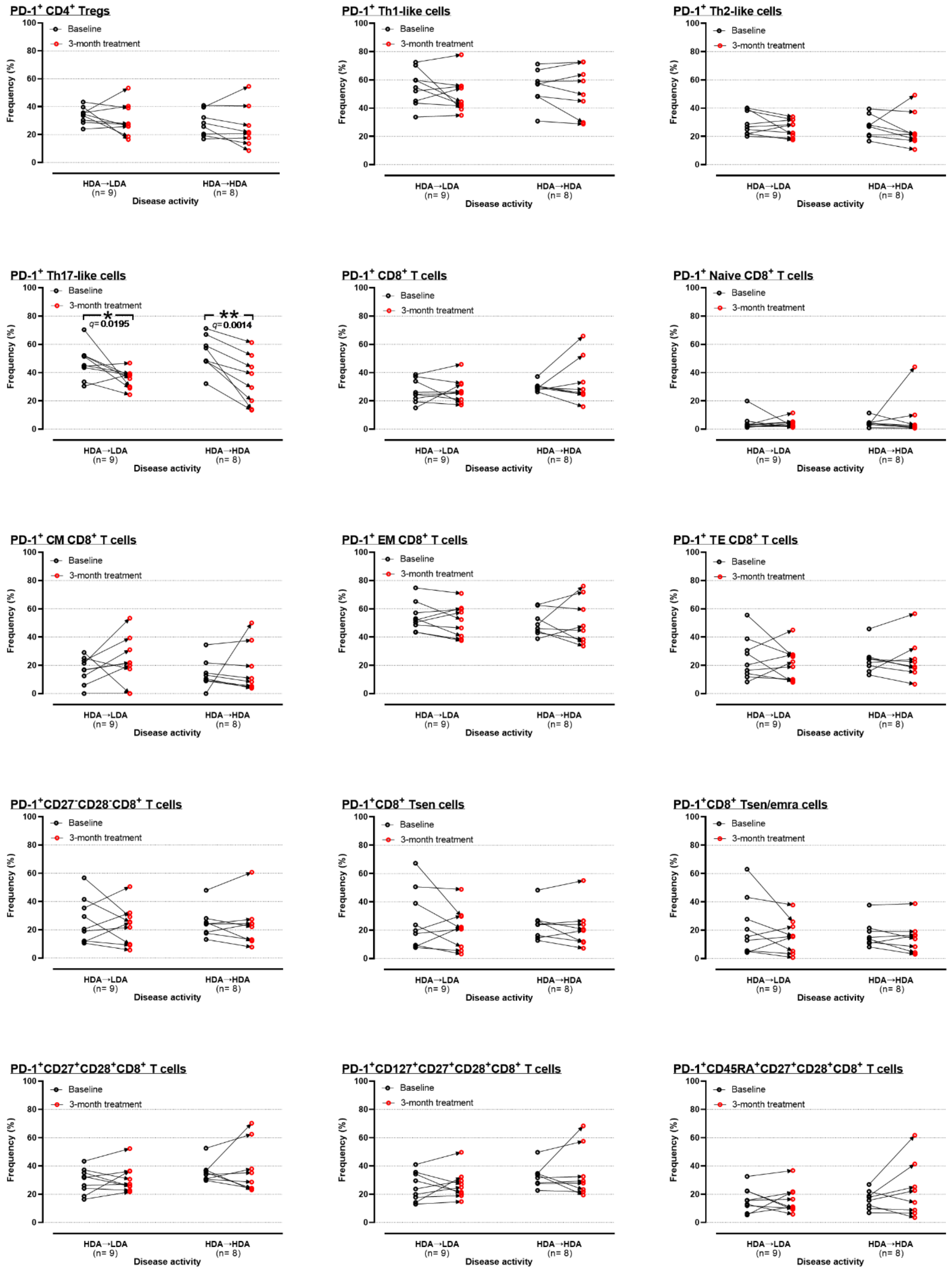
PD-1$^+$ CD4$^+$ Tregs
PD-1$^+$ Th1-like cells
PD-1$^+$ Th2-like cells
PD-1$^+$ Th17-like cells
PD-1$^+$ CD8$^+$ T cells
PD-1$^+$ Naive CD8$^+$ T cells
PD-1$^+$ CM CD8$^+$ T cells
PD-1$^+$ EM CD8$^+$ T cells
PD-1$^+$ TE CD8$^+$ T cells
PD-1$^+$CD27$^-$CD28$^-$CD8$^+$ T cells
PD-1$^+$CD8$^+$ Tsen cells
PD-1$^+$CD8$^+$ Tsen/emra cells
PD-1$^+$CD27$^+$CD28$^+$CD8$^+$ T cells
PD-1$^+$CD127$^+$CD27$^+$CD28$^+$CD8$^+$ T cells
PD-1$^+$CD45RA$^+$CD27$^+$CD28$^+$CD8$^+$ T cells
Baseline
3-month treatment
Frequency (%)
HDA→LDA (n= 9)
HDA→HDA (n= 8)
Disease activity
*
q=0.0195
**
q=0.0014

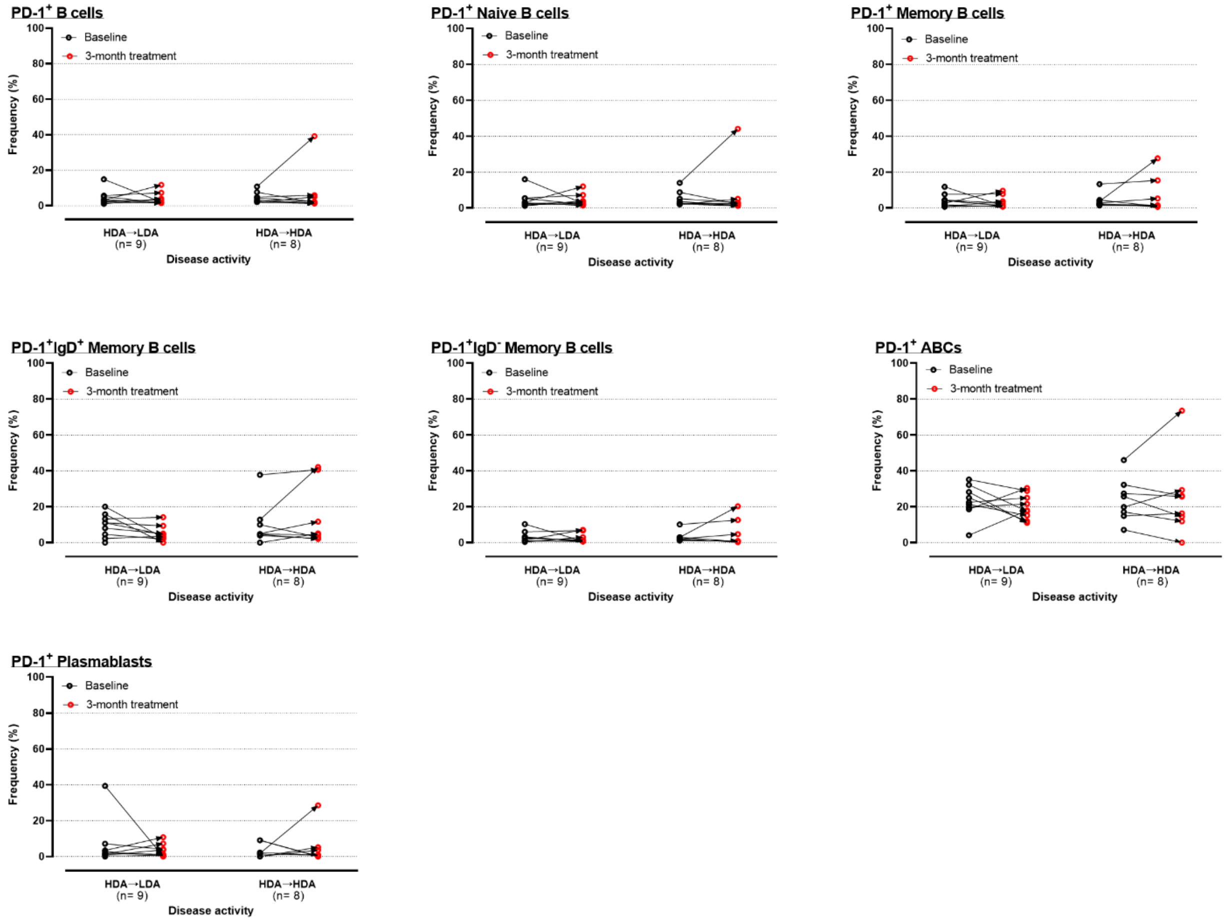

Supplementary Figure 21. Changes in PD-1$^+$ immune cell subpopulations/subsets after three months of treatments according to disease activity. The frequencies of PD-1$^+$ immune cells of **(A)** innate immunity, **(B)** innate and adaptive immunity and **(C)** adaptive immunity, in patients with IA at baseline and after 3 months of treatments, according to their disease activity after 3 months. Each circle corresponds to an individual patient (open black circles = baseline; open red circles = after 3 months). Groups were compared by performing two-way ANOVA corrected for multiple comparisons by false discovery rate (FDR) using the two-stage linear step-up procedure of Benjamini, Krieger and Yekutieli. (n: number of patients; IA: Inflammatory arthritis; NK: natural killer cells; DN: Double-Negative; CM: central memory; EM: effector memory; TE: terminal effector; Tsen: senescent T cells; Temra: effector memory T cells re-expressing CD45RA; Tregs: regulatory T cells; Th: T helper cells; Tfh: T follicular helper, Tph: T peripheral helper; ABC: age-associated B cells; DC: dendritic cells; mDC: myeloid dendritic cells; pDC: plasmacytoid dendritic cells; MAIT: mucosal-associated invariant T cells; iNKT: invariant natural killer T cells; ILCs: innate lymphoid cells; HDA: high disease activity (active disease); LDA: low disease activity (inactive disease); *, $q \leq 0.05$; **, $q \leq 0.01$)

A. Innate Immunity

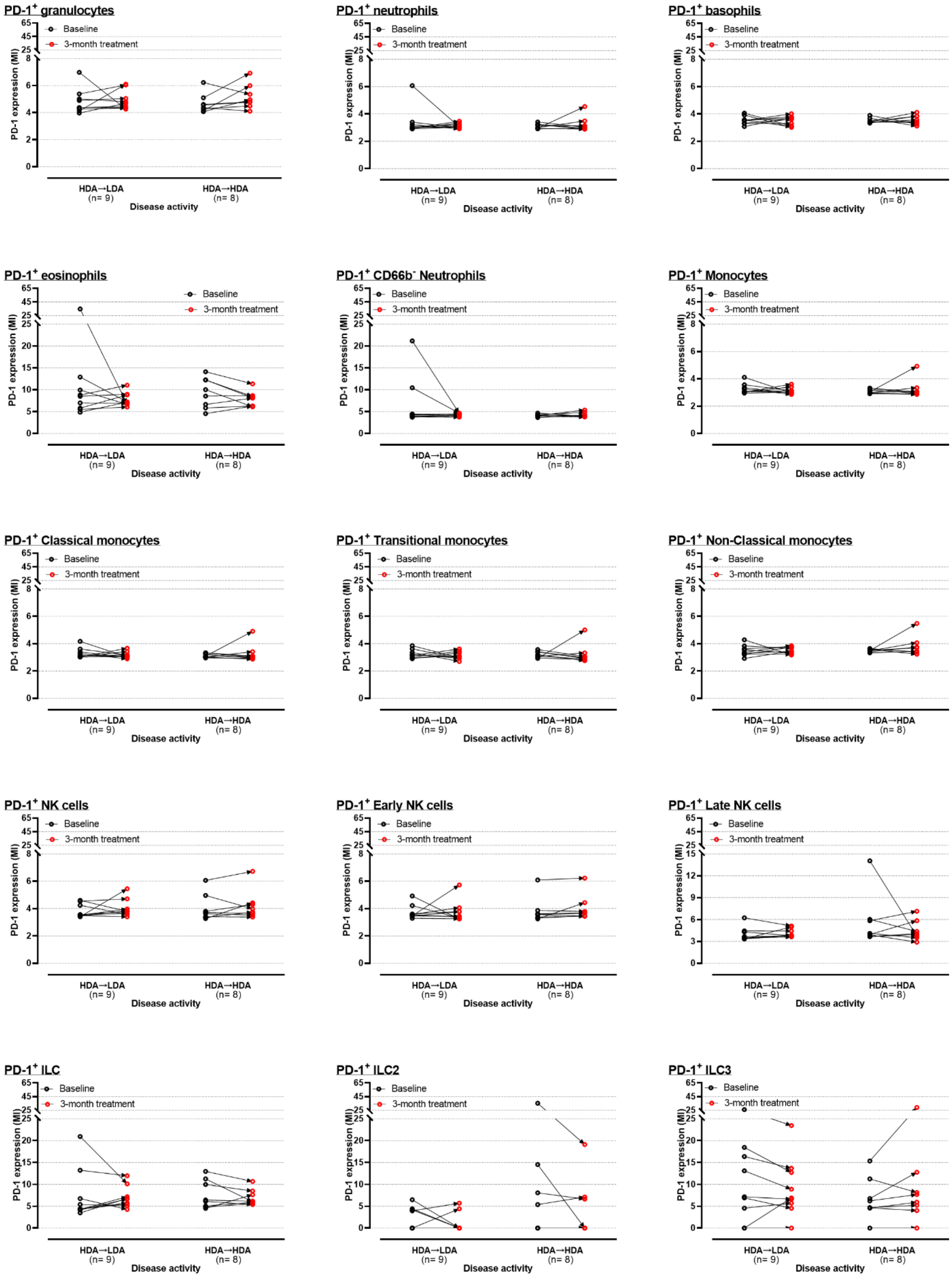

B. Innate and Adaptive Immunity

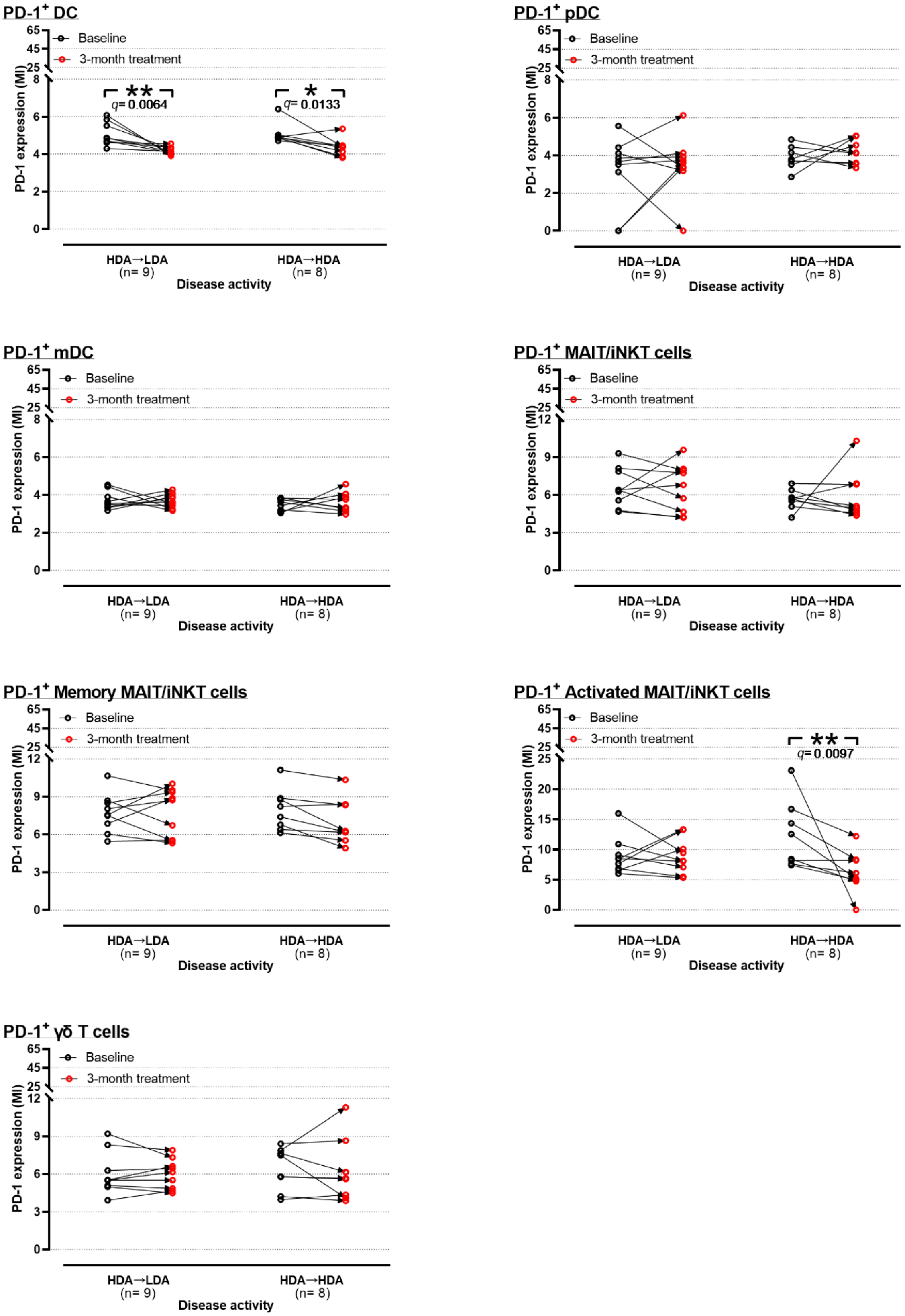

C. Adaptive Immunity

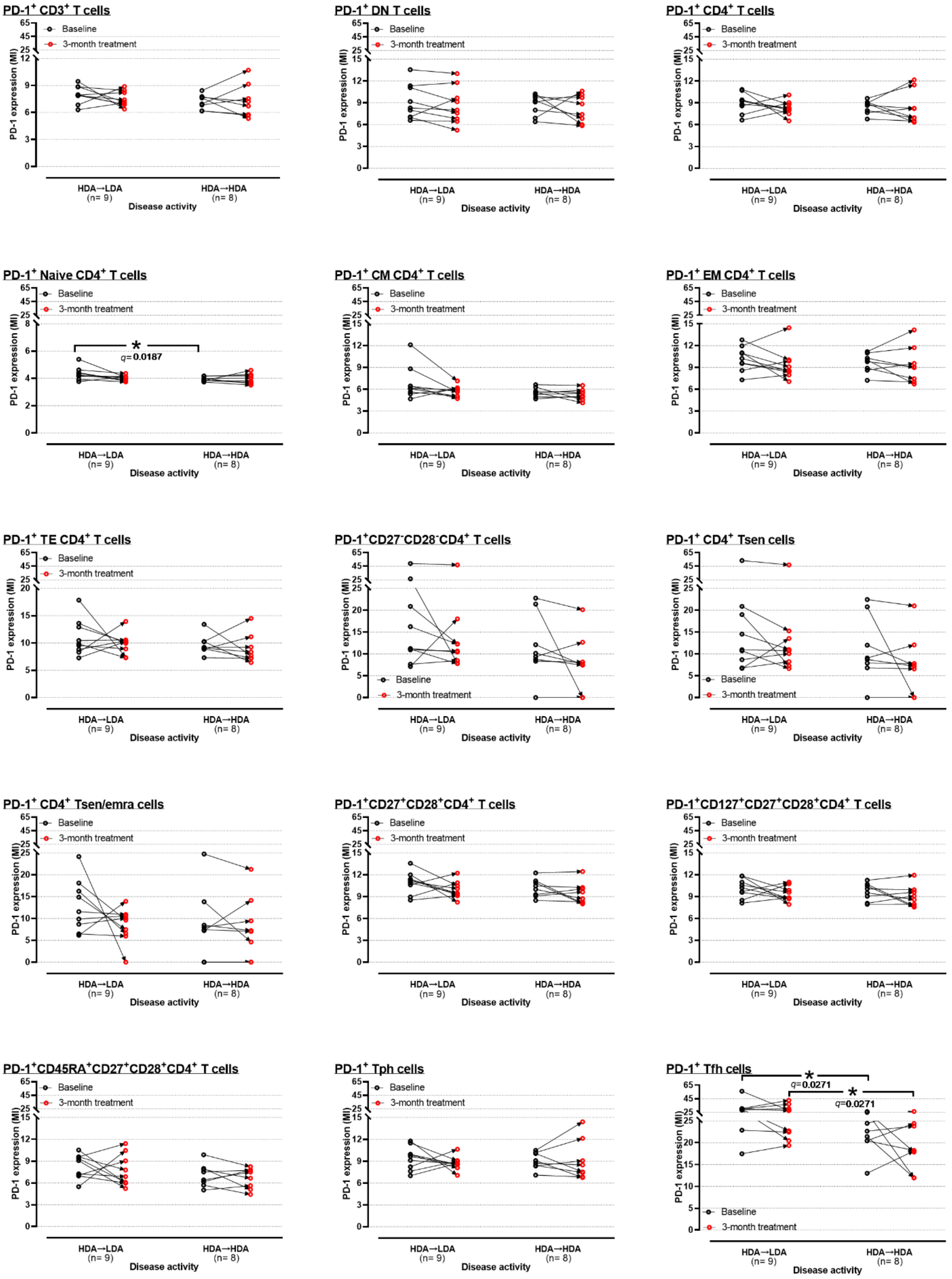

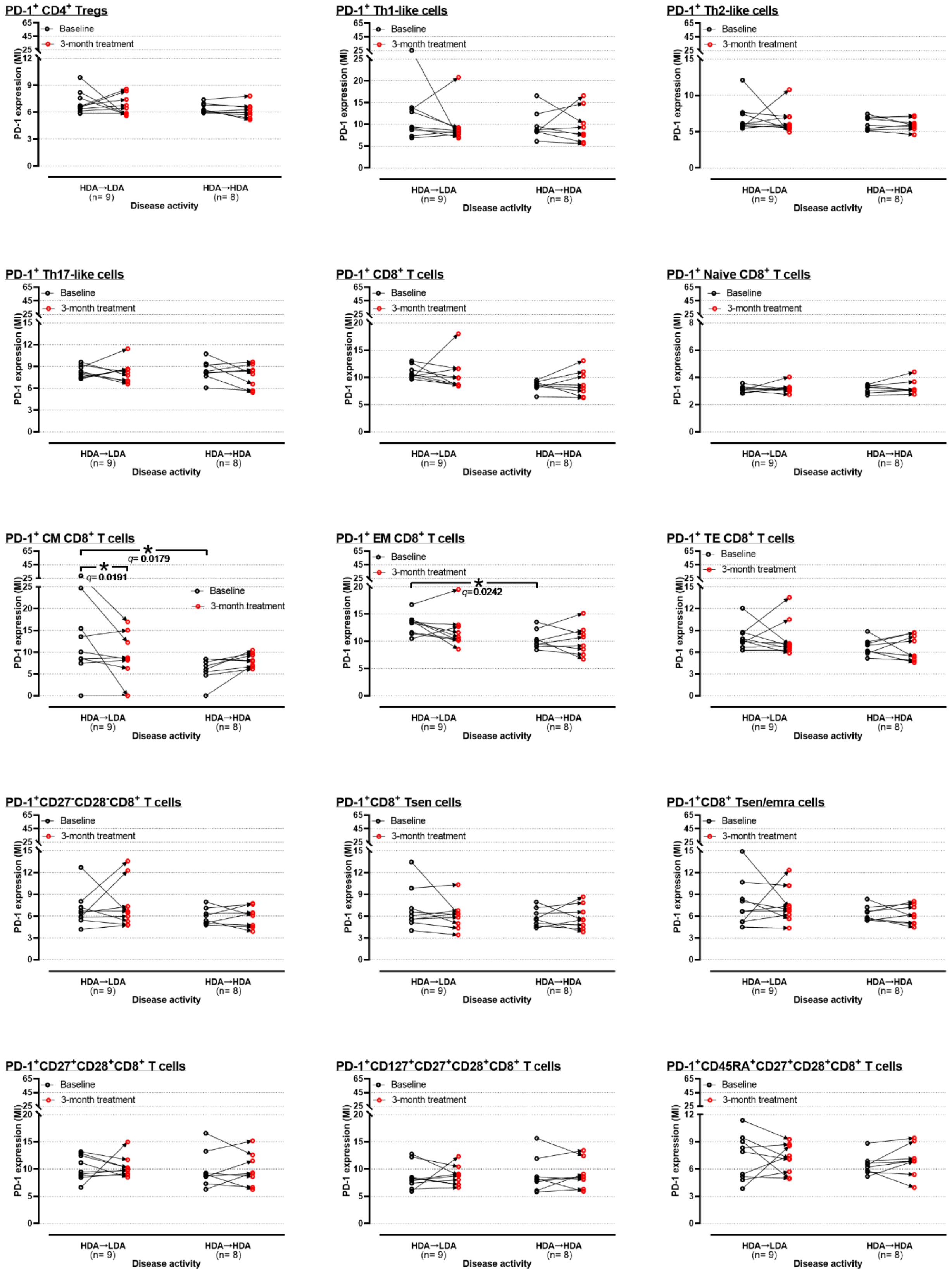
PD-1+ CD4+ Tregs
PD-1+ Th1-like cells
PD-1+ Th2-like cells
PD-1+ Th17-like cells
PD-1+ CD8+ T cells
PD-1+ Naive CD8+ T cells
PD-1+ CM CD8+ T cells
q= 0.0179
q= 0.0191
PD-1+ EM CD8+ T cells
q= 0.0242
PD-1+ TE CD8+ T cells
PD-1+CD27-CD28-CD8+ T cells
PD-1+CD8+ Tsen cells
PD-1+CD8+ Tsen/emra cells
PD-1+CD27+CD28+CD8+ T cells
PD-1+CD127+CD27+CD28+CD8+ T cells
PD-1+CD45RA+CD27+CD28+CD8+ T cells
Baseline
3-month treatment
PD-1 expression (MI)
HDA→LDA
(n= 9)
HDA→HDA
(n= 8)
Disease activity

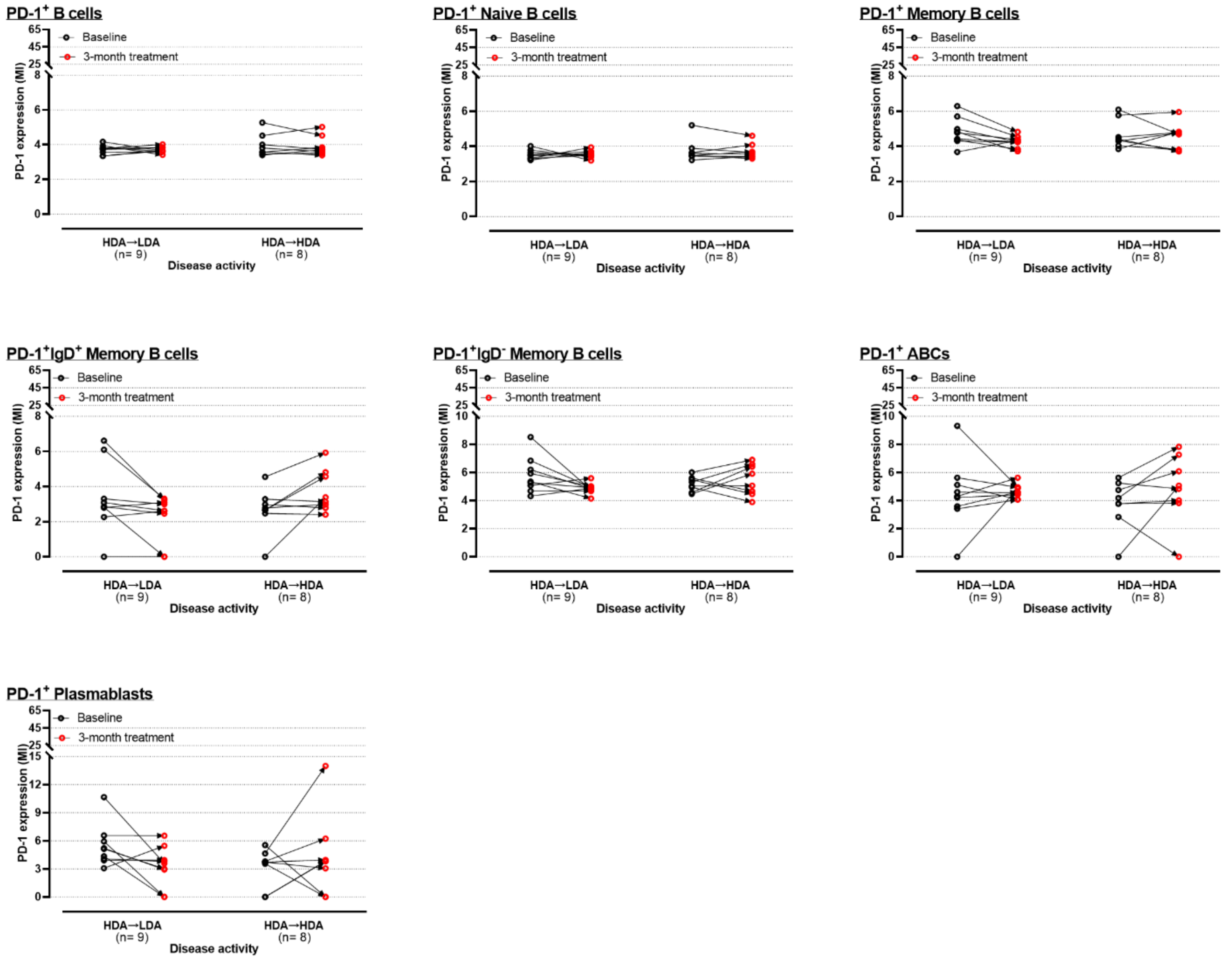

Supplementary Figure 21. Changes in PD-1 expression on PD-1$^+$ immune cell subpopulations/subsets after three months of treatments according to disease activity. The median intensity (MI) of PD-1 on PD-1$^+$ immune cells of (A) innate immunity, (B) innate and adaptive immunity and (C) adaptive immunity, in patients with IA at baseline and after 3 months of treatments, according to their disease activity after 3 months. Each circle corresponds to an individual patient (open black circles = baseline; open red circles = after 3 months). Groups were compared by performing two-way ANOVA corrected for multiple comparisons by false discovery rate (FDR) using the two-stage linear step-up procedure of Benjamini, Krieger and Yekutieli. (n: number of patients; MI: median intensity; IA: Inflammatory arthritis; NK: natural killer cells; DN: Double-Negative; CM: central memory; EM: effector memory; TE: terminal effector; Tsen: senescent T cells; Temra: effector memory T cells re-expressing CD45RA; Tregs: regulatory T cells; Th: T helper cells; Tfh: T follicular helper, Tph: T peripheral helper; ABC: age-associated B cells; DC: dendritic cells; mDC: myeloid dendritic cells; pDC: plasmacytoid dendritic cells; MAIT: mucosal-associated invariant T cells; iNKT: invariant natural killer T cells; ILCs: innate lymphoid cells; HDA: high disease activity (active disease); LDA: low disease activity (inactive disease); *, $q \leq 0.05$; **, $q \leq 0.01$)

1.2 Supplementary Tables

Supplementary Table 1. The antibody panel used for CyTOF analysis.

Metal	Target	Clone	Company
89Y	CD45	HI30	Standard BioTools
141Pr	CD196/CCR6	G034E3	Standard BioTools
143Nd	CD123/IL-3R	6H6	Standard BioTools
144Nd	CD19	HIB19	Standard BioTools
145Nd	CD4	RPA-T4	Standard BioTools
146Nd	CD8a	RPA-T8	Standard BioTools
147Sm	CD11c	Bu15	Standard BioTools
148Nd	CD16	3G8	Standard BioTools
149Sm	CD45RO	UCHL1	Standard BioTools
150Nd	CD45RA	HI100	Standard BioTools
151Eu	CD161	HP-3G10	Standard BioTools
152Sm	CD194/CCR4	L291H4	Standard BioTools
153Eu	CD25	BC96	Standard BioTools
154Sm	CD27	O323	Standard BioTools
155Gd	CD57	HCD57	Standard BioTools
156Gd	CD183/CXCR3	G025H7	Standard BioTools
158Gd	CD185/CXCR5	J252D4	Standard BioTools

160Gd	CD28	CD28.2	Standard BioTools
161Dy	CD38	HB-7	Standard BioTools
163Dy	CD56/NCAM	NCAM16.2	Standard BioTools
164Dy	TCRgd	B1	Standard BioTools
166Er	CD294	BM16	Standard BioTools
167Er	CD197/CCR7	G043H7	Standard BioTools
168Er	CD14	63D3	Standard BioTools
170Er	CD3	UCHT1	Standard BioTools
171Yb	CD20	2H7	Standard BioTools
172Yb	CD66b	G10F5	Standard BioTools
173Yb	HLA-DR	LN3	Standard BioTools
174Yb	IgD	IA6-2	Standard BioTools
175Lu	PD-1	EH12.2H7	Standard BioTools
176Yb	CD127/IL-7Ra	A019D5	Standard BioTools

Supplementary Table 2. The phenotypes of a total of 59 identified leukocyte subpopulations and their subsets.

A/A	subpopulation/subset	Phenotype
1	Granulocytes	$CD45^{lo}CD66b^{+}$
**2**	Neutrophils	$CD45^{lo}CD66b^{+}CD294^{-}CD16^{+}$
**3**	Eosinophils	$CD45^{lo}CD66b^{+}CD294^{+}CD16^{-}$
**4**	Basophils	$CD45^{+}CD66b^{-}CD19^{-}CD20^{-}CD3^{-}CD56^{-}HLA\text{-}DR^{-}CD11c^{-}CD123^{+}CD294^{+}$
**5**	$CD66b^{-}$ Neutrophils	$CD45^{lo}CD66b^{-}CD3^{-}CD19^{-}CD56^{-}HLA\text{-}DR^{-}CD123^{-}$
**6**	Monocytes	$CD45^{+}CD66b^{-}CD19^{-}CD20^{-}CD3^{-}CD56^{-}CD11c^{+}HLA\text{-}DR^{+}$
**7**	Classical monocytes	$CD14^{hi}CD38^{+}CD45^{+}CD66b^{-}CD19^{-}CD20^{-}CD3^{-}CD56^{-}CD11c^{+}HLA\text{-}DR^{+}$
**8**	Transitional monocytes	$CD14^{int}CD38^{lo/-}CD45^{+}CD66b^{-}CD19^{-}CD20^{-}CD3^{-}CD56^{-}CD11c^{+}HLA\text{-}DR^{+}$
**9**	Non-classical monocytes	$CD14^{-}CD38^{-}CD45^{+}CD66b^{-}CD19^{-}CD20^{-}CD3^{-}CD56^{-}CD11c^{+}HLA\text{-}DR^{+}$
**10**	NK cells	$CD45^{+}CD66b^{-}CD19^{-}CD20^{-}CD3^{-}CD14^{-}CD45RA^{+}CD123^{-}CD56^{+}$
**11**	Early NK cells	$CD57^{-}CD45^{+}CD66b^{-}CD19^{-}CD20^{-}CD3^{-}CD14^{-}CD45RA^{+}CD123^{-}CD56^{+}$
**12**	Late NK cells	$CD57^{+}CD45^{+}CD66b^{-}CD19^{-}CD20^{-}CD3^{-}CD14^{-}CD45RA^{+}CD123^{-}CD56^{+}$
**13**	ILC	$CD45^{+}CD3^{-}CD19^{-}CD56^{-}CD14^{-}CD16^{-}CD11c^{-}CD127^{+}$

**14**	ILC2	$CD161^{+}CD123^{-}TCR\gamma\delta^{-}CD294^{+}$ $CD45^{+}CD3^{-}CD19^{-}CD56^{-}CD14^{-}CD16^{-}CD11c^{-}CD127^{+}$
**15**	ILC3	$CD161^{+}CD123^{-}TCR\gamma\delta^{-}CD294^{-}CD45^{+}CD3^{-}CD19^{-}CD56^{-}CD14^{-}CD16^{-}CD11c^{-}CD127^{+}$
**16**	$CD3^{+}$ T cells	$CD45^{+}CD66b^{-}CD19^{-}CD20^{-}CD14^{-}CD11c^{-}CD3^{+}TCR\gamma\delta^{-}$
**17**	DN CD3+ T cells	$CD45^{+}CD66b^{-}CD19^{-}CD20^{-}CD14^{-}CD11c^{-}CD3^{+}TCR\gamma\delta^{-}CD4^{-}CD8^{-}$
**18**	$CD8^{+}$ T cells	$CD45^{+}CD66b^{-}CD19^{-}CD20^{-}CD14^{-}CD11c^{-}CD3^{+}TCR\gamma\delta^{-}CD4^{-}CD8^{+}CD161^{lo/-}$
**19**	Naïve $CD8^{+}$ T cells	$CCR7^{hi}CD45RO^{-}CD45RA^{+}CD45^{+}CD66b^{-}CD19^{-}CD20^{-}CD14^{-}CD11c^{-}CD3^{+}TCR\gamma\delta^{-}CD4^{-}CD8^{+}CD161^{lo/-}$
**20**	CM $CD8^{+}$ T cells	$CCR7^{hi}CD45RO^{+}CD45RA^{-}CD45^{+}CD66b^{-}CD19^{-}CD20^{-}CD14^{-}CD11c^{-}CD3^{+}TCR\gamma\delta^{-}CD4^{-}CD8^{+}CD161^{lo/-}$
**21**	EM $CD8^{+}$ T cells	$CCR7^{lo/-}CD27^{+}CD45^{+}CD66b^{-}CD19^{-}CD20^{-}CD14^{-}CD11c^{-}CD3^{+}TCR\gamma\delta^{-}CD4^{-}CD8^{+}CD161^{lo/-}$
**22**	TE $CD8^{+}$ T cells	$CCR7^{lo/-}CD27^{-}CD45^{+}CD66b^{-}CD19^{-}CD20^{-}CD14^{-}CD11c^{-}CD3^{+}TCR\gamma\delta^{-}CD4^{-}CD8^{+}CD161^{lo/-}$
**23**	$CD27^{-}CD28^{-}CD8^{+}$ T cells	$CD27^{-}CD28^{-}CD45^{+}CD66b^{-}CD19^{-}CD20^{-}CD14^{-}CD11c^{-}CD3^{+}TCR\gamma\delta^{-}CD4^{-}CD8^{+}CD161^{lo/-}$
**24**	$CD8^{+}$ Tsen	$CD57^{+}CD27^{-}CD28^{-}CD45^{+}CD66b^{-}CD19^{-}CD20^{-}CD14^{-}CD11c^{-}CD3^{+}TCR\gamma\delta^{-}CD4^{-}CD8^{+}CD161^{lo/-}$
**25**	$CD8^{+}$ Tsen-Temra	$CD45RA^{+}CD57^{+}CD27^{-}CD28^{-}CD45^{+}CD66b^{-}CD19^{-}CD20^{-}CD14^{-}CD11c^{-}CD3^{+}TCR\gamma\delta^{-}CD4^{-}CD8^{+}CD161^{lo/-}$

**26**	CD27^{+}CD28^{+}CD8^{+} T cells	CD27^{+}CD28^{+}CD45^{+}CD66b^{-}CD19^{-}CD20^{-}CD14^{-}CD11c^{-}CD3^{+}TCRγδ^{-}CD4^{-}CD8^{+}CD161$^{lo/-}$
**27**	CD127^{+}CD27^{+}CD28^{+}CD8^{+} T cells	CD127^{+}CD27^{+}CD28^{+}CD45^{+}CD66b^{-}CD19^{-}CD20^{-}CD14^{-}CD11c^{-}CD3^{+}TCRγδ^{-}CD4^{-}CD8^{+}CD161$^{lo/-}$
**28**	CD45RA^{+}CD27^{+}CD28^{+}CD8^{+} T cells	CD45RA^{+}CD27^{+}CD28^{+}CD45^{+}CD66b^{-}CD19^{-}CD20^{-}CD14^{-}CD11c^{-}CD3^{+}TCRγδ^{-}CD4^{-}CD8^{+}CD161$^{lo/-}$
**29**	CD4^{+} T cells	CD45^{+}CD66b^{-}CD19^{-}CD20^{-}CD14^{-}CD11c^{-}CD3^{+}TCRγδ^{-}CD4^{+}CD8^{-}
**30**	Naïve CD4^{+} T cells	CCR7^{hi}CD45RO^{-}CD45RA^{+}CD45^{+}CD66b^{-}CD19^{-}CD20^{-}CD14^{-}CD11c^{-}CD3^{+}TCRγδ^{-}CD4^{+}CD8^{-}
**31**	CM CD4^{+} T cells	CCR7^{hi}CD45RO^{+}CD45RA^{-}CD45^{+}CD66b^{-}CD19^{-}CD20^{-}CD14^{-}CD11c^{-}CD3^{+}TCRγδ^{-}CD4^{+}CD8^{-}
**32**	EM CD4^{+} T cells	CCR7$^{lo/-}$CD45RO^{+}CD45RA^{-}CD27^{+}CD45^{+}CD66b^{-}CD19^{-}CD20^{-}CD14^{-}CD11c^{-}CD3^{+}TCRγδ^{-}CD4^{+}CD8^{-}
**33**	TE CD4^{+} T cells	CCR7$^{lo/-}$CD45RO^{+}CD45RA^{-}CD27^{-}CD45^{+}CD66b^{-}CD19^{-}CD20^{-}CD14^{-}CD11c^{-}CD3^{+}TCRγδ^{-}CD4^{+}CD8^{-}
**34**	CD27^{-}CD28^{-}CD4^{+} T cells	CD27^{-}CD28^{-}CD45^{+}CD66b^{-}CD19^{-}CD20^{-}CD14^{-}CD11c^{-}CD3^{+}TCRγδ^{-}CD4^{+}CD8^{-}CD161$^{lo/-}$
**35**	CD4^{+} Tsen	CD57^{+}CD27^{-}CD28^{-}CD45^{+}CD66b^{-}CD19^{-}CD20^{-}CD14^{-}CD11c^{-}CD3^{+}TCRγδ^{-}CD4^{+}CD8^{-}CD161$^{lo/-}$
**36**	CD4^{+} Tsen-Temra	CD45RA^{+}CD57^{+}CD27^{-}CD28^{-}CD45^{+}CD66b^{-}CD19^{-}CD20^{-}CD14^{-}CD11c^{-}CD3^{+}TCRγδ^{-}CD4^{+}CD8^{-}CD161$^{lo/-}$

**37**	$CD27^{+}CD28^{+}CD4^{+}$ T cells	$CD27^{+}CD28^{+}CD45^{+}CD66b^{-}CD19^{-}CD20^{-}CD14^{-}CD11c^{-}CD3^{+}TCR\gamma\delta^{-}CD4^{+}CD8^{-}$
**38**	$CD127^{+}CD27^{+}CD28^{+}CD4^{+}$ T cells	$CD127^{+}CD27^{+}CD28^{+}CD45^{+}CD66b^{-}CD19^{-}CD20^{-}CD14^{-}CD11c^{-}CD3^{+}TCR\gamma\delta^{-}CD4^{+}CD8^{-}CD161^{lo/-}$
**39**	$CD45RA^{+}CD27^{+}CD28^{+}CD4^{+}$ T cells	$CD45RA^{+}CD27^{+}CD28^{+}CD45^{+}CD66b^{-}CD19^{-}CD20^{-}CD14^{-}CD11c^{-}CD3^{+}TCR\gamma\delta^{-}CD4^{+}CD8^{-}CD161^{lo/-}$
**40**	$CD4^{+}$ Tregs	$CCR4^{+}CD45RO^{+}CD45RA^{-}CD25^{hi}CD127^{lo/-}CD45^{+}CD66b^{-}CD19^{-}CD20^{-}CD14^{-}CD11c^{-}CD3^{+}TCR\gamma\delta^{-}CD4^{+}CD8^{-}$
**41**	Th1-like	$CXCR5^{-}CCR4^{-}CD45RO^{+}CD45RA^{-}CXCR3^{+}CCR6^{-}$ $CD45^{+}CD66b^{-}CD19^{-}CD20^{-}CD14^{-}CD11c^{-}CD3^{+}TCR\gamma\delta^{-}CD4^{+}CD8^{-}$
**42**	Th2-like	$CXCR5^{-}CCR4^{+}CD45RA^{-}CXCR3^{-}CCR6^{-}CD45^{+}CD66b^{-}CD19^{-}CD20^{-}CD14^{-}CD11c^{-}CD3^{+}TCR\gamma\delta^{-}CD4^{+}CD8^{-}$
**43**	Th17-like	$CXCR5^{-}CCR4^{+}CD45RA^{-}CXCR3^{-}CCR6^{+}CD45^{+}CD66b^{-}CD19^{-}CD20^{-}CD14^{-}CD11c^{-}CD3^{+}TCR\gamma\delta^{-}CD4^{+}CD8^{-}$
**44**	Tfh cells	$CXCR5^{+}CD57^{+}CD45^{+}CD66b^{-}CD19^{-}CD20^{-}CD14^{-}CD11c^{-}CD3^{+}TCR\gamma\delta^{-}CD4^{+}CD8^{-}$
**45**	Tph cells	$CXCR5^{-}CD45^{+}CD66b^{-}CD19^{-}CD20^{-}CD14^{-}CD11c^{-}CD3^{+}TCR\gamma\delta^{-}CD4^{+}CD8^{-}$
**46**	B cells	$CD45^{+}CD66b^{-}CD56^{-}CD14^{-}CD19^{+}CD3^{-}$
**47**	Naïve B cells	$CD27^{-}CD45^{+}CD66b^{-}CD56^{-}CD14^{-}CD19^{+}CD3^{-}$
**48**	Memory B cells	$CD27^{+}CD45^{+}CD66b^{-}CD56^{-}CD14^{-}CD19^{+}CD3^{-}$
**49**	IgD^{+} Memory B cells	$CD27^{+}IgD^{+}CD45^{+}CD66b^{-}CD56^{-}CD14^{-}CD19^{+}CD3^{-}$

**50**	IgD$^-$ Memory B cells	CD27$^+$IgD$^-$CD45$^+$CD66b$^-$CD56$^-$CD14$^-$CD19$^+$CD3$^-$
**51**	ABC	CD11c$^+$CXCR5$^-$CD45$^+$CD66b$^-$CD56$^-$CD14$^-$CD19$^+$CD3$^-$
**52**	Plasmablast	CD27$^+$CD38$^+$CD20$^-$CD45$^+$CD66b$^-$CD56$^-$CD14$^-$CD19$^+$CD3$^-$
**53**	DC	CD45$^+$CD66b$^-$CD19$^-$CD20$^-$CD3$^-$CD14$^-$HLA-DR$^+$
**54**	mDC	CD45$^+$CD66b$^-$CD19$^-$CD20$^-$CD3$^-$CD14$^-$HLA-DR$^+$CD123$^-$CD11c$^+$CD38$^+$
**55**	pDC	CD45$^+$CD66b$^-$CD19$^-$CD20$^-$CD3$^-$CD14$^-$HLA-DR$^+$CD123$^+$CD11c$^-$
**56**	γδ T cells	CD45$^+$CD66b$^-$CD19$^-$CD20$^-$CD14$^-$CD11c$^-$CD3$^+$CD4$^-$CD8$^-$TCRγδ$^+$
**57**	MAIT/iNKT cells	CD45$^+$CD66b$^-$CD19$^-$CD20$^-$CD14$^-$CD11c$^-$CD3$^+$CD4$^-$CD28$^+$CD161^{hi}
**58**	Activated MAIT/iNKT cells	CD127$^+$CCR6$^+$CD45$^+$CD66b$^-$CD19$^-$CD20$^-$CD14$^-$CD11c$^-$CD3$^+$CD4$^-$CD28$^+$CD161^{hi}
**59**	Memory MAIT/iNKT cells	CD45RO$^+$CCR7$^-$CD45$^+$CD66b$^-$CD19$^-$CD20$^-$CD14$^-$CD11c$^-$CD3$^+$CD4$^-$CD28$^+$CD161^{hi}

NK: natural killer cells; DN: Double-Negative; CM: central memory; EM: effector memory; TE: terminal effector; Tsen: senescent T cells; Temra: effector memory T cells re-expressing CD45RA; Tregs: regulatory T cells; Th: T helper cells; Tfh: T follicular helper, Tph: T peripheral helper; ABC: age-associated B cells; DC: dendritic cells; mDC: myeloid dendritic cells; pDC: plasmacytoid dendritic cells; MAIT: mucosal-associated invariant T cells; iNKT: invariant natural killer T cells; ILCs: innate lymphoid cells

Supplementary Table 3. Frequencies of PD-1^{+}-circulating immune cell types of adaptive immunity in patients with PsA, RA, and HC. (p-values were determined by unpaired t-test or Mann–Whitney U test).

Subpopulations/subsets of PD-1^{+-}circulating immune cells (%)	Mean ± SEM			
	**HC** (n=13)	**Seropositive RA** (n=9)	**Seronegative RA** (n=8)	**PsA** (n=9)
**CD3^{+} T cells**	28.22 ± 1.86	31.61 ± 2.58	31.06 ± 2.07	32.35 ± 3.16
DN	26.80 ± 2.42	31.15 ± 2.78	29.18 ± 5.44	37.81 ± 5.44
**CD8^{+} T cells**	24.78 ± 2.09	29.75 ± 2.24	29.65 ± 3.82	25.17 ± 2.64
Naïve	2.80 ± 0.20	2.92 ± 0.52	3.90 ± 0.59	5.97 ± 2.00
CM	12.47 ± 2.51	14.95 ± 3.44	13.68 ± 2.57	16.10 ± 2.95
EM	47.31 ± 3.42	53.53 ± 2.80	50.33 ± 2.59	51.00 ± 3.36
TE	23.60 ± 3.52	22.72 ± 3.31	24.21 ± 4.18	24.78 ± 5.20
CD27^{-}CD28^{-}	26.17 ± 3.83	25.14 ± 3.53	28.69 ± 4.97	25.52 ± 5.47
Tsen	25.51 ± 3.96	23.14 ± 3.88	27.15 ± 5.62	27.59 ± 6.97
Tsen-Temra	19.06 ± 3.21	17.08 ± 3.21	19.03 ± 4.01	21.32 ± 6.72
CD27^{+}CD28^{+}	25.55 ± 2.23	**34.79 ± 3.36§**	33.13 ± 4.30	27.23 ± 2.85
CD127^{+}CD27^{+}CD28^{+}	22.43 ± 2.30	**31.24 ± 3.63§**	30.23 ± 4.33	23.81 ± 3.07
CD45RA^{+}CD27^{+}CD28^{+}	10.60 ± 1.56	**18.04 ± 2.77§**	15.58 ± 3.24	12.74 ± 2.25
**CD4^{+} T cells**	26.02 ± 2.08	28.38 ± 3.34	27.91 ± 1.79	29.76 ± 3.01
Naïve	2.52 ± 0.29	2.52 ± 0.23	2.94 ± 0.43	5.12 ± 1.29
CM	19.42 ± 1.26	20.84 ± 2.12	19.34 ± 1.72	23.61 ± 3.26
EM	43.73 ± 1.97	43.89 ± 3.21	46.01 ± 2.14	47.87 ± 3.94
TE	47.55 ± 4.70	46.74 ± 4.76	51.87 ± 4.34	45.45 ± 3.89
CD27^{-}CD28^{-} cells	46.15 ± 6.61	45.19 ± 7.61	45.38 ± 6.04	52.10 ± 10.34
Tsen	43.42 ± 7.45	40.93 ± 7.73	51.13 ± 6.38	53.39 ± 10.59
Tsen-Temra	41.24 ± 7.37	32.29 ± 7.86	52.01 ± 11.02	59.94 ± 11.97
CD27^{+}CD28^{+}	17.52 ±1.54	21.20 ± 2.36	21.02 ± 1.44	23.11 ± 2.41
CD127^{+}CD27^{+}CD28^{+}	15.85 ± 1.58	18.86 ± 2.05	19.29 ± 1.36	20.81 ± 2.31
CD45RA^{+}CD27^{+}CD28^{+}	3.13 ±0.34	**5.58 ± 1.12§**	**5.82 ± 1.10$^{\#\#}$**	**7.52 ± 1.72****
Tph	21.93 ± 2.06	23.86 ± 3.21	22.61 ± 1.95	24.03 ± 2.75
Tfh	49.24 ± 4.74	**67.95 ± 5.48§**	57.87 ± 3.11	**68.30 ± 5.55***
Treg	28.84 ± 3.57	28.62 ± 3.20	28.32 ± 3.23	31.82 ± 2.48

Th1-like	49.48 ± 3.54	53.82 ± 5.19	50.87 ± 3.44	50.86 ± 4.52
Th2-like	25.10 ± 1.15	22.72 ± 2.61	28.43 ± 2.05	28.86 ± 2.92
Th17-like	38.07 ± 2.28	34.62 ± 3.66	38.70 ± 2.83	40.42 ± 3.51
**B cells**	2.78 ± 0.30	4.04 ± 0.94	3.01 ± 0.56	4.76 ± 1.45
Naïve	2.93 ± 0.25	4.10 ± 1.29	3.48 ± 0.63	5.17 ± 0.58
Memory	1.86 ± 0.27	**4.81 ± 1.26**§	1.91 ± 0.40	3.55 ± 1.15
IgD^+ Memory	4.53 ± 0.71	**1.84 ± 3.54**§§	5.08 ± 1.23	7.94 ± 2.25
IgD^- Memory	1.17 ± 0.15	**3.54 ± 1.00**§§	1.18 ± 0.16	2.79 ± 1.02
ABC	14.51 ± 1.75	**24.90 ± 3.00**§§	21.91 ± 4.32	21.92 ± 3.57
Plasmablast	1.40 ± 0.54	2.72 ± 1.09	2.18 ± 1.39	6.23 ± 4.25

n: number of patients; HC: healthy control; PsA: psoriatic arthritis; RA: rheumatoid arthritis; PD-1: programmed cell death-1; DN: Double-negative; CM: central memory; EM: effector memory; TE: terminal effector; Tsen: senescent T cells; Temra: effector memory T cells re-expressing CD45RA; Tregs: regulatory T cells; Th: T helper cells, Tfh: T follicular helper, Tph: T peripheral helper, MI: median intensity; ABC: Age-associated B cells (* $p\leq 0.05$, ** $p<0.01$, HC vs PsA; ## $p<0.01$, HC vs seronegative RA; § $p\leq 0.05$, §§ $p\leq 0.01$, HC vs seropositive RA).

Supplementary Table 4. Median intensity of the PD-1 molecule on PD-1^{+}-circulating immune cell types of adaptive immunity in patients with PsA, RA, and HC. (*p*-values were determined by unpaired t-test or Mann–Whitney U test).

**Subpopulations/subsets of PD-1^{+}-circulating immune cells (MI)**	**Mean ± SEM**			
	**HC** (n=13)	**Seropositive RA** (n=9)	**Seronegative RA** (n=8)	**PsA** (n=9)
**CD3^{+} T cells**	6.72 ± 0.26	**7.77 ± 0.44**§	7.13 ± 0.40	7.11 ± 0.38
DN	7.35 ± 0.73	9.01 ± 0.77	7.31 ±0.69	7.98 ± 0.66
**CD8^{+} T cells**	8.56 ± 0.42	9.80 ± 0.63	9.69 ± 0.67	9.56 ± 0.51
Naïve	3.16 ± 0.05	3.13 ± 0.08	3.26 ± 0.11	3.17 ± 0.07
CM	12.47 ± 3.06	12.14 ± 2.30	16.66 ± 8.61	8.21 ± 1.53
EM	9.94 ± 0.61	**12.14 ± 0.69**§	10.69 ± 0.75	11.09 ± 0.91
TE	6.52 ± 0.38	6.94 ± 0.46	7.08 ± 0.35	7.85 ± 0.65
$CD27^{-}CD28^{-}$ cells	6.03 ± 0.43	5.99 ± 0.31	6.57 ± 0.44	6.87 ± 0.90
Tsen	5.84 ± 0.43	5.75 ± 0.36	6.38 ± 0.44	7.08 ± 1.04
Tsen-Temra	6.49 ± 0.37	6.22 ± 0.29	6.34 ± 0.34	7.78 ± 1.11
$CD27^{+}CD28^{+}$	9.03 ± 0.67	**12.02 ± 0.82**§	8.82 ± 0.35	8.02 ± 0.41
$CD127^{+}CD27^{+}CD28^{+}$	7.83 ± 0.54	**10.70 ± 090**§§	7.63 ± 0.21	6.92 ± 0.35
$CD45RA^{+}CD27^{+}CD28^{+}$	5.78 ± 0.43	**8.92 ± 0.54**§§	5.72 ± 0.38	5.46 ± 0.43
**CD4^{+} T cells**	7.75 ± 0.33	**8.81 ± 0.46**§	8.34 ± 0.62	8.13 ± 0.52
Naïve	3.99 ± 0.08	4.20 ± 0.18	3.78 ± 0.08	4.02 ± 0.09
CM	5.64 ± 0.22	6.21 ± 0.38	5.30 ± 0.27	6.09 ± 0.78
EM	8.42 ± 0.30	**9.74 ± 0.50**§	9.15 ± 0.66	9.60 ± 0.75
TE	8.78 ± 0.79	6.61 ± 0.46	6.31 ± 0.30	8.44 ± 0.45
$CD27^{-}CD28^{-}$ cells	9.80 ± 0.98	13.38 ± 2.48	10.78 ± 2.01	14.75 ±4.77
Tsen	8.30 ± 1.07	11.99 ± 2.11	10.53 ± 1.82	13.59 ± 5.25
Tsen-Temra	7.69 ± 0.88	8.95 ± 1.81	10.24 ± 1.12	11.56 ±2.80
$CD27^{+}CD28^{+}$	9.28 ± 0.25	**10.85 ± 0.45**§§	10.24 ± 0.68	9.82 ± 0.61
$CD127^{+}CD27^{+}CD28^{+}$	8.85 ± 0.24	**10.22 ± 0.44**§§	9.98 ± 0.74	9.12 ± 0.50
$CD45RA^{+}CD27^{+}CD28^{+}$	6.46 ± 0.28	**7.96 ± 0.58**§	7.88 ± 0.88	7.01 ± 0.62
Tph	8.33 ± 0.39	9.47 ± 0.52	8.86 ± 0.67	8.52 ± 0.58
Tfh	23.08 ± 2.22	27.38 ± 4.05	22.11 ± 2.58	25.28 ± 1.43
Treg	28.84 ± 3.57	28.62 ± 3.20	28.32 ± 3.23	31.82 ± 2.48

Th1-like	8.23 ± 0.50	**12.30 ± 1.93**§	8.73 ± 1.14	8.82 ±1.00
Th2-like	5.84 ± 0.11	6.58 ± 0.73	6.54 ± 0.41	6.17 ± 0.30
Th17-like	7.93 ± 0.39	7.65 ± 0.35	8.04 ± 0.50	8.62 ± 0.46
**B cells**	3.60 ± 0.06	**4.07 ± 0.22**§	3.51 ± 0.08	3.73 ± 0.09
Naive	3.43 ±0.04	3.78 ± 0.21	3.43 ± 0.09	3.59 ± 0.08
Memory	4.13 ± 0.08	**5.07 ± 0.32**§	4.05 ± 0.18	4.35 ± 0.14
IgD^+ Memory	2.83 ± 0.28	2.44 ± 0.68	2.46 ± 0.36	2.54 ± 0.33
IgD^- Memory	5.20 ± 0.22	5.76 ± 0.45	5.18 ± 0.42	4.95 ± 0.17
ABC	4.61 ± 0.28	5.53 ±0.55	3.77 ± 0.30	5.56 ± 0.72
Plasmablast	2.95 ± 0.68	3.05 ± 0.61	2.27 ± 0.68	2.66 ± 0.68

*n: number of patients; HC: healthy control; PsA: psoriatic arthritis; RA: rheumatoid arthritis; PD-1: programmed cell death-1; MI: median intensity; DN: Double-negative; CM: central memory; EM: effector memory; TE: terminal effector; Tsen: senescent T cells; Temra: effector memory T cells re-expressing CD45RA; Tregs: regulatory T cells; Th: T helper cells; Tfh: T follicular helper, Tph: T peripheral helper, MI: median intensity; ABC: Age-associated B cells (§ $p \leq 0.05$, §§ $p \leq 0.01$, HC vs seropositive RA).

Supplementary Table 5. Expression of PD-1^{+} on leukocyte subpopulations and their subsets involved in innate immunity (P values were determined by unpaired t-test or Mann–Whitney U test)

	Mean ± SEM			
**PD-1^{+}- expressing cells**	**HC** (n=13)	**Seropositive RA** (n=9)	**Seronegative RA** (n=8)	**PsA** (n=9)
**Granulocytes**				
%	5.11 ± 0.62	3.86 ± 0.55	4.48 ± 0.90	10.95 ± 5.63
MI	5.02 ± 0.19	4.73 ± 0.25	**4.28 ± 0.08$^{\#\#}$**	4.80 ± 0.29
Neutrophils				
%	5.18 ± 0.68	4.46 ± 0.47	6.47 ± 1.65	13.50 ± 6.48
MI	3.00 ± 0.02	2.96 ± 0.02	3.02 ± 0.04	**3.49 ± 0.33***
Basophils				
%	1.45 ± 0.15	1.57 ± 0.17	1.70 ± 0.36	5.06 ± 2.27
MI	3.38 ± 0.07	3.44 ± 0.08	3.61 ± 0.11	3.57 ± 0.11
Eosinophils				
%	66.67 ± 3.53	73.50 ± 3.98	59.16 ± 6.53	67.61 ± 2.45
MI	7.99 ± 0.97	11.70 ± 3.03	6.78 ± 1.15	7.50 ± 0.86
CD66b^{-} Neutrophils				
%	1.78 ± 0.19	3.48 ± 1.18	2.82 ± 0.80	10.27 ± 6.45
MI	4.06 ± 0.06	4.79 ± 0.71	4.40 ± 0.34	7.82 ± 2.44
**Monocytes**				
%	4.71 ± 0.47	6.82 ± 1.65	6.39 ± 1.14	10.29 ± 3.72
MI	3.01 ± 0.02	3.07 ± 0.06	3.06 ± 0.03	**3.27 ± 0.12***
Classical				
%	5.04 ± 0.52	7.23 ± 1.79	6.64 ± 1.20	10.52 ± 3.75
MI	3.05 ± 0.02	3.11 ± 0.07	3.11 ± 0.03	**3.30 ± 0.12***
Transitional				
%	4.10 ± 0.37	4.79 ± 0.67	5.93 ± 1.49	**9.81 ± 3.63***
MI	3.06 ± 0.02	3.13 ± 0.07	2.99 ± 0.05	**3.35 ± 0.10****
Non-classical				
%	1.91 ± 0.15	2.43 ± 0.29	3.24 ± 0.76	**5.72 ± 2.61***
MI	3.33 ± 0.05	3.29 ± 0.06	3.29 ± 0.08	**3.63 ± 0.10****

**NK**				
%	1.43 ± 0.17	1.70 ± 1.01	1.61 ± 0.34	3.82 ± 1.80
MI	3.90 ± 0.22	4.22 ± 0.31	3.57 ± 0.10	3.56 ± 0.10
Early				
%	1.40 ± 0.13	1.54 ± 0.23	2.06 ± 0.53	4.06 ± 1.94
MI	3.55 ± 0.05	3.93 ± 0.32	3.53 ± 0.09	3.56 ± 0.09
Late				
%	1.59 ± 0.19	1.58 ± 1.02	1.27 ± 0.27	3.48 ± 1.67
MI	4.43 ± 0.49	5.69 ± 1.12	3.64 ± 0.14	3.63 ± 3.35
**ILC**				
%	23.68 ± 2.95	27.95 ± 5.41	21.88 ± 1.90	26.14 ± 4.88
MI	8.69 ± 1.88	7.20 ± 1.12	6.78 ± 1.27	6.75 ± 1.80
ILC2				
%	0.18 ± 0.18	1.83 ± 1.40	**2.42 ± 1.27**$^{\#}$	4.66 ± 3.18
MI	0.40 ± 0.40	1.36 ± 0.96	6.76 ± 4.45	2.25 ± 0.92
ILC3				
%	17.66 ± 4.03	13.18 ± 4.77	19.15 ± 8.14	15.65 ± 5.94
MI	11.27 ± 2.85	5.44 ± 1.92	33.21 ± 21.35	11.21 ± 2.70

n: number of patients; HC: healthy control; RA: rheumatoid arthritis; PsA: psoriatic arthritis; MI: Median intensity; NK: natural killer cells; ILC2: innate lymphoid cells type 2; ILC3: innate lymphoid cells type 3; PD-1: programmed cell death-1 (*p≤ 0.05, ** p≤ 0.01, HC vs PsA; $^{\#}$ p≤ 0.05 and $^{\#\#}$ p≤ 0.01, HC vs seronegative RA)

Supplementary Table 6. Percentages and expression of PD-1 on leukocyte subpopulations and their subsets involved in both innate and adaptive immunity. **(**P values were determined by unpaired t-test or Mann–Whitney U test)

**Subpopulations/ subsets of PD-1^{+-} circulating immune cells**	**Mean ± SEM**			
	**HC** (n=13)	**Seropositive RA** (n=9)	**Seronegative RA** (n=8)	**PsA** (n=9)
**DC**				
%	0.75 ± 0.09	0.70 ± 0.06	1.49 ± 0.41	**3.53 ± 1.97***
MI	4.97 ± 0.15	5.08 ± 0.25	5.11 ± 0.23	4.90 ± 0.14
Plasmacytoid				
%	2.24 ± 0.58	1.23 ± 0.39	3.20 ± 1.09	5.94 ± 2.89
MI	3.54 ± 0.07	3.16 ± 0.65	3.43 ± 0.15	**3.91 ± 0.13***
Myeloid				
%	2.26 ± 0.22	2.11 ± 0.21	3.55 ± 0.94	6.81 ± 3.43
MI	3.38 ± 0.08	3.56 ± 0.17	3.48 ± 0.13	**3.70 ± 0.12***
**MAIT/iNKT**				
%	28.97 ± 3.16	23.84 ± 3.98	30.04 ± 4.70	35.95 ± 6.74
MI	5.75 ± 0.23	5.73 ± 0.41	6.28 ± 0.83	6.23 ± 0.52
Memory				
%	21.60 ± 2.82	18.14 ± 3.82	23.42 ± 4.17	29.48 ± 6.40
MI	6.90 ± 0.28	7.74 ± 0.59	8.35 ± 1.66	7.54 ± 0.54
Activated				
%	29.88 ± 3.43	21.16 ± 2.66	30.46 ± 5.92	41.18 ± 8.12
MI	7.94 ± 0.49	10.21 ± 1.92	8.25 ± 0.87	10.01 ± 1.24
**γδ T cells**				
%	25.63 ± 2.79	26.35 ± 5.75	27.35 ± 2.79	22.42 ± 4.64
MI	5.47 ± 0.35	7.20 ± 0.83	6.31 ± 0.35	5.24 ± 0.36

n: number of patients; HC: healthy control; PsA: psoriatic arthritis; RA: rheumatoid arthritis; MI: Median intensity; DCs: dendritic cells; MAIT: mucosal-associated invariant T cells; iNKT: invariant natural killer T cells; PD-1: programmed cell death-1 (* $p \leq 0.05$, HC vs PsA).

Supplementary Table 7. Significant correlations of PD-1 expression levels (MI) of circulating PD-1$^+$ immune cells at baseline with CRP and/or ESR in bDMARD-naïve **A**, seropositive RA, **B**, seronegative RA, and **C,** PsA patients. Correlation coefficients between the variables were calculated according to Spearman's rank correlation coefficient (rho)

**Subpopulations/subsets of PD-1$^+$-circulating immune cells**	**CRP**		**ESR**	
	Rho	P value	Rho	P value
**A. SEROPOSITIVE RA**				
**Innate Immunity**				
Neutrophils	0.636	NS	**0.714**	**0.031**
ILC	**-0.783**	**0.017**	**-0.678**	**0.045**
**Innate & Adaptive Immunity**				
DC	0.533	NS	**0.678**	**0.045**
**Adaptive Immunity**				
CD4$^+$ T cells	0.400	NS	**0.678**	**0.045**
Naive	0.133	NS	**0.753**	**0.019**
Tregs	0.400	NS	**0.854**	**0.003**
CD8$^+$ T cells				
EM	0.200	NS	**0.073**	**0.035**
B cells				
Memory	**-0.700**	**0.043**	-0.536	NS
**B. SERONEGATIVE RA**				
**Innate Immunity**				
ILC	**-0.738**	**0.046**	**-0.833**	**0.015**
NK	**-0.755**	**0.031**	-0.611	NS
Late	-0.695	0.056	**-0.743**	**0.035**
**Adaptive Immunity**				
CD4$^+$ Tregs	**0.738**	**0.046**	0.595	NS
**C. PsA**				
**Innate Immunity**				
Monocytes	-0.483	NS	**-0.700**	**0.043**
Classical	-0.536	NS	**-0.720**	**0.029**
Eosinophils	**-0.800**	**0.014**	**-0.717**	**0.037**
**Innate & Adaptive Immunity**				
DC	**-0.717**	**0.037**	-0.367	NS

**Adaptive Immunity**				
$CD3^+$ T cells	-0.200	NS	**-0.683**	**0.050**
$CD4^+$ T cells	-0.383	NS	**-0.733**	**0.031**
TE	-0.350	NS	**-0.750**	**0.025**
Tph	-0.417	NS	**-0.683**	**0.050**
$CD27^+CD28^+$	-0.200	NS	**-0.683**	**0.050**
$CD27^-CD28^-$	-0.600	NS	**-0.733**	**0.031**
Tsen	-0.633	NS	**-0.783**	**0.017**
Tsen/Temra	**-0.700**	**0.043**	**-0.800**	**0.014**
$CD8^+$ T cells				
Naïve	**-0.700**	**0.043**	-0.667	NS
$CD27^+CD28^+$	**0.783**	**0.017**	0.350	NS

RA: rheumatoid arthritis; PsA: psoriatic arthritis; MI: median intensity; CRP: C-reactive protein; ESR: erythrocyte sedimentation rate; PD-1: programmed cell death-1; ILC: innate lymphoid cells; DC: dendritic cells; Tregs: regulatory T cells; EM: effector memory; NK: natural killer cells; TE: terminal effector; Th: T helper cells; Tsen: senescent T cells; Temra: effector memory T cells re-expressing CD45RA

Supplementary Table 8. Significant correlations of the PD-1 expression levels (MI) of circulating PD-1^{+} immune cells with CRP and/or ESR in IA patients who **A,** remain active 3 months post-therapy (n=8) and **B,** who became inactive 3 months post-therapy**.** Correlation coefficients between the variables were calculated according to Spearman's rank correlation coefficient (rho)

**Subpopulations/subsets of PD-1^{+}-circulating immune cells**	**CRP**		**ESR**	
	Rho	P value	Rho	P value
**A. Active disease 3 months post-therapy**				
**Innate Immunity**				
ILC				
ILC2	-0.655	NS	**-0.741**	**0.035**
Granulocytes				
Neutrophils	-0.262	NS	**-0.778**	**0.023**
Monocytes	-0.167	NS	**-0.743**	**0.035**
Classical	-0.167	NS	**-0.743**	**0.035**
**Innate & Adaptive Immunity**				
DC				
Plasmacytoid	-0.262	NS	**-0.790**	**0.020**
**Adaptive Immunity**				
CD4^{+} T cells				
CD27^{-}CD28^{-}	**-0.970**	**0.001**	-0.259	NS
Tsen	**-0.970**	**0.001**	-0.452	NS
Tsen/Temra	**-0.731**	**0.040**	0.078	NS
**B. Inactive disease 3 months post-therapy**				
**Innate Immunity**				
Eosinophils	**0.753**	**0.019**	0.708	NS
**Adaptive Immunity**				
DN T cells	**0.686**	**0.047**	0.100	0.810
CD4^{+} T cells				
EM	-0.510	NS	**-0.800**	**0.014**
Th2-like	-0.268	NS	**-0.700**	**0.043**
Th17-like	-0.510	NS	**-0.850**	**0.006**
ABC cells	0.192	NS	**0.700**	**0.043**

IA: inflammatory arthritis; MI: median intensity; CRP: C-reactive protein; ESR: erythrocyte sedimentation rate; PD-1: programmed cell death-1; ILC: innate lymphoid cells; DC: dendritic cells; DN: Double-negative; Tsen: senescent T cells; Temra: effector memory T cells re-expressing CD45RA; EM: effector memory; Th: T helper cells; ABC: age-associated B cells
